# Supplementary material for: Exploitation of the Apoptosis-Primed State of MYCN-Amplified Neuroblastoma to Develop a Potent and Specific Targeted Therapy Combination
Source: Cancer Cell. 2016 Feb 8;29(2):159–72. doi: 10.1016/j.ccell.2016.01.002 (PMC4749542; doi:10.1016/j.ccell.2016.01.002)
Supplement: Document S1. Supplemental Experimental Procedures, Figures S1–S7, and Tables S1, S2 [file mmc1.pdf]

Cancer Cell, Volume *29*

Supplemental Information

Exploitation of the Apoptosis-Primed State of *MYCN*-Amplified Neuroblastoma to Develop a Potent and Specific Targeted Therapy Combination

Jungoh Ham, Carlotta Costa, Renata Sano, Timothy L. Lochmann, Erin M. Sennott, Neha U. Patel, Anahita Dastur, Maria Gomez-Caraballo, Kateryna Krytska, Aaron N. Hata, Konstantinos V. Floros, Mark T. Hughes, Charles T. Jakubik, Daniel A.R. Heisey, Justin T. Ferrell, Molly L. Bristol, Ryan J. March, Craig Yates, Mark A. Hicks, Wataru Nakajima, Madhu Gowda, Brad E. Windle, Mikhail G. Dozmorov, Mathew J. Garnett, Ultan McDermott, Hisashi Harada, Shirley M. Taylor, Iain M. Morgan, Cyril H. Benes, Jeffrey A. Engelman, Yael P. Mossé, and Anthony C. Faber

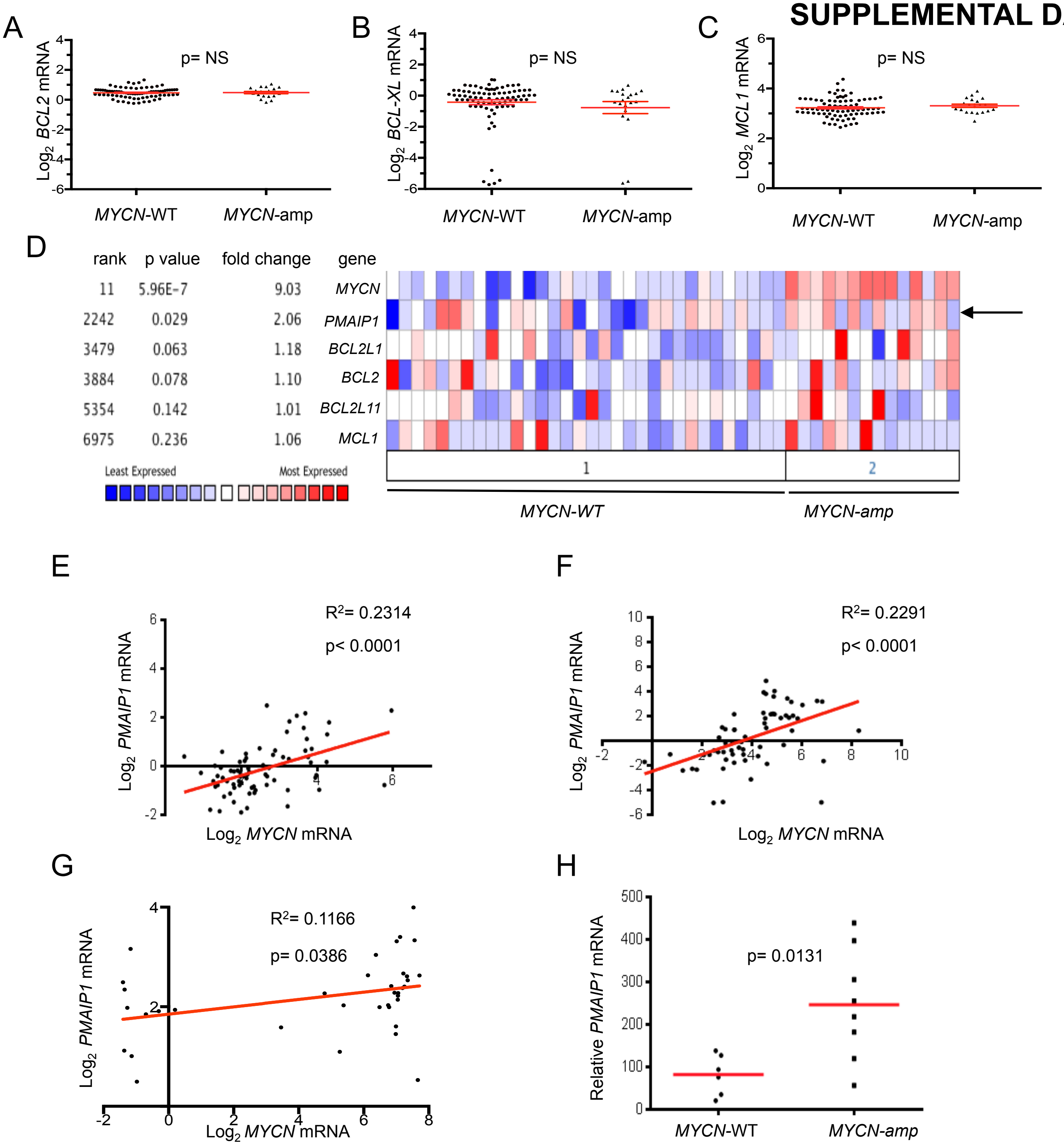

Figure S1 (related to Figure 1) Gene expression data parsing reveal *MYCN*-amplified neuroblastoma have high NOXA expression.

(A-C) RNA expression from the Wang dataset (Wang et al., 2006) for *MYCN*-WT neuroblastoma tumors (n=81) and *MYCN*-amplified (amp) neuroblastoma tumors (n=20). NS= not significant by non-parametric Mann-Whitney U-Test. Red lines are mean, error bars are -/+S.E.M. Y axis = Log_2 median-centered.

(D) Comparison of BCL-2 family members in *MYCN* wild-type (WT) and *MYCN*-amplified (amp) neuroblastomas (Janoueix-Lerosey et al., 2008) The Oncomine™ Platform (Life Technologies, Ann Arbor, MI) was used for analysis and visualization. Arrow indicates NOXA (*PMAIP1*). Data is Log_2 median-centered.

(E, F*)* Linear regression analysis between *MYCN* and NOXA (*PMAIP1*) expression in two medulloblastoma tumor sets (Kool et al., 2008; Robinson et al., 2012) (R^2=0.2314 for Robinson et al. (E), R^2=0.2291 for Kool et al. (F), p value <0.0001 for both sets). Data from Oncomine and is Log_2 median-centered.

(G) Linear regression analysis between *MYCN* and NOXA (*PMAIP1*) expression in neuroblastoma cell lines (Garnett et al. 2012). R^2=0.1166, p value = 0.0386. Data from Oncomine and is Log_2 median-centered.

(H) *MYCN*-WT and *MYCN*-amp neuroblastoma cell lines used in this study were assayed for NOXA RNA expression by qRTPCR. Student t-test indicated statistical difference between the two groups (p =0.0131).

Table S1 (related to Figure 1) *MYCN* amplified cancer cells are sensitive to ABT-263 (modified from cancerRxgene.org)

Drug Name	gene	sample (n)	mutant (n)	p value	IC50 Effect	Slope Effect	Mean (WT)	Mean (mutant)	Q Value
ABT-263	MYCN	661	26	0.000204583	0.064853587	0.0916665	2.28374	-0.105377	0.0112351
IPA-3	MYCN	665	25	0.235236	0.278877836	0.030481	5.52888	4.60757	0.712446
Vorinostat	MYCN	663	25	0.00558859	0.281225048	0.0885589	1.14141	0.0922058	0.115972
VX-680	MYCN	355	25	0.649953	0.293156819	-0.0351276	2.35922	2.31374	0.934278
OSI-906	MYCN	665	25	0.00418768	0.353332837	-0.0331439	3.44913	1.98539	0.0977126
ZM-447439	MYCN	642	26	0.000107534	0.353637246	0.0599163	2.97674	1.68667	0.0069408
QS11	MYCN	665	25	0.290149	0.362438229	0.0213664	3.96269	3.68273	0.751846
Methotrexate	MYCN	663	25	0.249704	0.425972929	-0.00741431	0.483483	0.65277	0.723843
OSU-03012	MYCN	665	25	0.228711	0.439066194	-0.0383004	3.13261	3.45487	0.706899
BAY 61-3606	MYCN	665	25	0.605774	0.440129015	0.029318	2.65854	2.4318	0.91608
PAC-1	MYCN	665	25	0.30013	0.452918437	-0.00240477	3.46285	3.11705	0.755648
AICAR	MYCN	663	25	0.0386602	0.476345427	-0.0352158	8.21457	8.42227	0.335325
GSK-650394	MYCN	665	25	0.486651	0.481901191	-0.0154645	4.26113	4.22894	0.868459
Mitomycin C	MYCN	665	25	0.335194	0.534143564	0.0123004	-1.10889	-1.02453	0.78655
CEP-701	MYCN	663	25	0.0117301	0.551063949	0.137619	-0.275621	-0.873251	0.18077
Axitinib	MYCN	663	25	0.0628123	0.560340483	0.0337669	3.09824	2.45822	0.421292
PF-562271	MYCN	672	26	0.0266798	0.561221112	0.102367	2.88291	2.70906	0.283028
AZD7762	MYCN	663	25	0.00921422	0.573696235	0.117877	-0.245575	-0.792295	0.1555
PD-0332991	MYCN	633	26	0.508188	0.579861133	0.0239354	2.69056	2.34934	0.878808
JNK Inhibitor VIII	MYCN	661	26	0.028968	0.605817759	-0.00732601	5.99778	5.57067	0.296237
BX-795	MYCN	659	26	0.244597	0.625227397	0.0128616	2.35212	1.60907	0.718255
Imatinib	MYCN	359	25	0.271146	0.626668695	-0.0290436	4.83213	4.70821	0.738206
Camptothecin	MYCN	663	25	0.432739	0.645238689	0.025919	-4.01124	-4.64046	0.842426
Epothilone B	MYCN	665	25	0.368465	0.685054621	0.0202401	-4.7685	-4.44229	0.810118
NSC-87877	MYCN	672	26	0.574995	0.70563189	-0.00738962	6.60198	6.60167	0.903914
LAQ824	MYCN	665	25	0.757507	0.711528008	0.0475988	-2.7742	-2.68253	0.971689
BMS-754807	MYCN	665	25	0.0591397	0.72414544	-0.0214165	1.24843	0.43582	0.40738
Parthenolide	MYCN	353	24	0.200715	0.741282248	0.0376962	4.83948	4.55609	0.669949
PHA-665752	MYCN	358	25	0.0068125	0.747175117	-0.0606826	4.77189	4.76723	0.133038
EHT 1864	MYCN	655	24	0.615758	0.759135673	0.0300669	4.6266	4.54701	0.920258
Thapsigargin	MYCN	665	25	0.00000127	0.776902287	0.199592	-4.03493	-3.99713	0.0002071
GW 441756	MYCN	663	25	0.566113	0.780078665	-0.00722484	4.04568	3.80271	0.903527
Nutlin-3a	MYCN	661	26	0.000000813	0.782352365	0.0914537	4.64933	4.0434	0.0001393
Sunitinib	MYCN	355	25	0.458238	0.788156025	0.00807471	3.11189	3.90644	0.858279
GSK269962A	MYCN	353	24	0.150767	0.792672515	0.0309754	3.44376	3.00397	0.602099
SB590885	MYCN	641	26	0.975958	0.832869196	-0.00945431	5.03294	5.51198	1.01848
Etoposide	MYCN	672	26	0.659007	0.841906379	0.00970601	1.8599	2.10136	0.937665
Lenalidomide	MYCN	663	25	0.459988	0.843482351	0.00221078	5.42334	5.49222	0.858644
LFM-A13	MYCN	665	25	0.145132	0.858300173	0.012893	6.22928	6.12854	0.595136

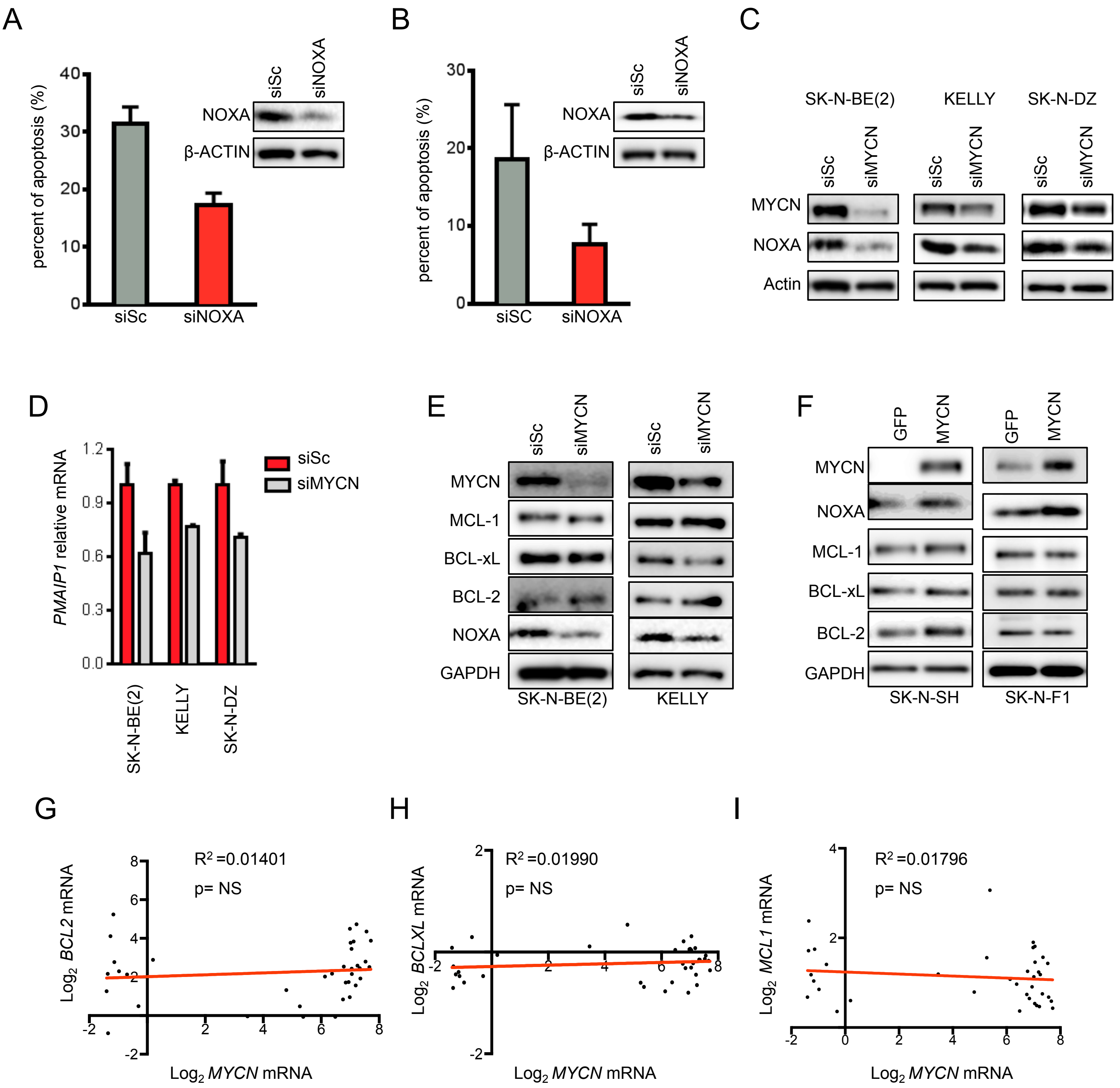

Figure S2 (related to Figure 2). NOXA is involved in *MYCN*-amplified neuroblastoma cell hypersensitivity to ABT-263.

(A, B) (A) SK-N-DZ and (B) SK-N-BE(2) *MYCN*-amplified neuroblastoma cells transfected with short interfering (si)-scrambed (Sc) or siNOXA were subsequently treated for 72 hr with ABT-263 and assayed for apoptosis or (inset) lysed to confirm knockdown. Error bars are +S.E.M.

(C, D) SK-N-BE(2), KELLY and SK-N-DZ cells were transfected with short interfering (si)-scrambed (Sc) or siMYCN and lysed to detect MYCN, NOXA or β-Actin protein (C) or mRNA levels of NOXA relative to β-Actin (D) was determined. Error bars are +S.D. for (D).

(E) SK-N-BE(2) and KELLY cells were transfected with short interfering (si)-scrambed (Sc) or siMYCN and lysed to detect the indicated antibodies.

(F) *MYCN*-wildtype neuroblastoma SK-N-SH and SK-N-F1 cells were engineered to express GFP or MYCN and the lysates were probed with the indicated antibodies.

(G-I) RNA expression levels of BCL-2 family members in Garnett et al (2012). p values were not significant (NS) for the linear regression analysis. Data from Oncomine and is Log_2 median-centered.

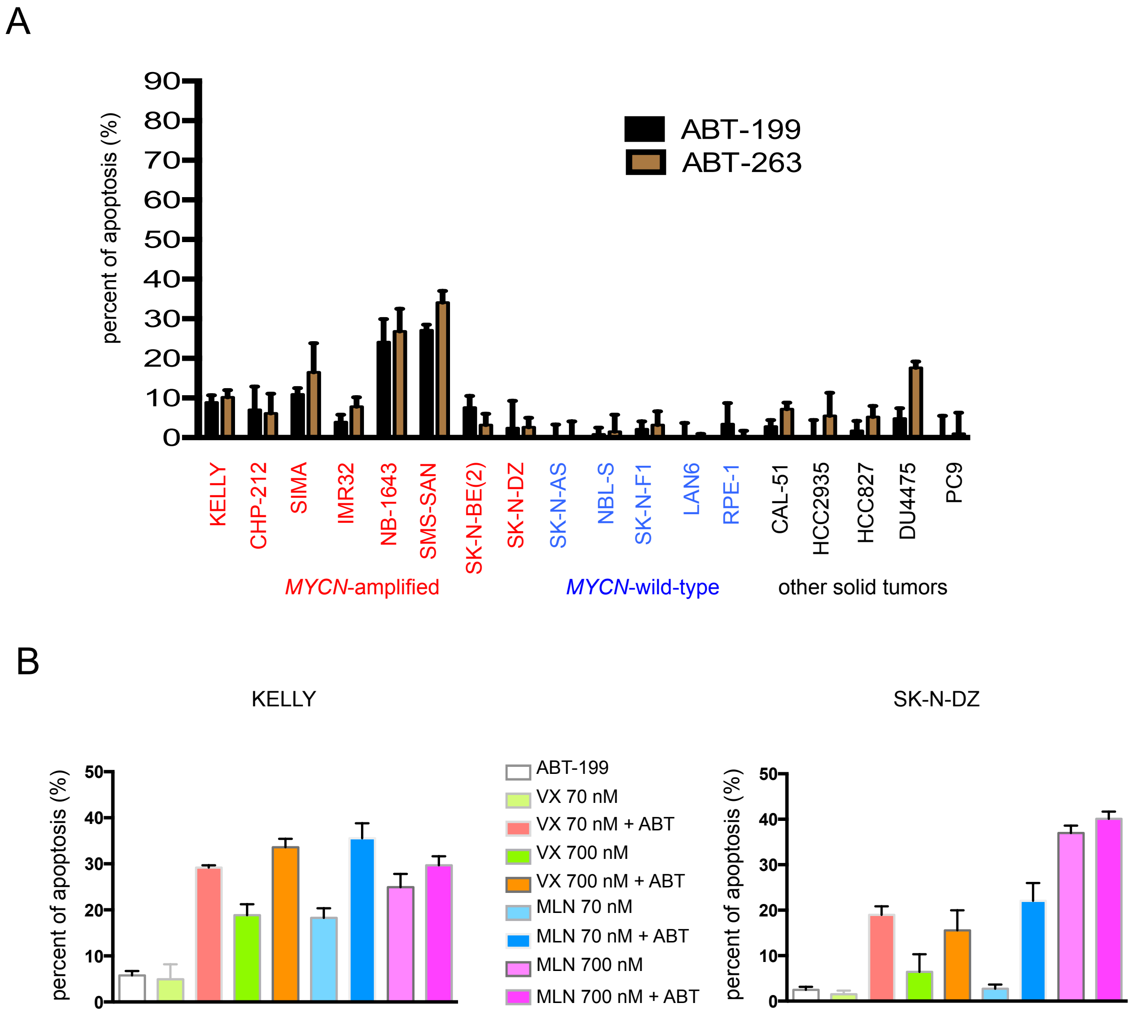

C

KELLY

MLN8237 (nM)

ABT199 (μM)	800	400	200	100	50	25	12.5
10	-0.5	-0.3	-0.2	-0.1	-0.2	-0.3	-0.1
5	1.5	3.2	3.8	13.1	7.4	9.1	13.3
2.5	5.0	9.0	13.3	33.1	19.7	20.7	18.4
1.25	4.6	8.4	11.5	29.2	17.1	15.1	10.1
0.63	5.2	8.3	11.5	25.4	17.3	15.9	3.5
0.32	5.1	8.4	12.0	26.8	17.0	18.2	14.2
0.16	5.0	9.0	12.2	27.6	17.9	20.6	9.3

SK-N-DZ

MLN8237 (nM)

ABT199 (μM)	800	400	200	100	50	25	12.5
10	-1.5	-1.8	-2.9	0.0	4.0	1.6	-1.2
5	5.4	7.3	7.9	5.3	46.6	44.1	12.5
2.5	1.4	4.1	2.4	8.1	34.2	34.6	14.4
1.25	1.6	0.6	1.4	7.3	25.5	20.3	12.2
0.63	1.6	3.0	1.8	4.9	24.0	12.1	7.6
0.32	3.0	7.6	5.5	9.4	23.7	13.1	5.9
0.16	2.5	2.7	3.0	5.1	19.4	0.6	7.0

Figure S3 (related to Figure 3 and 4). Synergy of ABT-199 and Aurora A inhibitors at low concentrations.
(A) The indicated cell lines were treated as in Figure 3B, for a duration of 24 hr and apoptosis was detected by FACS. Error bars are +S.D., n=3.
(B) Apoptosis as determined by FACS following 24 hr of indicated drug treatments in KELLY and SK-N-DZ cells. Error bars are +S.D., n=3.
(C) A dose matrix of MLN8237 and ABT-199 was created in KELLY and SK-N-DZ cells. Viability was assessed after 48 hr. Percent of the excess over the Bliss at each dose of the drug is presented.

Table S2 related to Figure 4. Drugs used in the apoptosis “anchor” screen

Drug	Other Name	Target(s)
Afatinib	BIBW2992	EGFR, HER2
Crizotinib	PF-2341066, Xalkori	MET, ALK
Gefitinib	ZD1839, Iressa	EGFR
Lapatinib	Tykerb, GW572016	ERBB2
AZD6244	Selumetinib, ARRY- 142886	MEK1/2
MK2206		AKT
BYL-719	Alpelisib	PI3K alpha
GDC-0941	Pictilisib	PI3K alpha/beta
PKC412	Midostaurin	PKC, VEGFR, PDGFR, FLT3, KIT
PF299804	Dacomitinib	EGFR, ERBB2
MLN8237	Alisertib	AURKA
VX680	Tozasertib, MK-0457	FLT3, Aurora kinases
AP26113	Brigatinib	ALK, EGFR
WZ4002		EGFR L858R /T790M
Dasatinib	BMS-354825, Sprycel	SRC, ABL, EPHB, SFK
Dabrafenib	GSK2118436	BRAF, CRAF
Imatinib	Gleevac, STI0571, CGP 57148	KIT, PDGFR, ABL
MP-470	Amuvatinib	KIT, PDGFR, FLT3
XL880	Foretinib, GSK1363089	MET, KDR, VEGFR, FLT4,
GSK429286A	KIN001-155	Rho kinase
Vorinostat	Zolinza, SAHA	HDAC inhibitor (Class I, Iia, Iib, IV)
NVP-BGJ398		FGFR
CT99021	CHIR-99021	GSK3
GDC-0449	Vismodegib	Hedgehog pathway

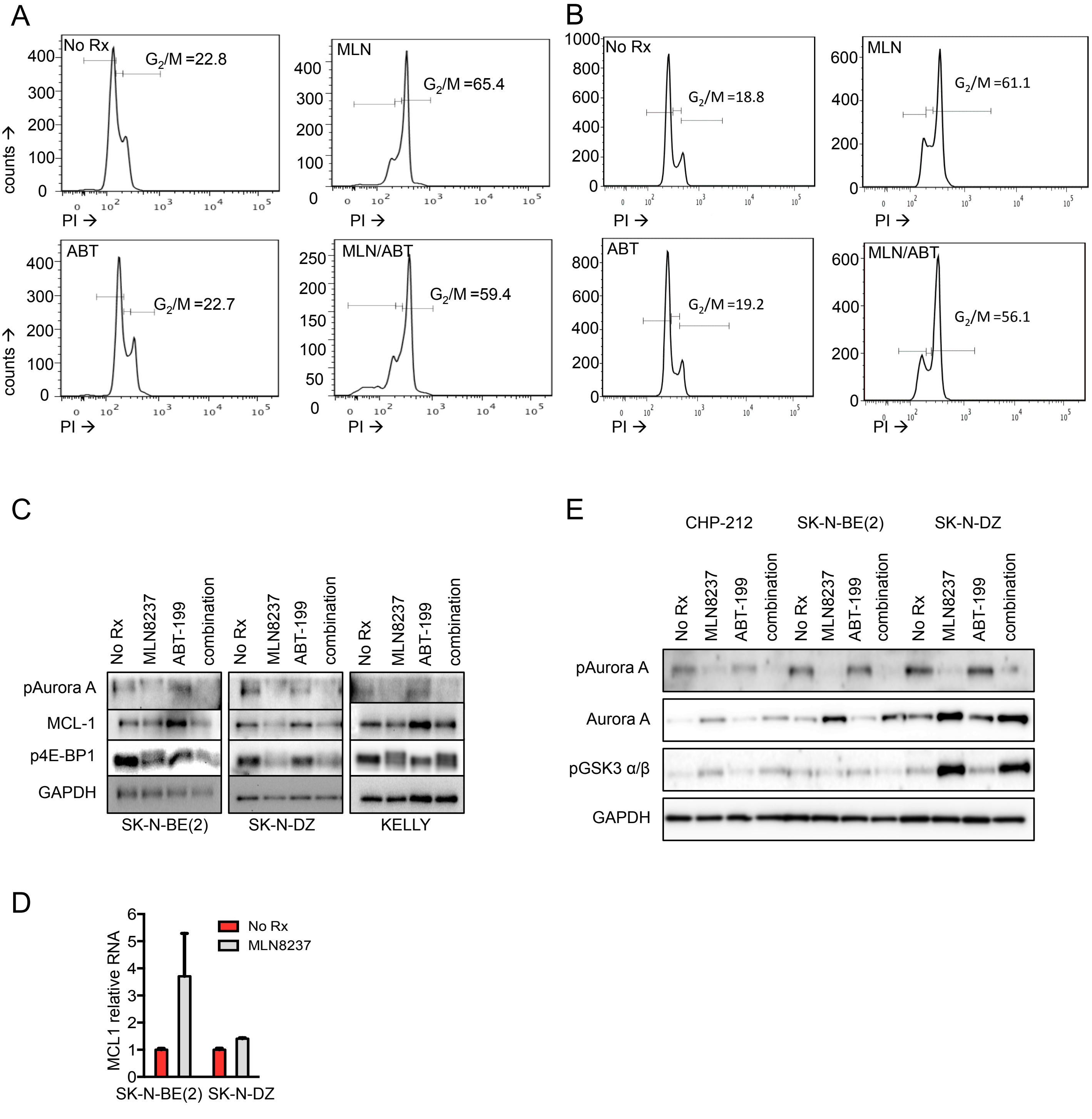

Figure S4 (related to Figure 5). Mitotic Arrest following MLN8237 treatment.
(A, B) Cell cycle profiles of *MYCN*-amplified KELLY (A) and SIMA (B) cells 24 hr following no treatment (No Rx), MLN8237 (MLN) treatment, ABT-199 (ABT) treatment, and combination treatment (MLN/ABT).
(C) Lysates from the indicated *MYCN*-amplified neuroblastoma cell lines were probed with the indicated antibodies following 16 hours of treatment with the indicated drugs.
(**D**) Relative MCL-1 RNA levels following no drug (No Rx) or MLN8237 24 hr after treatment in SK-N-BE(2) and SK-N-DZ. Error bars are +S.D.
(E) Lysates from the indicated *MYCN*-amplified neuroblastoma cell lines were probed with the indicated antibodies following 24 hr of treatment with the indicated drugs. Lysates from **(E)** were overlapping with those in main Figure 5B.

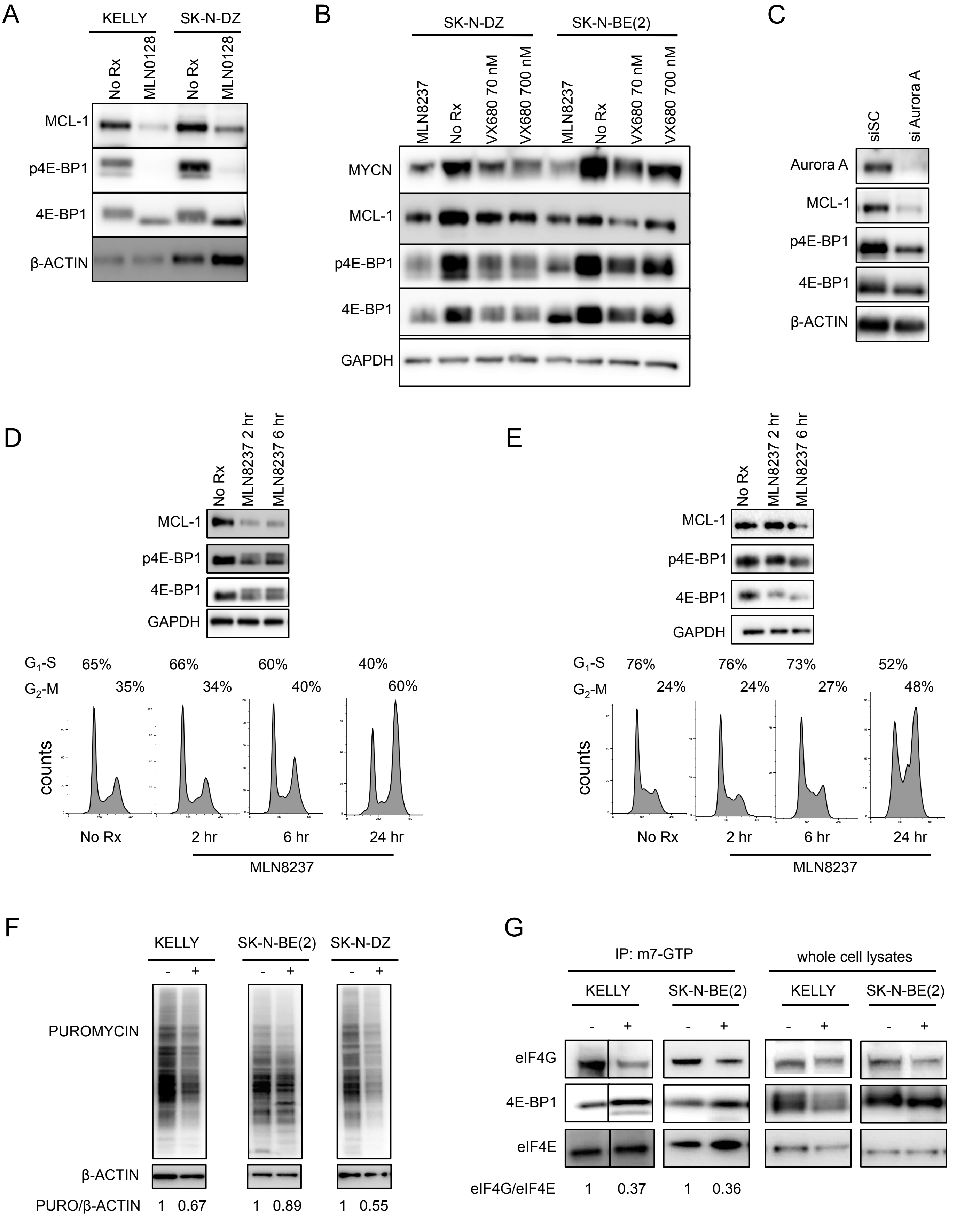

A
KELLY
SK-N-DZ
No Rx
MLN0128
No Rx
MLN0128
MCL-1
p4E-BP1
4E-BP1
β-ACTIN
B
SK-N-DZ
SK-N-BE(2)
MLN8237
No Rx
VX680 70 nM
VX680 700 nM
MLN8237
No Rx
VX680 70 nM
VX680 700 nM
MYCN
MCL-1
p4E-BP1
4E-BP1
GAPDH
C
siSC
si Aurora A
Aurora A
MCL-1
p4E-BP1
4E-BP1
β-ACTIN
D
No Rx
MLN8237 2 hr
MLN8237 6 hr
MCL-1
p4E-BP1
4E-BP1
GAPDH
G1-S 65% 66% 60% 40%
G2-M 35% 34% 40% 60%
counts
No Rx
2 hr
6 hr
24 hr
MLN8237
E
No Rx
MLN8237 2 hr
MLN8237 6 hr
MCL-1
p4E-BP1
4E-BP1
GAPDH
G1-S 76% 76% 73% 52%
G2-M 24% 24% 27% 48%
counts
No Rx
2 hr
6 hr
24 hr
MLN8237
F
KELLY
SK-N-BE(2)
SK-N-DZ
- + - + - +
PUROMYCIN
β-ACTIN
PURO/β-ACTIN 1 0.67 1 0.89 1 0.55
G
IP: m7-GTP
whole cell lysates
KELLY
SK-N-BE(2)
KELLY
SK-N-BE(2)
- + - + - + - +
eIF4G
4E-BP1
eIF4E
eIF4G/eIF4E 1 0.37 1 0.36

Figure S5 (related to Figure 5) MLN8237 downregulates MCL-1 through inhibition of cap-dependent protein translation.
(A) KELLY and SK-N-DZ cells were untreated (No Rx) or treated with100 nM MLN0128 for 6 hr and lysates were probed with the indicated antibodies.
(B) *MYCN*-amplified cell lines, SK-N-DZ and SK-N-BE(2), were treated with the indicated drugs at the indicated concentrations for 24 hr and probed with the indicated antibodies.
(C) SK-N-DZ cells were treated with 50 nM control (siSC) or 50 nM siAurora A siRNA for 24 hr and lysates probed with the indicated antibodies.
(D, E) Top: SK-N-BE(2) (D) and KELLY (E) cells were left untreated (no Rx) or treated with 100 nM MLN8237 for 2 hr or 6 hr, and lysates were probed with the indicated antibodies. Bottom: Cell cycle analysis of SK-N-BE(2) and KELLY cells at the indicated times following treatment with 100 nM MLN8237.
(F) *MYCN*-amplified neuroblastoma cells were left treated with DMSO (-) or 100 nM MLN8237 (+), followed by 1 μM of puromycin exposure and lysates were subjected to Western blot analysis with the indicated antibodies.
(G) Cells were treated with DMSO (-) or 100 nM MLN8237 (+) and lysates were subjected to m^7GTP pull-downs, and analyzed for levels of the indicated proteins. Bands were quantified and the ratio of eIF4G:eIF4E was presented. Immunoprecipiated lysates from KELLY cells were run on the same gel but in discontigious lanes.

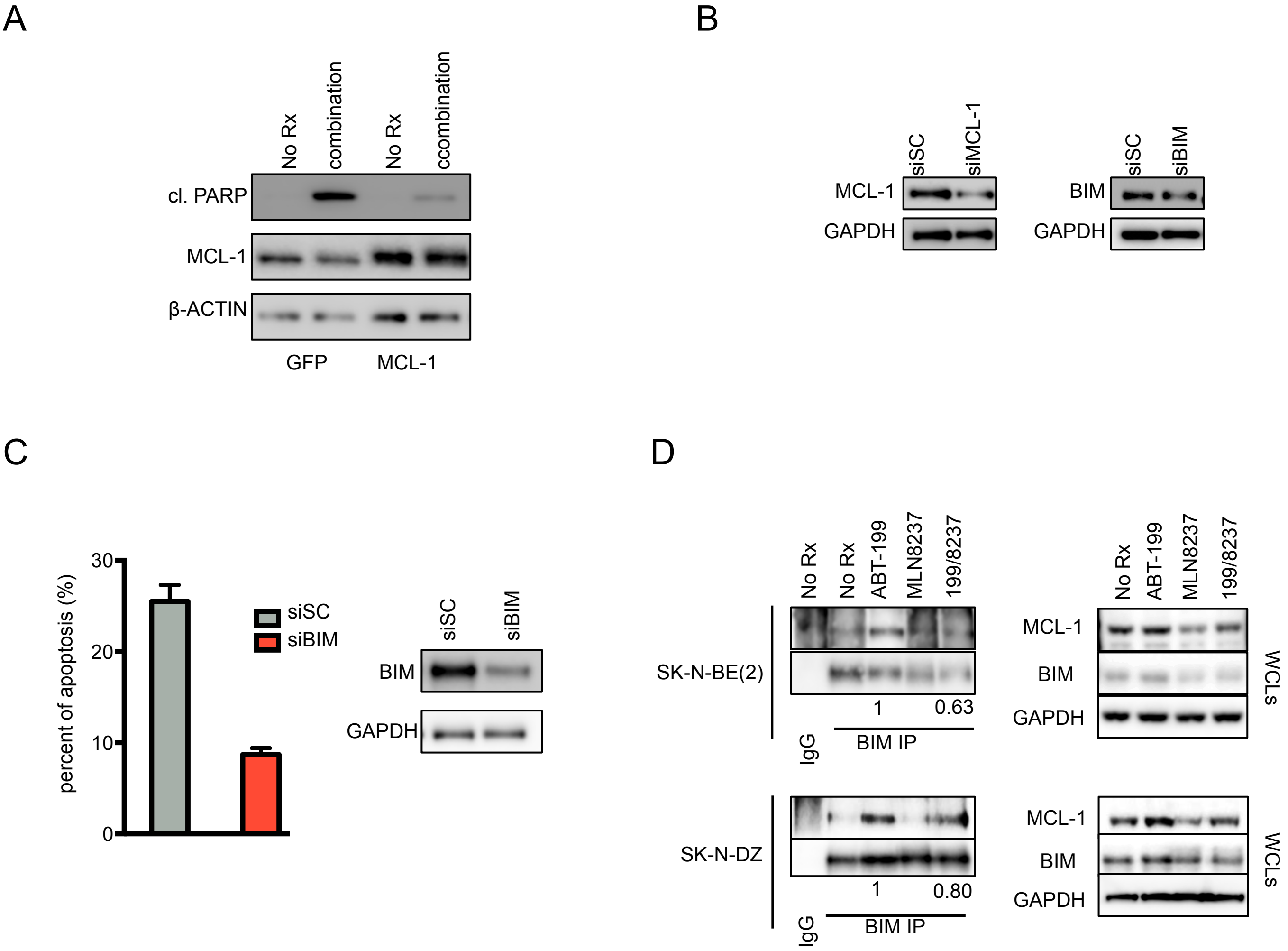
A
No Rx
combination
No Rx
ccombination
cl. PARP
MCL-1
β-ACTIN
GFP
MCL-1
B
siSC
siMCL-1
MCL-1
GAPDH
siSC
siBIM
BIM
GAPDH
C
percent of apoptosis (%)
30
20
10
0
siSC
siBIM
siSC
siBIM
BIM
GAPDH
D
No Rx
No Rx
ABT-199
MLN8237
199/8237
SK-N-BE(2)
1
0.63
IgG
BIM IP
No Rx
ABT-199
MLN8237
199/8237
MCL-1
BIM
GAPDH
WCLs
SK-N-DZ
1
0.80
IgG
BIM IP
MCL-1
BIM
GAPDH
WCLs

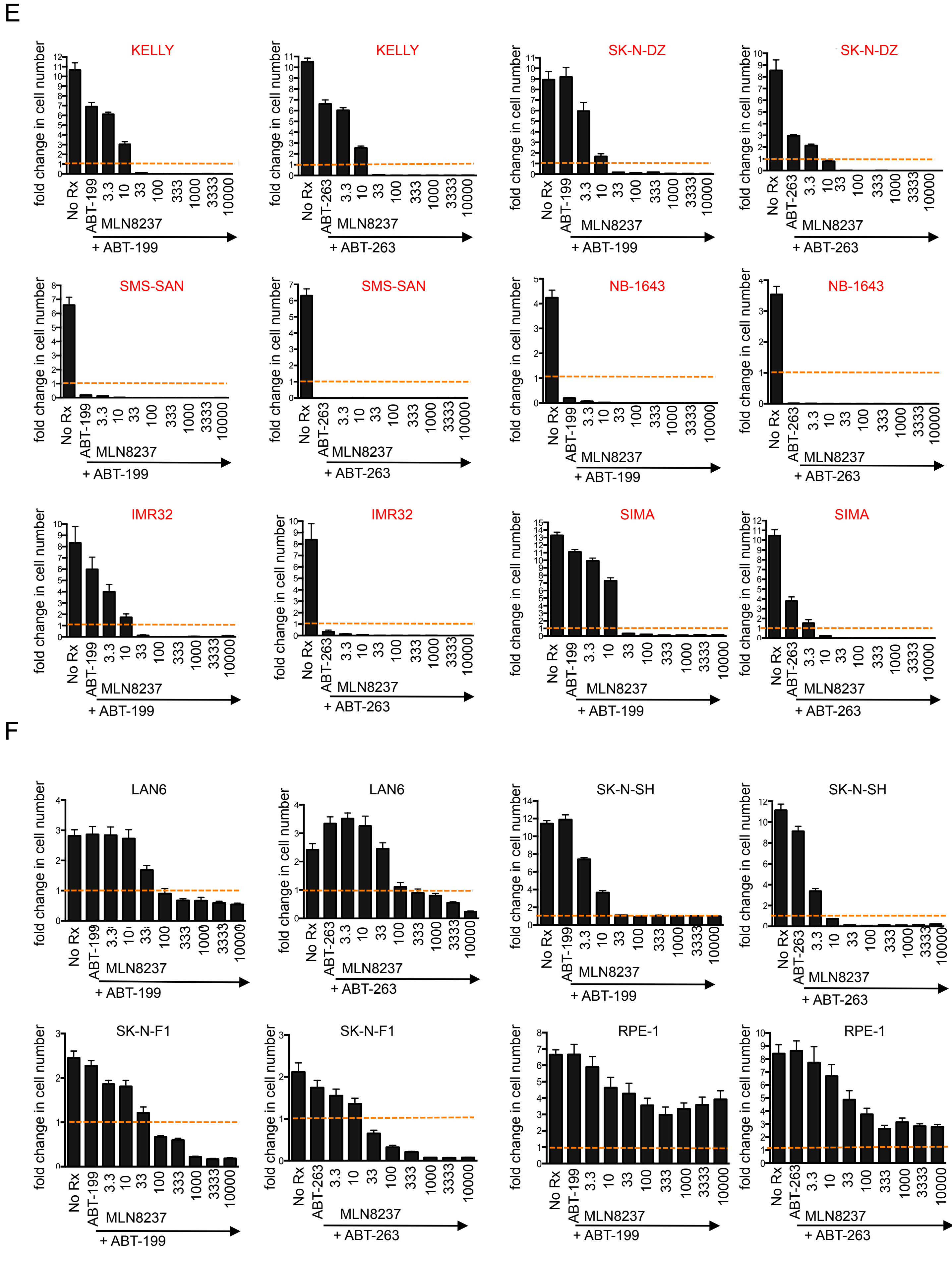
E
KELLY
SK-N-DZ
SMS-SAN
NB-1643
IMR32
SIMA
fold change in cell number
No Rx
ABT-199
ABT-263
3.3
10
33
100
333
1000
3333
10000
MLN8237
+ ABT-199
+ ABT-263
F
LAN6
SK-N-SH
SK-N-F1
RPE-1

Figure S6 (related to Figure 5). MCL-1 is critical in MLN8237/ABT-199 combination-mediated toxicity.
(A) SK-N-DZ cells engineered to overexpress GFP or MYCN were treated for 24 hr with no drug (No Rx) or the combination of 100 nM MLN8237 and 1 μM ABT-199 and lysates were probed for the indicated antibodies. **(B)** SK-N-BE(2) cells treated with control siRNA (siSC) or siMCL-1 (left panel) or siBIM (right panel) were lysed and assayed for knockdown efficiency. Figure is related to main Figure 5C.
(C) SK-N-DZ cells were treated with siSC or siBIM, and cells were treated with the combination and assayed for apoptosis 48 hr later (left). FACS apoptosis is presented as amount of apoptosis minus each condition minus no treatment scrambled control. Error bars are +S.E.M. (n=3). Insets, Western blots of scrambled (Sc) or BIM siRNA-treated cells (right).
(D) Lysates from cells treated for 24 hr were immunoprecipitated with BIM or an IgG control (left) or not immunoprecipitated (Whole Cell Lysates, right) (5% of immunoprecipitated proteins) and probed with the indicated antibodies. Ratio of quantified band intensities of MCL-1 complexed to MCL-1/BIM that was pulled down, for the indicated treatments.
(E,F) *MYCN*-amplified neuroblastoma cell lines (E) or *MYCN*-wild-type neuroblastoma cell lines or the RPE-1 cell line (F) were treated for 6 days with 1 μM ABT-199 or 1 μM ABT-263 in the presence or absence of increasing concentrations of MLN8237 and viability was determined. Error bars are +S.E.M. (n=3-4).

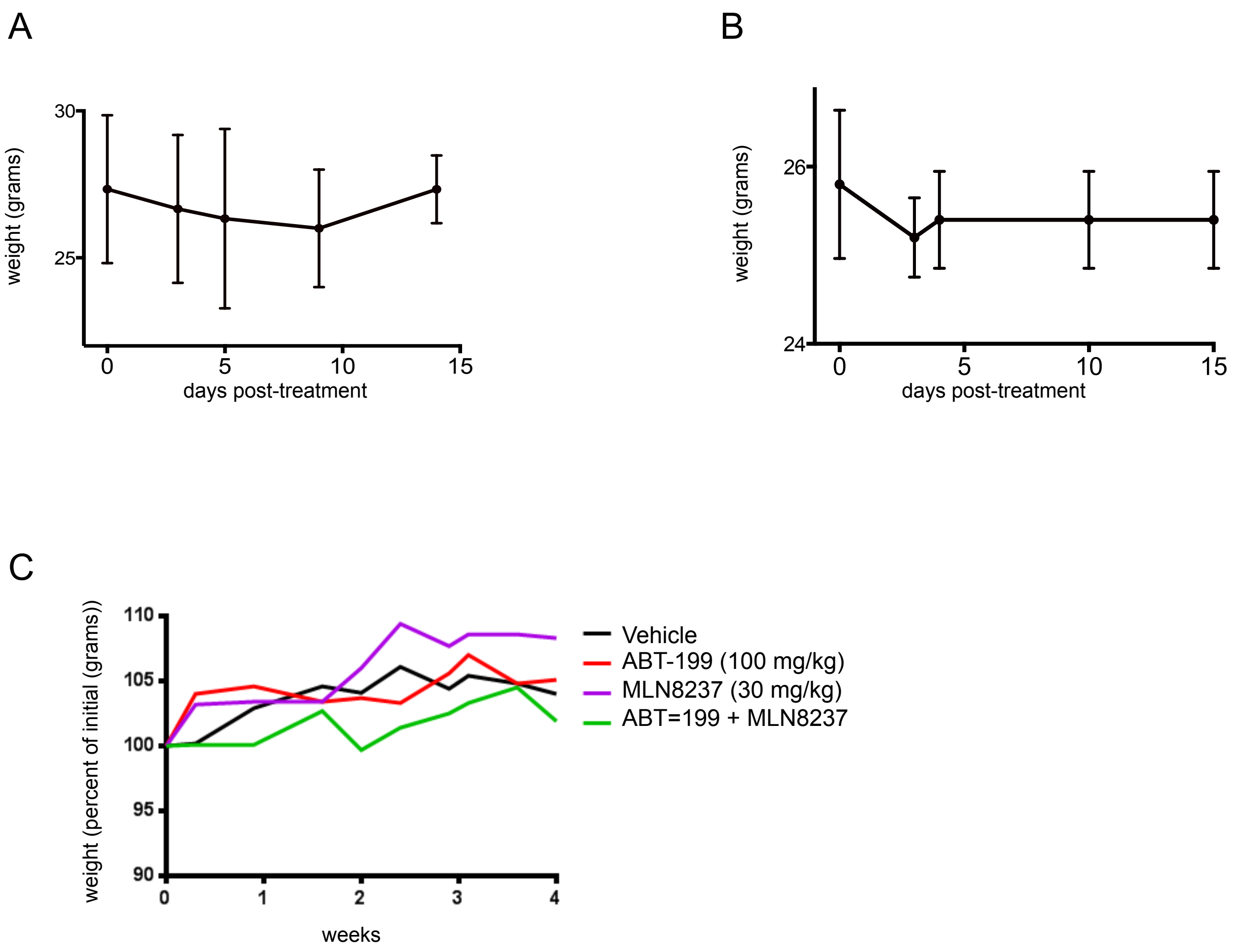

Figure S7 (related to Figure 6 and 7). No weight loss in mouse models treated with the combination.

(A,B) Weights of human xenograft-bearing mice (A, Kelly) and (B, SK-N-SH) from the in vivo experiments. Error Bars are -/+ S.D.

(C) Weights of PDX-bearing mice from the in vivo experiment.

Supplemental Experimental Procedures

Cell Lines

The cell lines in this study were from the Center for Molecular Therapeutics at Massachusetts General Hospital, The Children's Hospital of Philadelphia and Massachusetts General Hospital. KELLY, SIMA, IMR32, NB-1643, SMS-SAN, RPE-1, NBL-S, LAN6, SK-N-F1, SK-N-BE(2), SK-N-DZ, CAL-51, HCC2935, DU4475, and PC9 cells were cultured in RPMI (the Lonza Group) with 10% FBS (Seradigm) in the presence of 1µg/ml penicillin and streptomycin. HCC827 cells were cultured in RPMI with 5% FBS in the presence of antibiotics. CHP-212, SK-N-SH, and SK-N-AS cells were grown in DMEM/F12 (HyClone Laboratories, Inc.) with 10% FBS in the presence of 1µg/ml penicillin and streptomycin. 293T cells were grown in in DMEM with 10% FBS in the presence of 1µg/ml penicillin and streptomycin.

The Genomics of Drug Sensitivity in Cancer

The website for publically accessible drug-sensitivity data (www.cancerRxgene.org) contains the complete drug sensitivity profiles of the 130 drugs tested as well as the efficacy of different drugs compared between 26 *MYCN*-amplified cancers (including 20 neuroblastomas) and the remainder of the cancer cell lines (*MYCN*-wild-type). Table S1 is derived from this dataset and has been modified from the downloadable data.

Antibodies and Reagents

The antibodies used for Western blot analyses were n-Myc (MYCN) (cat #9405), β-Actin (cat #4967), phospho-Erk (cat #9101), phospho-GSK-3α/β(cat #9331), eIF4G (cat #2469), eIF4E (cat #2067), phospho-4E-BP1 (cat #2855), total 4E-BP1 (cat #9452) cleaved PARP (cat #5625), phospho-Aurora A/B/C (cat #13464), total aurora A (cat #12100), and Bim (cat #2933); all these antibodies were from Cell Signaling Technology (Beverly, MA). n-Myc (sc-53993), MCL-1 (sc-819) and normal Rabbit IgG from Santa Cruz Biotechnology (Dallas, TX) were used. The NOXA antibody (product # MA1-41000) was from Pierce-Thermo Scientific (Waltham, MA). GAPDH or β-Actin antibody were used as loading controls. The Protein A beads were from GE Healthcare (Little Chalfont, United Kingdom). The reagents used for apoptosis assay were Annexin V-Cy5 reagent (cat #1013-200) from BioVision (Milpitas, CA), propidium iodide (cat #51-66211E) from BD Biosciences (San Jose, CA), and Guava Nexin Reagent (part #4700-1140) from Millipore. Reagents used for siRNA experiments were Opti-MEM (cat #31985-070) from Life Technologies/Thermo Fisher (Waltham, MA) and HiPerfect Transfection Reagent (cat # 301705) from Qiagen (Valencia, CA). MLN8237, MLN0128 and ABT-199 were from Abmole (Houston, TX).

Western Blotting and Immunoprecipitation

Cell lines, tumors from traditional human xenografts, and tumors from patient-derived xenografts were prepared and lysed in lysis buffer (20mM Tris, 150mM NaCl, 1% NP-40, 1 mM EDTA, 1mM EGTA, 10% glycerol, and protease and phosphatase inhibitors), incubated on ice for 10 min and centrifuged at 10,500 rpm for 10 min at 4 °C. Tumor lysates were homogenized with Tissuemiser (Fisher Scientific) in the lysis buffer described previously, incubated for 20 min on ice, and centrifuged at 10,500 rpm for 10 min at 4 °C. Equal amounts of the detergent-soluble lysates were resolved using the NuPAGE® Novex® Midi Gel system on 4% to 12% Bis-Tris Gels (Invitrogen), transferred to PVDF membranes (PerkinElmer) in between 6 pieces of Whatman paper (Fisher Scientific) set in transfer buffer from Biorad with 20% methanol, and following transfer and blocking in 5% non-fat milk in PBS, probed overnight with the antibodies listed above. Representative blots from several experiments are shown in the Figures. Chemiluminescence was detected with the Syngene G:Box camera (Synoptics). For immunoprecipitation, cells were lysed in the same buffer; 250ug to 500ug were incubated with either BIM antibody (500 ng), MCL-1 antibody (500 ng), or rabbit IgG (500 ng). As Cell Signaling antibody concentrations change from lot to lot, precise concentrations were verified by the company. Following the addition of 25uL of 1:1 PBS: pre-washed Protein A beads to the antibody/lysate mix, samples were incubated with rotating motion overnight. Equal amounts of extracts (10% of immunoprecipitated protein) were prepared in parallel. For the immunoprecipitations, all antibodies from each panel were run on the same gel for precise comparisons.

Apoptosis
Cells were seeded in triplicate at roughly 30-40% confluency and treated for the indicated time with the indicated treatments, with no-treatment controls in parallel. Apoptosis experiments staining with propidium iodide and Cy5-Annexin V, including data from Figures 3B and 5A and Figure S3B were analyzed on a BD LSR III (Becton Dickenson), with the exception of the NBL-S cells from Figure 3B (see below). Apoptosis was determined 72 hr after treatments in Figure 3B and 24 hr after treatments in Figure S3B-S4B, and 48 hr in Figure 5A. With the exception of the KELLY cells, which were quantified 24 hr later for the due to the quick death kinetics of the cells. For the apoptosis experiments from data in Figures 2A, 2B, 2H, 5C and Figures S3A and S7C and the NBL-S cells from Figure 3B, cells were stained with Guava Nexin (Millipore) according to the manufacturer's protocol, and subsequently analyzed on a Guava easyCyte FACS machine (Millipore). For all apoptosis experiments staining with propidium iodide and Annexin, the number of cells in quadrants II and IV (Annexin V positive) were counted as apoptotic.

Cell Cycle
Cells were seeded in triplicate at roughly 30-40% confluency and treated for 24 hr, with no-treatment controls in parallel. The cells were trypsinized, washed and re-suspended with 0.1%Triton in PBS. The cells were then stained with Propidium Iodide, incubated for 20 min at 37 degrees °C and immediately analyzed on a BD LSR III for cell cycle profiles (Becton Dickenson, Figure S4A-S4B) or a Guava easyCyte FACS Machine (Figure S5D-S5E).

Cell Viability
For the long-term (six-day) ABT-199/MLN8237 and ABT-263/MLN8237 Cell Titer-Glo experiments in Figures S6E-S6F, sparsely seeded cells in two parallel 96-well flat bottom black plates were either 1) treated with 50 μL of CellTiter-Glo (Promega) the next day and immediately frozen ("day 0 plate"), or 2) treated the next day with drug. Following six days of continuous drug treatment at 37 degrees and 5% atmospheric CO_2, 50 μL of CellTiter-Glo was added to cells and immediately frozen. Cells were then thawed on a rocker with motion (Day 0 plate and treatment plate), and upon thawing, were read on a Centro LB 960 microplate luminometer (Berthold Technologies) according to the Promega protocol. The quantification of cells from the day 0 plate was used to determine the total cell growth number over the experiment. Cells were seeded in at least triplicate. For the 72 hr viability assays including data from Figures 2G, 2I, and 4C, sparsely seeded cells were plated in a flat-bottom black 96-well plate and the next day the indicated drug(s) at the indicated concentration(s) were added. 72 hr later, CellTiter-Glo (Promega) was added per well and read on the Centro LB960 microplate luminometer according to the Promega protocol. For the five-day crystal violet experiments, 100,000 cells were seeded (300,000 for the SMS-SAN and NB1643 cells) and the next day cells were treated with nothing, 10 nM MLN8237, 1 μM ABT-199 or the combination and five days later viable cells were stained with 0.1% crystal violet (Sigma-Aldrich) as shown in Figures 5F-5G.

RNA extraction and quantitative (Q) RT-PCR
RNA was isolated from cultured cells grown at sub-confluency using the Zymo Quick-RNA MiniPrep kit (Zymo Research), and RNA was reverse-transcribed to from cDNA molecules using cDNA synthesis kit superscript II (Invitrogen, Carlsbad, CA) on a 7500 Fast Real-Time PCR System (Life Technologies). The number of NOXA, MCL1 and β-ACTIN molecules was monitored in real time on a GENEAMP PCR System 9700 (Life Technologies) by measuring the fluorescence increases of SYBR Green (Roche). The primers for *PMAIP1* Forward were: 5′-GCTGGAAGTCGAGTGTGCTA-3′ and *PMAIP1* Reverse 5′-CCTGAGCAGAAGAGTTTGGA-3; *MCL1* Forward (5'-GGGCAGGATTGTGACTCTCATT-3') and *MCL1* Reverse (5'-GATGCAGCTTTCTTGGTTTATGG-3'); *ACTB* Forward (5'-GGCATGGGTCAGAAGGATT-3') and *ACTB* Reverse (5'-AGGATGCCTCTCTTGCTCTG-3'). To determine relative abundance of *PMAIP1* and *MCL1* in relation to *ACTB* , the Delta-Delta CT (cycle threshold) method was utilized.

siRNA Experiments
For the short-interfering (si)RNA experiments, siRNA designed against *MCL-1, MYCN, BIM,* and *AuroraA* and scrambled control oligos were used at a concentration of 50nM and transfected with Hiperfect reagent (Qiagen). In general, cells were plated in antibiotic-free media to achieve next day confluency of roughly 60%. Next, 75 μL of Hiperfect was added to 750 μL of OPTI-MEM in a 1.5 mL eppendorf tube. In parallel, scramble siRNA was added to 750 μL of Opti-MEM in a second, separate 1.5 mL eppendorf tube. Following gently mixing and incubation for ten minutes, the tubes were combined, and again, mixed gently. Following an additional ten min incubation time, the Hiperfect-siRNA mix was added to the appropriate cells. 24 hr later, the cells were re-seeded in 6-well plates, and the next morning were either (1) treated with the appropriate drug for eventual apoptosis determination, (2) lysed for Western blot analysis, or (3) collected for RNA isolation. The siRNA against *MCL-1*, *NOXA* and *Aurora A* were from Dharmacon, and the *BIM*, *MYCN* and scramble control siRNA were from Qiagen. The knockdowns for the scrambled, NOXA and MYCN, for example presented in Figure S2A-S2E, were performed within the same experiments. For the SK-N-BE(2) and SK-N-DZ cells, presented in Figure 5C and Figure S6B-S6C, the knockdowns for the scrambled, MCL-1 and BIM were performed within the same experiments. Apoptosis was measured 48 hours following drug additions in Figure 5C.

shRNA Experiments
For the short-hairpin (sh)RNA experiments, shRNA against NOXA (Hata et al., 2014) and MYCN (Sigma-Aldrich), MISSION shRNA (clone ID:NM_005378.3-230s1c1) were utilized. shRNA designed against a scramble sequence (MISSION pLKO.1-shRNA control plasmid DNA) served as the control. The shRNAs each have a pLKO.1 puromycin-resistant vector backbone and served as the basis for cell selection in puromycin following infection. Cells were transduced with plasmid-containing viral particles and viral particles were generated in 293T cells and collected over 48 hr.

MYCN and MCL-1 Overexpression
The GFP-IRES-MCL-1 pLENTI plasmid and pLENTI-GFP control were previously described (Faber et al., 2014). pMXs-hu-N-Myc (Nakagawa et al., 2010) was a gift from Shinya Yamanaka (Addgene plasmid #50772), and cloned into the pLENTI backbone to form pLENTI-MYCN.

Cap-dependent translation assay
Cells were lysed in the same buffer as used for Western blotting experiments. 30 μL of m7-GTP beads (Jena Bioscence, Germany) were added to an equal amount of cellular lysates. Following overnight incubation at 4 degrees Celsius, the IP complexes were washed three times in lysis buffer, boiled, and ran on a 4-12% BIS-TRIS gel (Invitrogen, Carlsbad, CA).

Global Protein Synthesis assay
Cells were pre-incubated with the indicated drugs, followed by the addition of 1μM puromycin for 30 min. Cells were lysed and subjected to western blot analysis with an anti-puromycin antibody, stripped for the loading control (β-ACTIN).

Combination synergy test
6000 cells /well were seeded in 96-well plates. A 8x8 dose matrix of either MLN8237 or ABT-199 was performed and cell viability was measured at the end of 48 or 72 hr using CellTiter Glo (Promega) according to Promega protocol. Excess over the Bliss values were calculated as a measure of synergy and are displayed in Figure S3C .

Protein Quantification
Bands, where indicated, were quantified on a Syngene using the GeneTools software program. Data is presented as the indicated ratios, with the control (No Rx) or the ABT-199 condition normalized to a value of "1".

Supplemental Reference

Nakagawa, M., Takizawa, N., Narita, M., Ichisaka, T., and Yamanaka, S. (2010). Promotion of direct reprogramming by transformation-deficient Myc. Proceedings of the National Academy of Sciences of the United States of America *107*, 14152-14157.
