# Supplementary material for: Exploitation of the Apoptosis-Primed State of MYCN-Amplified Neuroblastoma to Develop a Potent and Specific Targeted Therapy Combination
Source: Cancer Cell. 2016 Feb 8;29(2):159–72. doi: 10.1016/j.ccell.2016.01.002 (PMC4749542; doi:10.1016/j.ccell.2016.01.002)
Supplement: Document S2. Article plus Supplemental Information [file mmc2.pdf]

Cancer Cell

Article

Exploitation of the Apoptosis-Primed State of *MYCN*-Amplified Neuroblastoma to Develop a Potent and Specific Targeted Therapy Combination

Graphical Abstract

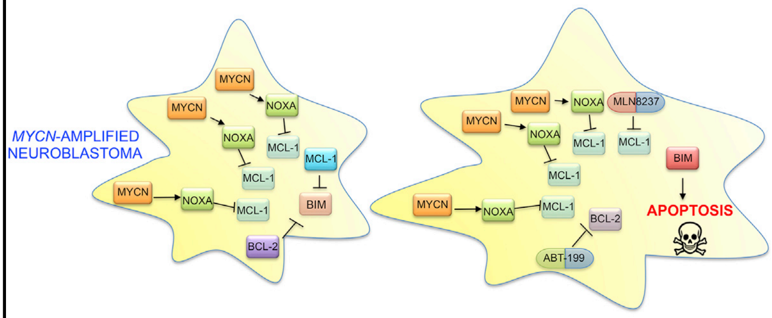

Authors

Jungoh Ham, Carlotta Costa, Renata Sano, ..., Jeffrey A. Engelman, Yael P. Mossé, Anthony C. Faber

Correspondence

acfaber@vcu.edu

In Brief

Ham et al. show that *MYCN*-amplified neuroblastomas are sensitive to treatment with the BCL-2 inhibitor ABT-199 due to MYCN-driven increase of NOXA. Combination treatment with the Aurora Kinase A inhibitor MLN8237 and ABT-199 is synergistic in xenograft models of this tumor type, in part via reducing MCL-1.

Highlights

- Amplified *MYCN* is synthetic lethal with the BCL-2 inhibitor ABT-199 in neuroblastoma
- MYCN upregulates the MCL-1 inhibitor, NOXA
- *MYCN*-amplified neuroblastomas are further sensitized to ABT-199 with MLN8237
- ABT-199 with MLN8237 induce tumor regressions in *MYCN*-amplified neuroblastoma mice

Ham et al., 2016, Cancer Cell *29*, 159–172
February 8, 2016 ©2016 The Authors
http://dx.doi.org/10.1016/j.ccell.2016.01.002

Cancer Cell
Article

Exploitation of the Apoptosis-Primed State of *MYCN*-Amplified Neuroblastoma to Develop a Potent and Specific Targeted Therapy Combination

Jungoh Ham,[1,11] Carlotta Costa,[4,5,11] Renata Sano,[2] Timothy L. Lochmann,[8] Erin M. Sennott,[4,5] Neha U. Patel,[1] Anahita Dastur,[4,5] Maria Gomez-Caraballo,[4,5] Kateryna Krytska,[2] Aaron N. Hata,[4,5] Konstantinos V. Floros,[1] Mark T. Hughes,[1] Charles T. Jakubik,[4,5] Daniel A.R. Heisey,[1] Justin T. Ferrell,[1] Molly L. Bristol,[1] Ryan J. March,[4,5] Craig Yates,[1] Mark A. Hicks,[1] Wataru Nakajima,[9] Madhu Gowda,[10] Brad E. Windle,[1] Mikhail G. Dozmorov,[6] Mathew J. Garnett,[7] Ultan McDermott,[7] Hisashi Harada,[1] Shirley M. Taylor,[8] Iain M. Morgan,[1] Cyril H. Benes,[4,5] Jeffrey A. Engelman,[4,5] Yael P. Mossé,[2,3] and Anthony C. Faber[1,*]

[1]Philips Institute for Oral Health Research, VCU School of Dentistry and Massey Cancer Center, Virginia Commonwealth University, Perkinson Building, Richmond, VA 23298, USA
[2]Division of Oncology and Center for Childhood Cancer Research, The Children's Hospital of Philadelphia, Philadelphia, PA 19104, USA
[3]Department of Pediatrics, University of Pennsylvania Perelman School of Medicine, Philadelphia, PA 19104, USA
[4]Massachusetts General Hospital Cancer Center, Boston, MA 02129, USA
[5]Department of Medicine, Harvard Medical School, Boston, MA 02115, USA
[6]Department of Biostatistics, Virginia Commonwealth University, Richmond, VA 23298, USA
[7]Cancer Genome Project, The Wellcome Trust Sanger Institute, Hinxton CB10 1SA, UK
[8]Department of Microbiology and Immunology, Massey Cancer Center, Richmond, VA 23298, USA
[9]Department of Molecular Oncology, Institute for Advanced Medical Sciences, Nippon Medical School, Kawasaki 211-8533, Japan
[10]Department of Pediatrics, Children's Hospital of Richmond, VCU, Richmond, VA 23298, USA
[11]Co-first author
*Correspondence: acfaber@vcu.edu
http://dx.doi.org/10.1016/j.ccell.2016.01.002
This is an open access article under the CC BY license (http://creativecommons.org/licenses/by/4.0/).

SUMMARY

Fewer than half of children with high-risk neuroblastoma survive. Many of these tumors harbor high-level amplification of *MYCN*, which correlates with poor disease outcome. Using data from our large drug screen we predicted, and subsequently demonstrated, that *MYCN*-amplified neuroblastomas are sensitive to the BCL-2 inhibitor ABT-199. This sensitivity occurs in part through low anti-apoptotic BCL-xL expression, high pro-apoptotic NOXA expression, and paradoxical, MYCN-driven upregulation of NOXA. Screening for enhancers of ABT-199 sensitivity in *MYCN*-amplified neuroblastomas, we demonstrate that the Aurora Kinase A inhibitor MLN8237 combines with ABT-199 to induce widespread apoptosis. In diverse models of *MYCN*-amplified neuroblastoma, including a patient-derived xenograft model, this combination uniformly induced tumor shrinkage, and in multiple instances led to complete tumor regression.

INTRODUCTION

There are now a number of successful targeted therapies that treat genetically distinct cancers (reviewed in Huang et al., 2014). Unfortunately, many cancer subtypes are not yet amenable to targeted therapy treatment, including cancers with well-defined driver oncogenes. For example, *KRAS* mutations are found at high rates in lung, colorectal, and pancreatic cancer, and inhibiting KRAS in these cancers causes tumor growth inhibition (Sunaga et al., 2011; Zorde Khvalevsky et al., 2013). However, KRAS, like most non-kinase driving oncogenes, is not currently pharmacologically targetable. Alternative means

Significance

Targeted therapies are now being developed for a subset of neuroblastomas with ALK mutations. However, MYCN pathway inhibitors have proved difficult to develop. We demonstrate that the presence of *MYCN* amplification in neuroblastoma exhibits synthetic lethality when treated with the BCL-2 targeting agent ABT-199, and that these tumors are further sensitized by the addition of the Aurora A inhibitor, MLN8237, in cell culture models and diverse mouse models. In contrast, *MYCN*-WT neuroblastoma cell culture models and human xenografts proved insensitive to this combination. Therefore, exploiting the paradoxical apoptosis-promoting function of *MYCN* amplification in neuroblastoma could be an effective strategy for the development of better therapies. ABT-199/MLN8237 combination therapy is differentially effective in the high-risk, *MYCN*-amplified subset of neuroblastoma.

of blocking KRAS, such as synthetic lethal approaches or combined inhibition of important downstream pathways, are being pursued to treat these cancers (reviewed in McCormick, 2015).

Neuroblastoma is a neural-crest-derived cancer and is the leading cause of cancer-related deaths in children aged 1–4 years (Gao et al., 1997). High-level amplifications of the *MYCN* oncogene is observed in about 20% of cases, and has long been associated with high-risk disease and poor outcome in neuroblastoma (Huang and Weiss, 2013). *MYCN* encodes an E-BOX-binding, basic-helix-loop-helix-leucine zipper (bHLH-LZ) transcription factor that is enriched in the nervous system. In these cancers, *MYCN* is a bona fide oncogenic driver (Burkhart et al., 2003; Weiss et al., 1997) and, similar to KRAS in KRAS-driven tumors, preclinical inhibition of MYCN protein leads to tumor growth inhibition in neuroblastoma mouse models (Brockmann et al., 2013; Burkhart et al., 2003; Delehouze et al., 2014; Faisal et al., 2011; Gustafson et al., 2014; Puissant et al., 2013). However, MYCN is not amenable to direct pharmacologic inhibition, and preclinical efficacy of indirectly targeting MYCN has largely been modest (Brockmann et al., 2013; Chipumuro et al., 2014; Gustafson et al., 2014; Puissant et al., 2013). As such, augmenting treatment efficacy for this high-risk group will likely require the development of additional rational therapies based on targetable pathways specifically activated in neuroblastomas with *MYCN* amplification. To this point, while MYCN has the pro-growth and pro-survival characteristics of a classical oncogene, MYCN also has the ability to promote apoptosis (Chen et al., 2010; Fulda et al., 1999; Petroni et al., 2011; Veschi et al., 2012).

The Genomics of Drug Sensitivity in Cancer (GDSC) is a comprehensive drug susceptibility discovery program that we have developed to help identify therapeutic strategies for genetically defined subsets of cancer (Garnett et al., 2012; Yang et al., 2013). Previous studies building on the GDSC findings have revealed sometimes unsuspected susceptibilities, such as the sensitivity of Ewing sarcomas driven by a *EWS-FLI1* translocation to PARP inhibitors (Garnett et al., 2012), the response of *KRAS* and *BRAF* mutant colorectal cancers to simultaneous MCL-1/BCL-xL/BCL-2 inhibition (Faber et al., 2014), and the response of *TP53/RB*-deleted small-cell lung cancers to the BH3 mimetic ABT-263 upon TORC1/2 inhibitor addition (Faber et al., 2015). Here, we investigate potential therapeutic opportunities for *MYCN*-amplified neuroblastoma.

RESULTS

MYCN-Amplified Neuroblastoma Cells Are Highly Sensitive to ABT-263, due to High NOXA

Analysis of drug-sensitivity data of ~500 solid tumor cancer cell lines from a high-throughput drug screen (Garnett et al., 2012) indicated that among all solid tumor types, neuroblastoma cell lines were exquisitely sensitive to the in-clinic BCL-2/BCL-2/xL inhibitor ABT-263 (navitoclax) (Figure 1A). Intriguingly, this sensitivity was specific only to neuroblastoma cells with *MYCN* amplification (Figure 1B). In fact, among 130 experimental and clinical drugs, *MYCN*-amplified cell lines (20/26 of which were neuroblastoma cell lines) showed the most significant shift in half maximal inhibitory concentration (IC_{50}) toward sensitivity with ABT-263 than any other drug compared with *MYCN*-WT cell lines (Table S1, top 39 by IC_{50} shift listed). To explore the underlying etiology for this exquisite sensitivity of *MYCN*-amplified cell lines, we examined expression levels of BCL-2 family members that are known to modulate the sensitivity to ABT-263 by interrogating a database of 20 *MYCN*-amplified and 81 *MYCN*-WT neuroblastoma primary tumors (Wang et al., 2006). This analysis uncovered the MCL-1 inhibitor NOXA, encoded by *PMAIP1*, to be significantly higher in *MYCN*-amplified neuroblastomas (Figures 1C, 1D, and S1A–S1C). The paradoxical nature of *PMAIP1* upregulation in *MYCN*-amplified neuroblastoma is underscored by the fact that *PMAIP1* expression was sharply decreased in stage 4 *MYCN*-WT neuroblastomas compared with stage 1–3 *MYCN*-WT neuroblastomas (Figure 1E), consistent with the widely noted suppression of pro-apoptotic proteins as cancers progress (reviewed in Hata et al., 2015). High expression of *PMAIP1* in the subset of *MYCN*-amplified neuroblastomas was confirmed in a second dataset of neuroblastomas (Janoueix-Lerosey et al., 2008), again without differential expression of other key BCL-2 family members (Figure S1D). In addition, gene expression datasets from medulloblastoma tumors (Kool et al., 2008; Northcott et al., 2012; Robinson et al., 2012), a pediatric cancer in which *MYCN* is often amplified (Ryan et al., 2012), indicated that *PMAIP1* mRNA expression positively correlated with *MYCN* mRNA expression (Figures 1F, S1E, and S1F). The positive relationship between *PMAIP1* mRNA expression and *MYCN* mRNA expression was also observed in a large collection of neuroblastoma cell lines (Garnett et al., 2012) (Figure S1G) and confirmed in our neuroblastoma cell line panel (Figure S1H). Altogether, these data indicate high MYCN expression is associated with high NOXA expression.

High levels of NOXA expression can confer sensitivity to BCL-2/BCL-xL inhibitors (Lucas et al., 2012; Nakajima et al., 2014; Nalluri et al., 2015; Wang et al., 2014), and artificial expression of NOXA is sufficient to sensitize cancer cells to BCL-2/BCL-xL inhibitors (Nakajima et al., 2014), suggesting that increased NOXA expression is a contributing factor to ABT-263 sensitivity observed in *MYCN*-amplified neuroblastoma cells. We therefore reduced NOXA expression by stably expressing short hairpins (sh) against NOXA or transfection of siRNA directed against NOXA, with both experimental methods resulting in reduced NOXA expression and protection of *MYCN*-amplified neuroblastoma cell lines from ABT-263-mediated apoptosis compared with controls (Figures 2A, 2B, S2A, and S2B). These data confirm that reduction of NOXA levels de-sensitizes *MYCN*-amplified neuroblastoma cells to ABT-263.

The relationship between *MYCN* and *PMAIP1* expression in *MYCN*-amplified neuroblastoma cells was significant (Figures 1C–1E and S1D, S1G, and S1H), raising the possibility that *MYCN* may regulate *PMAIP1* in these tumors. We investigated this hypothesis by knockdown of MYCN and found a concomitant decrease in NOXA protein (Figures 2C and S2C) and *PMAIP1* mRNA levels (Figure S2D). Furthermore, ectopic MYCN expression in *MYCN*-WT neuroblastoma cells or in epithelium-derived RPE-1 cells led to a concomitant increase in NOXA at both the protein and RNA levels (Figures 2D and 2E). In contrast, reducing MYCN levels in *MYCN*-amplified neuroblastoma cells or increasing MYCN in *MYCN*-WT neuroblastoma cells did not consistently affect the expression of other BCL-2 family proteins other than *PMAIP1* (Figures S2E and S2F), nor did MYCN levels correlate with other BCL-2 family

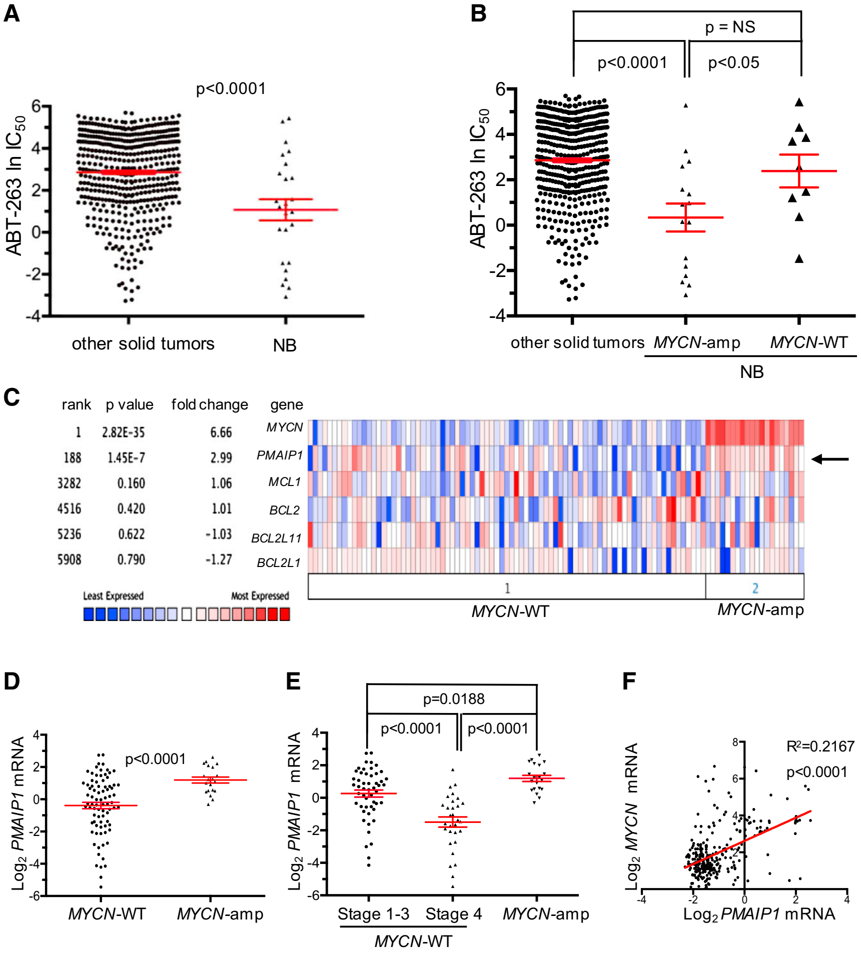

Figure 1. *MYCN*-Amplified Neuroblastoma Cells Have Sensitivity to the BCL-2/Bcl-xL Inhibitor ABT-263 and High NOXA Expression

(A) The sensitivity of ABT-263 in neuroblastoma (NB) cell lines (n = 25) is plotted against other solid tumor cancer cell lines (n = 476). Student's t test.

(B) Sensitivity of *MYCN*-amplified neuroblastomas (n = 16), *MYCN*-WT neuroblastomas (n = 9) and other solid tumor cancer cell lines (n = 476) to ABT-263. Student's t test.

(C) Heatmap from data from Wang et al. (2006) showing RNA levels of MYCN and select BCL-2 family members from 20 *MYCN*-amplified and 81 *MYCN*-WT neuroblastomas. Arrow indicates NOXA (*PMAIP1*). The Oncomine Platform was used for analysis and visualization.

(D) *PMAIP1* mRNA levels in 20 *MYCN*-amplified versus 81 *MYCN*-WT neuroblastoma. Non-parametric Mann-Whitney U-test, $p < 0.0001$ (Wang et al., 2006).

(E) *PMAIP1* mRNA levels in stage 1–3 *MYCN*-WT (n = 51) versus stage 4 *MYCN*-WT (n = 30) neuroblastoma, non-parametric Mann-Whitney U-test, $p < 0.0001$; stage 1–3 WT versus *MYCN*-amplified neuroblastoma, non-parametric Mann-Whitney U-test, $p = 0.0188$; and stage 4 WT versus *MYCN*-amplified neuroblastoma, non-parametric Mann-Whitney U-test, $p < 0.0001$ (Wang et al., 2006).

(F) *PMAIP1* RNA expression plotted against *MYCN* RNA expression in 285 medulloblastoma tumors (Northcott et al., 2012). Linear regression analysis, $R^2 = 0.2167$, $p < 0.0001$. Red lines are the mean for (A), (B), (D), and (E) and error bars are ±SEM. Y axis is Log_2 median-centered for (D) and (E), and both axes are Log_2 median-centered for (F). See also Figure S1; Table S1.

member expression in the Garnett cell line collection (Figures S2G–S2I). These data indicate that *MYCN* amplification in neuroblastoma cells upregulates NOXA expression and contributes to ABT-263 sensitivity.

We further assessed whether MYCN was causative of ABT-263 sensitivity. Upon ABT-263 treatment, the exogenous MYCN-expressing WT neuroblastoma cells demonstrated a marked increase in cleaved PARP expression compared with the GFP-expressing controls; consistently, these cells had enhanced sensitivity to ABT-263 in 3-day viability assays (Figures 2F and 2G). Conversely, the shMYCN-transduced *MYCN*-amplified neuroblastoma SK-N-BE(2) and SK-N-DZ cells were de-sensitized to ABT-263 compared with the control cells (Figures 2H and 2I). These data further demonstrate a causal role of MYCN in ABT-263 sensitivity in neuroblastoma. We next probed whether MYCN-regulation of *PMAIP1* was direct. Chromatin immunoprecipitation assays revealed preferential binding of the MYCN antibody over an isotype-matched IgG control within the promoter of *PMAIP1* similar to well-characterized MYCN-binding sites in *MDM2* and *MIR17HG* (Figure 2J) (Shohet et al., 2011; Slack et al., 2005). One site, BS1, includes the reported MYCN-preferential *CATGTG* motif, and BS2 includes the *CAACTG* motif that MYCN shows a proclivity toward when amplified (Murphy et al., 2009). These data altogether demonstrate that amplified *MYCN* sensitizes neuroblastoma cells to ABT-263, and this involves MYCN-dependent upregulation of *PMAIP1* transcription.

MYCN-Amplified Neuroblastoma Cells Retain Sensitivity to ABT-199

Despite its clinical efficacy in certain hematologic cancers, the utility of ABT-263 may be hampered by on-target thrombocytopenia (Roberts et al., 2012) that is a result of BCL-xL inhibition in platelets (Mason et al., 2007). ABT-199 (venetoclax) is a next-generation BH3 mimetic designed to spare BCL-xL (Souers et al., 2013); however, despite the obvious benefit of this drug in avoiding thrombocytopenia, its utility may largely be dictated by a cancer's propensity to rely on BCL-2 over BCL-xL for survival. Toward this end, we performed gene expression analysis of BCL-2 and BCL-xL in the Cancer Cell Line Encyclopedia (Barretina et al., 2012). BCL-2 expression was significantly higher in neuroblastoma cells compared with other solid tumor cell lines, and BCL-xL expression was significantly reduced in neuroblastoma cells, resulting in a high BCL-2:BCL-xL ratio (Figure 3A). We therefore hypothesized that the unique sensitivity to ABT-263 seen in *MYCN*-amplified cells would be preserved following ABT-199 treatment; in fact, we found that *MYCN*-amplified neuroblastoma cells remained sensitive to ABT-199, while *MYCN*-WT

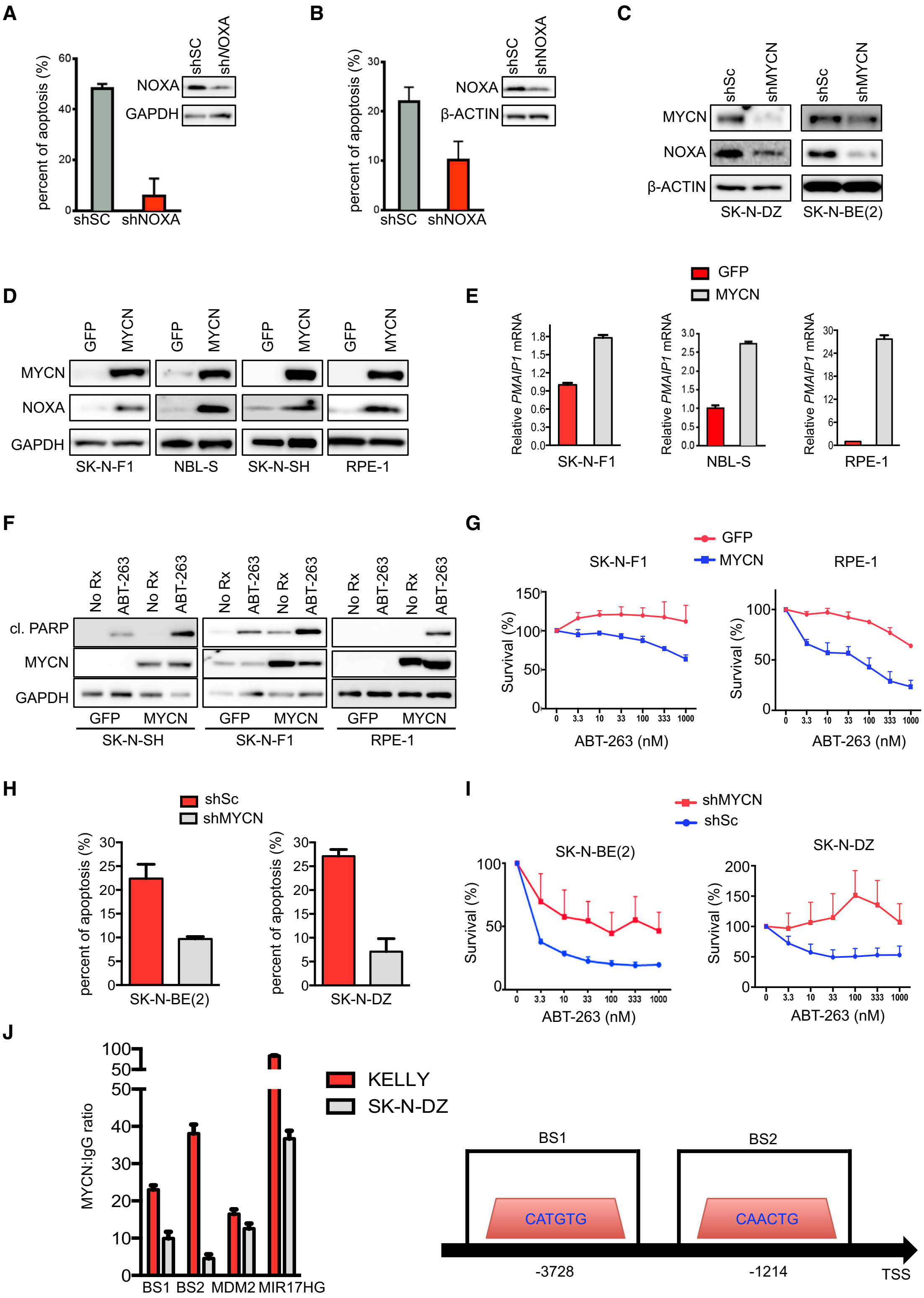

Figure 2. *MYCN*-Amplified Neuroblastoma Cells' Sensitivity to ABT-263 Is Mitigated by NOXA Reduction

(A and B) FACS apoptosis quantification of *MYCN*-amplified neuroblastoma cell lines SK-N-DZ (A) and SK-N-BE-2 (B) infected with scrambled (Sc) or NOXA-specific (NOXA) shRNA, n = 3, error bars are + SEM. Percent of apoptosis induced by ABT-263 minus the no treatment control. Inset = Western blot of knockdown.

(legend continued on next page)

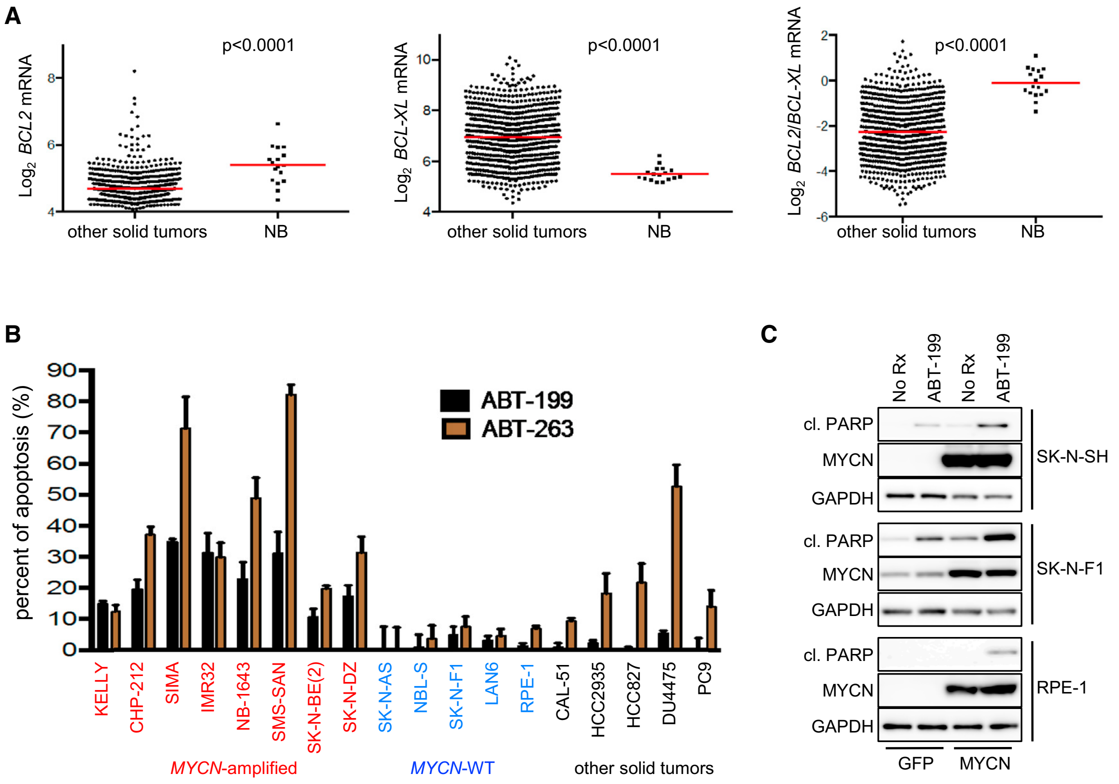

Figure 3. *MYCN*-Amplified Neuroblastoma Cells Retains Sensitivity to ABT-199 through a High BCL-2/BCL-xL Ratio

(A) *BCL2* (left), *BCL-xL* (middle), or the ratio of *BCL2/Bcl-xL* (right) from the Cancer Cell Line Encyclopedia were plotted for solid tumor cancer cell lines (n = 840) and neuroblastoma cell lines (n = 17). Student's t test, $p < 0.0001$ for all comparisons. Red lines are means.

(B) FACS apoptosis determination of indicated cell lines following 72 hr of ABT-199 or ABT-263 treatment over no-treatment control. Error bars are + SD, n = 3.

(C) *MYCN*-WT neuroblastoma SK-N-SH, SK-N-F1, and the RPE-1 cells expressing GFP or MYCN were left untreated or treated with ABT-199 for 24 hr and the amount of cleaved PARP was determined.

See also Figure S3.

neuroblastoma cells and RPE-1 cells were insensitive to ABT-199, consistent with their insensitivity to ABT-263 (Figures 3B and S3A). In the other solid tumor cell lines with a range of sensitivity to ABT-263, all lines were resistant to ABT-199 (Figures 3B and S3A). Similar to the data with ABT-263 (Figure 2F), exogenous expression of MYCN markedly increased sensitivity to ABT-199 in *MYCN*-WT neuroblastoma cells and RPE-1 cells (Figure 3C).

Combination Drug Screen Reveals that Dual BCL-2-Aurora A Kinase Inhibition Therapy Is Effective

ABT-199 has exhibited a relatively favorable toxicity profile (Seymour et al., 2014; Ma et al., 2014), and it uniquely induced apoptosis in *MYCN*-amplified neuroblastoma cells (Figures 3B and S3A); we therefore decided to pursue the drug as part of a combination targeted therapy strategy for *MYCN*-amplified neuroblastoma. We performed a combination drug screen using ABT-199 as the "anchor" in combination with 24 targeted therapies covering a wide array of targets (Table S2). We found that both concentrations of the Aurora A inhibitor MLN8237 (alisertib) and the Aurora A/B inhibitor VX680 (tozasertib) potently induced apoptosis in *MYCN*-amplified neuroblastomas when combined with ABT-199 (Figures 4A and 4B). Furthermore, both combinations potently decreased the viability of KELLY cells (Figure 4C), consistent with the apoptosis screen (Figure 4A); strong apoptotic responses were also detected when assayed by fluorescence-activated cell sorting (FACS) (Figure S3B). The Bliss

(C) Cell lysates from cells infected with a control shRNA (shSc) or shMYCN were immunoblotted for MYCN, NOXA, and β-ACTIN.

(D and E) *MYCN*-WT neuroblastoma cells and the epithelium-derived RPE-1 cells were engineered to overexpress GFP or MYCN and lysates probed for MYCN, NOXA, or β-ACTIN (D), or levels of the abundance of *PMAIP1* RNA (E) (relative to *ACTB*). n = 3; error bars are +SD.

(F and G) *MYCN*-WT cells expressing GFP or MYCN were left untreated or treated with ABT-263 for 24 hr and the amount of cleaved PARP determined (F), or for 72 hr and number of viable cells determined (G). For (G), n = 3. Error bars are +SD.

(H and I) SK-N-BE(2) and SK-N-DZ shSC and shMYCN cells (as in (C)) were treated with ABT-263 for 72 hr and the amount of apoptosis was determined (H), or 72 hr and viable cells determined (I). For (I), n = 3. Error bars are +SD.

(J) Ratio of SYBR green signal from a chromatin immunoprecipitation assay with an MYCN antibody over an isotype-matched IgG in the KELLY and SK-N-DZ cells (left) and schematic of the regions amplified on the *PMAIP1* promoter where MYCN bound (right). Error bars are + SD.

See also Figure S2.

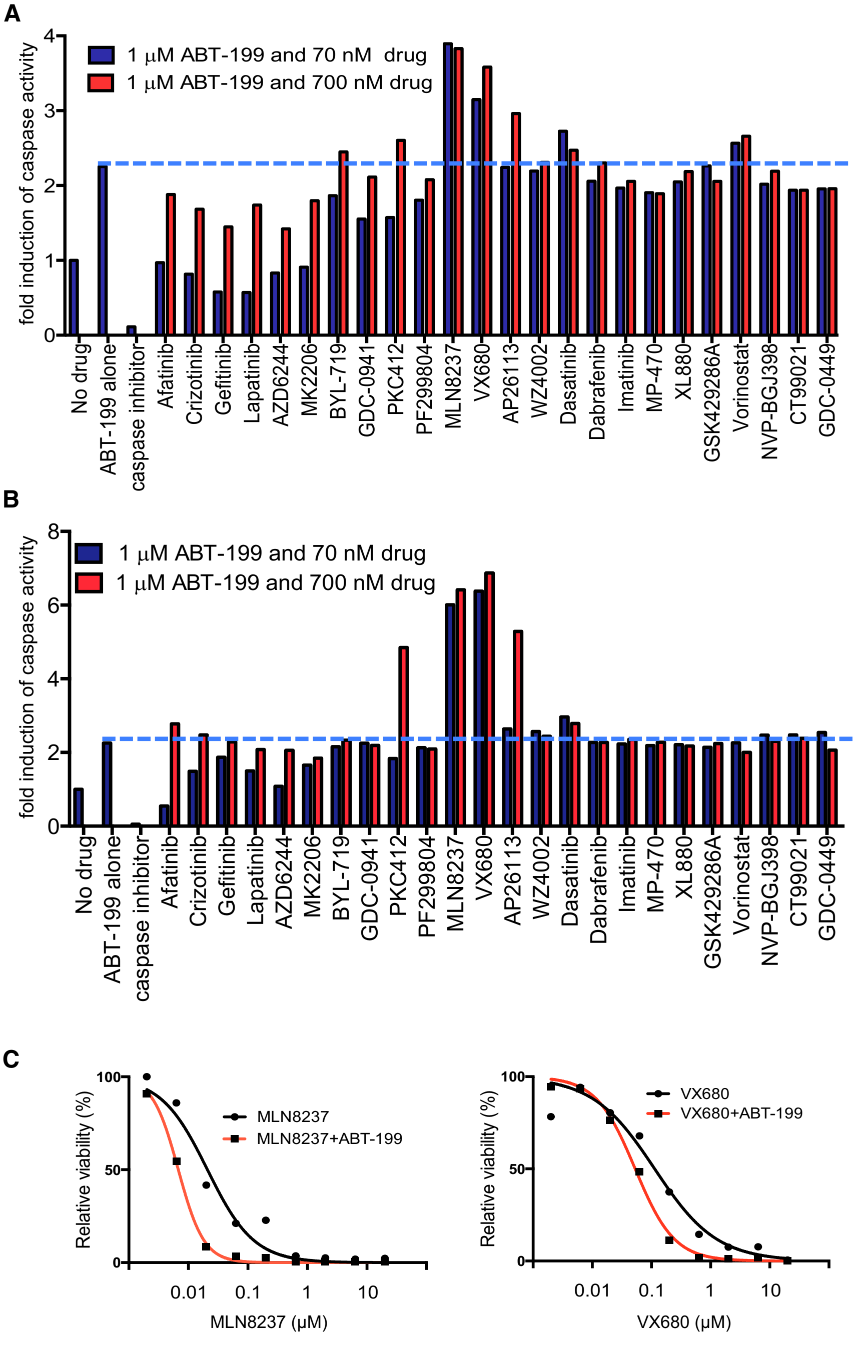

Figure 4. Aurora A Inhibition Combines with ABT-199 to Promote Apoptosis and Induce Loss of Cell Viability

(A and B) Anchor apoptosis drug screen with 1 μM ABT-199 and 24 other targeted therapies at two different doses (blue bars, 70 nM; red bars, 700 nM) in KELLY (A) and SK-N-DZ (B) cells. As controls, cells were also treated with no drug, 1 μM ABT-199 alone and 1 μM ABT-199 plus caspase inhibitor, as indicated. Blue dotted line drawn at amount of caspase activity from single-agent ABT-199 for visual comparison. Caspase activity is plotted on the y axis.

(C) 72 hr viability assay of KELLY cells treated with increasing amounts of MLN8237 (left) or VX680 (right) in the absence or presence of 1 μM ABT-199. See also Figures S4–S6; Table S2.

independence model confirmed a marked synergistic interaction between MLN8237 and ABT-199 at relatively low concentrations of MLN8237 (Figure S3C). Consistent with the notion that both growth arrest and apoptosis are required for effective targeted therapy (Faber et al., 2014; Hata et al., 2014, 2015), the addition of MLN8237 to ABT-199 resulted in growth arrest (Figures S4A and S4B). These data indicate that Aurora A inhibition further sensitizes *MYCN*-amplified neuroblastoma cells to ABT-199.

Since MLN8237 showed single-agent activity in several neuroblastoma mouse xenograft models during experiments conducted by the Pediatric Preclinical Testing Program, leading to testing in pediatric neuroblastoma patients (Maris et al., 2010), and MLN8237 was more potent at a lower dose than the dual Aurora inhibitor VX680 when combined with ABT-199 (Figure 4C), we decided to pursue ABT-199/MLN8237 combinations in *MYCN*-amplified neuroblastoma cells. MLN8237 is an in-clinic Aurora A inhibitor with high specificity over Aurora B (Manfredi et al., 2011). We verified both single-agent efficacy of the two drugs as well as the apoptosis-inducing potency of the combination across a panel of *MYCN*-amplified neuroblastoma cells compared with both the *MYCN*-WT neuroblastoma cells and the RPE-1 cell line, where it was ineffective (Figure 5A, 57.6 ± 8.1 versus 5.8 ± 2.9, Student's *t* test, p = 0.0015).

MLN8237 Enhances ABT-199 Activity in Part through MCL-1 Reduction in *MYCN*-Amplified Neuroblastoma Cells

We next sought to understand how the addition of MLN8237 further sensitized *MYCN*-amplified neuroblastoma cells to ABT-199. MLN8237 induced mitotic arrest (Figures S4A and S4B); interestingly, upon mitotic arrest, MCL-1 is phosphorylated and degraded in a proteasome-dependent manner (Harley et al., 2010; Haschka et al., 2015; Wertz et al., 2011). In addition, much of targeted-therapy-induced apoptosis is ultimately controlled by a balance of expression and function of the BCL-2 family members (Hata et al., 2015) and resistance to ABT-199 occurs through upregulation of BCL-xL and MCL-1 (Choudhary et al., 2015). Since BCL-xL levels are relatively low in neuroblastoma (Figure 3A) and *MYCN*-amplified neuroblastoma cells and tumors have high levels of the MCL-1 inhibitor NOXA (Figures 1C–1E, S1D, S1G, and S1H), we hypothesized that the further induction of

apoptosis seen when MLN8237 was combined with ABT-199 could be due to reduction of MCL-1 levels, effectively decreasing the apoptotic threshold even lower in these cancers. Therefore, we assessed MCL-1 expression following MLN8237 treatment. We found that MCL-1 expression was downregulated following MLN8237 treatment (Figure 5B and S4C). Interestingly, we found levels of phosphorylated 4E-BP1 were also reduced by MLN8237, following the same pattern as MCL-1 expression and phosphorylated Aurora A (Figures 5B and S4C). MCL-1 mRNA levels were elevated following treatment with MLN8237 (Figure S4D), which may be a result of feedback from protein loss (Faber et al., 2014). Furthermore, phosphorylated GSK3, corresponding to inactivation of the kinase, which stabilizes MCL-1 in a mitosis-independent matter (Maurer et al., 2006), was not inhibited by MLN8237, and in fact, was often upregulated (Figure S4E).

Inhibiting p4E-BP1 in other cancers leads to disruption of the eIF4G:eIF4E complex and downregulation of cap-dependent MCL-1 protein translation (Mallya et al., 2014; Mills et al., 2008; Schatz et al., 2011), which suggested that MLN8237 reduced MCL-1 expression in *MYCN*-amplified neuroblastoma cells via a shift toward mitotic arrest-inducing degradation and away from cap-dependent MCL-1 translation. Therefore, we sought to determine whether inhibition of mTORC1-p4E-BP1 leads to downregulation of MCL-1 in these cancers (Faber et al., 2014; Schatz et al., 2011). In fact, we found that treatment with the TORC1/2 inhibitor MLN0128 led to downregulation of p-4E-BP1 and MCL-1 in *MYCN*-amplified neuroblastomas (Figure S5A), which demonstrated that MCL-1 in these cancers is downregulated following inhibition of p4E-BP1. We verified that MLN8237 and VX680 treatment each resulted in loss of p4E-BP1 and MCL-1 in *MYCN*-amplified neuroblastoma cells (Figure S5B), as did knockdown of Aurora A (Figure S5C). These data altogether confirm a role of Aurora A in the downregulation of p4E-BP1, which results in the downregulation of MCL-1 in *MYCN*-amplified neuroblastoma cells. We next treated *MYCN*-amplified neuroblastoma cells with a short time course of MLN8237, and found p4E-BP1 and MCL-1 were reduced prior to marked mitotic arrest (Figures S5D and S5E), suggesting a contribution of MCL-1 downregulation independent of mitotic arrest. To more directly address whether inhibition of protein translation contributes to MCL-1 downregulation following MLN8237 treatment, we first measured the effects of MLN8237 on global protein translation in *MYCN*-amplified neuroblastoma cells (Goodman et al., 2011). We demonstrate a consistent decrease in protein translation across the cells (Figure S5F). To specifically determine whether inhibition of cap-dependent translation was contributing to the decrease in global protein synthesis, we performed a pull-down assay with m^7-GTP Sepharose beads to determine whether the levels of cap-bound proteins were decreased. Western blot analysis revealed disassociation of eIF4G:eIF4E, significant for inhibition of cap-dependent protein translation (Figure S5G), with the expected concomitant increase in 4EBP1:eIF4E complexes (Figure S5G). Therefore, in *MYCN*-amplified neuroblastoma, MLN8237 inhibits p4E-BP1, leading to loss of eIF4G:eIF4E complex-mediated MCL-1 protein translation.

We next sought to better define the role of MCL-1 in combination-induced toxicity. MCL-1 reduction by siRNA sensitized *MYCN*-amplified neuroblastoma cells to ABT-199, inducing similar levels of apoptosis as the combination treatment in the scrambled (sc) siRNA transfected cells (Figures 5C and S6B). Introducing exogenous MCL-1 inhibited apoptosis, as measured by cleaved PARP induced by combined ABT-199/MLN8237 (Figures 5D and S6A). The induction of apoptosis in *MYCN*-amplified neuroblastoma cells was mitigated by BIM siRNA (Figures 5C and S6C), implicating that the disruption of BIM/MCL-1 complexes leads to loss of MCL-1-bound BIM and contributes to ABT-199/MLN8237-mediated apoptosis. To directly assess this, we performed immunoprecipitation with antibodies against MCL-1 and BIM in SK-N-BE(2) and SK-N-DZ cell lysates following different drug treatments. We found that BIM:MCL-1 complexes are increased following ABT-199 treatment, which mitigates BIM-mediated apoptosis; however, addition of MLN8237 markedly reduced these complexes, consistent with the effect of MLN8237 on MCL-1 expression in whole-cell extracts (Figures 5E and S6D). These data altogether demonstrate that MLN8237 downregulation of MCL-1 contributes to the sensitivity of *MYCN*-amplified neuroblastoma cells to ABT-199/MLN8237 by disrupting BIM:MCL-1 complexes and that ABT-199/MLN8237 combination-induced apoptosis is, at least in part, mediated by BIM. Of interest, we found that MYCN protein levels were also reduced by MLN8237 treatment (Figure 5B), consistent with previous data (Brockmann et al., 2013) and likely contributing to combination efficacy.

Differential Apoptosis Induced by ABT-199/MLN8237 in *MYCN*-Amplified Compared with *MYCN*-WT Neuroblastoma Cells Translates to Potent Differences in Drug Efficacy

To characterize the relevance of the differential apoptotic response seen in the combined ABT-199/MLN8237 treatment between *MYCN*-amplified and *MYCN*-WT neuroblastoma cells (Figure 5A), we treated cell lines with each inhibitor alone or in combination and assayed viability after 5 days (Figures 5F and 5G). After 5 days of treatment, cells treated with the combination demonstrated marked growth inhibition in the *MYCN*-amplified neuroblastoma cells compared with *MYCN*-WT cells; the single agents had limited and variable effects across the *MYCN*-amplified cell lines (Figure 5F). Consistent with the apoptosis data (Figure 5A), the combination had a limited effect on *MYCN*-WT neuroblastoma cells and the RPE-1 cell line (Figure 5G). Similarly, in 6-day viability assays, when cells were treated with increasing doses of MLN8237, the combination of ABT-199 and MLN8237 was markedly more effective in *MYCN*-amplified neuroblastoma cells compared with the *MYCN*-WT neuroblastoma cells and the RPE-1 cell line (Figures S6E and S6F). It is noteworthy that similar levels of efficacy were demonstrated with the ABT-199/MLN8237 combination as the ABT-263/MLN8237 combination in the *MYCN*-amplified neuroblastoma cells (Figures S6E and S6F). These data are consistent with the expression data supporting the use of the Bcl-xL-sparing ABT-199 in *MYCN*-amplified neuroblastoma (Figure 3A) and the ability of ABT-199 to induce apoptosis in these cancers (Figure 3B). Lastly, we noted that this combination was effective at even low concentrations of MLN8237 (10 nM MLN8237) in the *MYCN*-amplified neuroblastoma cells

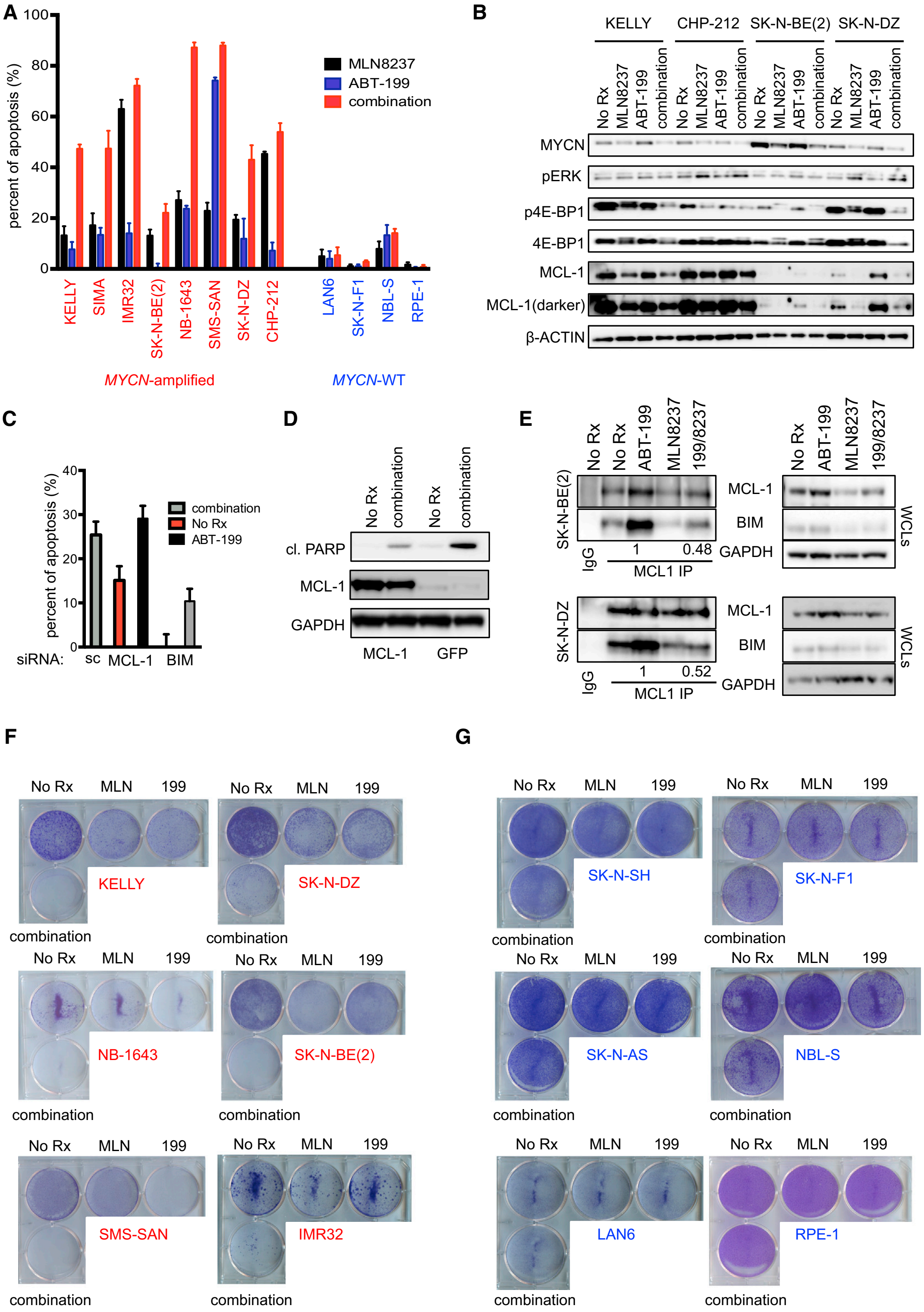

Figure 5. ABT-199/MLN8237 Is Effective in *MYCN*-Amplified Neuroblastoma Cells through Enhanced Apoptosis

(A) FACS apoptosis determination of indicated cell line treated for 48 hr with the indicated treatments. Percent of apoptosis induced by the drugs minus the no treatment control. Error bars are SD (n = 3).

(B) Western blot analysis of *MYCN*-amplified neuroblastomas treated with no drug (No Rx), MLN8237, ABT-199, or the combination.

(C) SK-N-BE(2) cells transfected with scrambled (sc) siRNA, MCL-1 siRNA, or BIM siRNA were treated as indicated in the figure. FACS apoptosis is presented as the amount of apoptosis for each condition minus no treatment of the scrambled control. Error bars are +SEM.

(legend continued on next page)

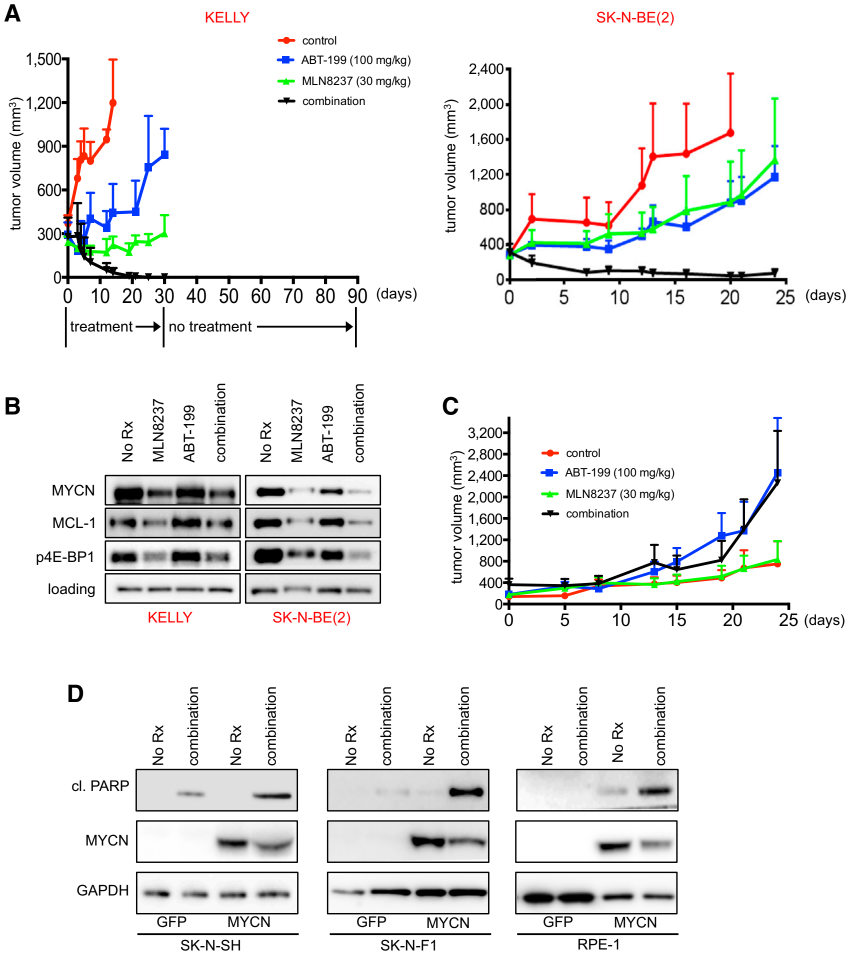

Figure 6. ABT-199/MLN8237 Is Effective in Mouse Models of *MYCN*-Amplified Neuroblastoma

(A) Mice were treated with MLN8237, ABT-199, or both drugs for the indicated times. The cohort of KELLY mice treated with the combination was monitored for an additional 60 days. Error bars are + SEM.

(B) Western blot analysis of tumor lysates from KELLY-bearing and SK-N-BE(2)-bearing mice with the indicated treatments (loading = GAPDH or β-actin).

(C) SK-N-SH tumors were treated and monitored as in (A). Error bars are +SEM.

(D) *MYCN*-WT neuroblastoma SK-N-SH, SK-N-F1, and the RPE-1 cells expressing GFP or MYCN were left untreated or treated with MLN8237/ABT-199 (combination) for 24 hr and lysates were blotted with the indicated antibodies.

See also Figure S7.

(Figures 4C, 5F, and S6E). Together, these data show a specific and potent in vitro efficacy of ABT-199/MLN8237 in *MYCN*-amplified neuroblastoma cells, even at relatively low concentrations of MLN8237.

ABT-199/MLN8237 Exhibits Robust In Vivo Activity against *MYCN*-Amplified Neuroblastoma

Based on the in vitro specificity and potency of this combination, we pursued studies in animal models of neuroblastoma. *MYCN*-amplified KELLY and SK-N-BE(2) xenograft-bearing mice were treated with 30 mg/kg once daily MLN8237, 100 mg/kg once daily ABT-199, or the combination, and tumor progression was monitored. There was no reduction in mouse body weight (Figure S7A). Neither MLN8237 nor ABT-199 alone regressed these tumors, whereas mice treated with the combination exhibited tumor regression (Figure 6A). KELLY xenografts in mice treated with the combination regressed to an undetectable level and remained undetectable for 60 days following the last treatment. Based on previous criteria (Laude et al., 1991), we considered this a durable and lasting tumor-free response (Figure 6A). Analysis of tumors treated with MLN8237 and ABT-199 showed that the combination reduced the levels of MCL-1 and p4E-BP1 in vivo, consistent with our in vitro observations (Figure 6B). In contrast, the *MYCN*-WT SK-N-SH mouse xenograft model was insensitive, and in fact tumors treated with ABT-199 appeared to grow faster than the no-treatment controls (Figure 6C). Again, the combination therapy was well tolerated by evidence of weight sustainability (Figure S7B). In vitro modeling of sensitivity to the combination revealed MYCN expression was sufficient to sensitize to the combination (Figure 6D), consistent with our data from the single-agent BCL-2 inhibitors (Figures 2F, 2G, 3C).

MLN8237 has been tested in a phase I clinical trial conducted within the Children's Oncology Group (Mosse et al., 2012), which revealed an optimal dosing schedule of 80 mg/m^2 daily for 7 days, followed by 14 days off treatment, repeated every 3 weeks. Therefore, we performed additional in vivo experiments at the clinically relevant dosing schedule (Mosse et al., 2012). In the *MYCN*-amplified human xenograft mouse model of NB-1643, ABT-199 was administered daily at 100 mg/kg, and MLN8237 was administered at 30 mg/kg, 7 days on, 14 days off. Following this dosing schedule, the NB-1643 model displayed tumor stasis with single-agent MLN8237 treatment, significant tumor regression with single-agent ABT-199 treatment, and complete regression of all tumors with the combination (Figure 7A); these mice were followed for an additional 60 days

(D) Exogenous overexpression of MCL-1 in SK-N-BE(2) cells and PARP cleavage following combination treatment.

(E) MCL-1 complex immunoprecipitation in the SK-N-BE(2) and SK-N-DZ cells. An IgG matched isotype antibody served as a control. Ratio of quantified band intensities of BIM complexed to MCL-1/normalized BIM amounts in the whole-cell lysates (WCLs).

(F and G) Crystal violet staining of 5-day growth assays of *MYCN*-amplified (F) or *MYCN*-WT neuroblastoma cells or RPE-1 cells (G) left untreated (No Rx), or treated with MLN8237, ABT-199, or the combination.

See also Figures S4–S6.

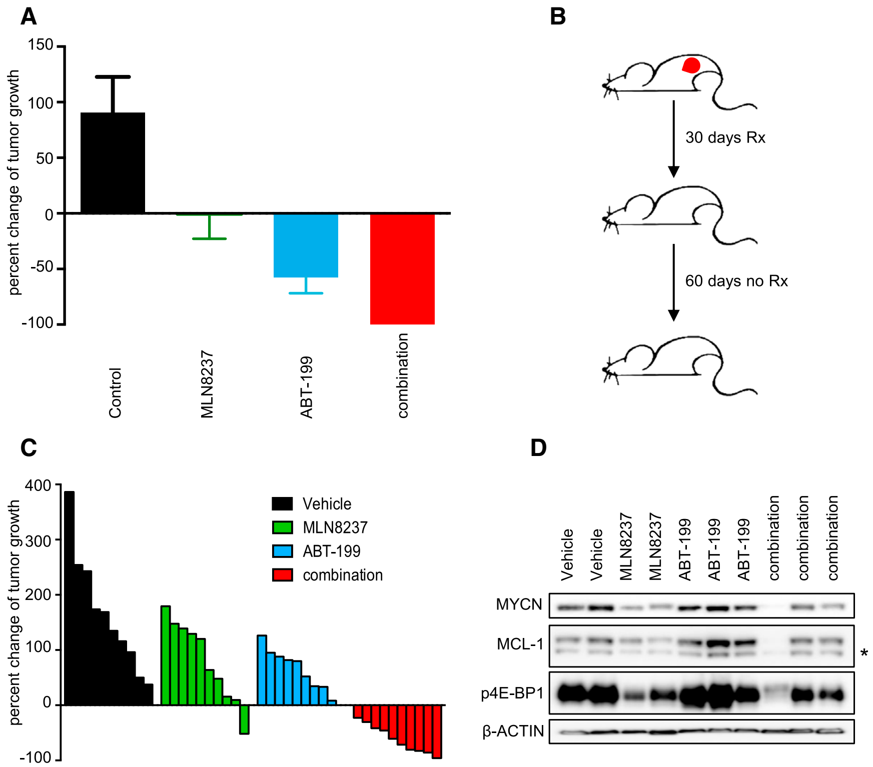

Figure 7. ABT-199/MLN8237 Combination Is Effective at the Optimal Dosing Schedule of MLN8237 in *MYCN*-Amplified Neuroblastoma Models

(A) NB-1643 mice were treated with single-agent MLN8237, single-agent ABT-199, or the combination and monitored for approximately 30 days. Error bars are +SEM.

(B) All tumors from the combination cohort were monitored an additional 60 days and did not demonstrate re-growth.

(C) Tumors (each represented by a bar) from individual mice bearing PDXs were monitored for growth after treatment with vehicle (control), MLN8237, ABT-199, or the combination for approximately 30 days.

(D) Western blots from tumor lysates from the PDXs. Asterisk indicates non-specific band.

See also Figure S7.

following completion of treatment, during which the tumors remained undetectable (Figure 7B).

The implementation of patient-derived cell lines and patient-derived xenografts (PDXs) in preclinical studies has been helpful to evaluate and improve cancer therapeutics (Crystal et al., 2014; Faber et al., 2015). Here, we have established *MYCN*-amplified neuroblastoma PDXs via injection of fresh tumor from a neuroblastoma patient into CB17 SCID mice for preclinical evaluation of this combination therapy. Following the same dosing schedule for MLN8237 of 1 week on, 2 weeks off, mice treated with 100 mg/kg once daily ABT-199, 30 mg/kg MLN8237, or the combination, were evaluated. While both single agents had modest activity in this model, the combination induced regression of ten out of ten tumors in the cohort (Figure 7C). In these PDXs, there was a notable increase in MCL-1 expression following ABT-199 treatment, and MLN8237 led to reduction in MCL-1 from both baseline levels and levels following ABT-199 treatment (Figure 7D), similar to the in vitro results (Figure 5B) and the KELLY tumors (Figure 6B). The combination-treated mice showed no reduction in weight gain during the treatment period (Figure S7C), consistent with the mice from the Nu/Nu mouse studies (Figures S7A and S7B). These data suggest that the combination therapy can induce sustained remissions in a PDX model harboring *MYCN* amplification using a clinically feasible schedule of MLN8237 and ABT-199 administration, supporting its promising preclinical profile and proposed mode of action (Figure 8).

DISCUSSION

Amplification of *MYCN* is the driving oncogenic event in a subset of the most aggressive neuroblastomas; exposure to MYCN antisense oligonucleotides results in tumor growth inhibition (Burkhart et al., 2003). As a non-kinase, however, drugging MYCN with small molecule inhibitors has proven challenging. Recently, several groups reported that indirectly targeting MYCN resulted in anti-tumor activity, further validating the pathway as an important target (Chipumuro et al., 2014; Gustafson et al., 2014; Puissant et al., 2013). One such approach used MLN8237, which induces proteasome-mediated degradation of MYCN (Brockmann et al., 2013) and leads to loss of its expression (Brockmann et al., 2013).

In this study, we took a pharmacogenomic approach to identify an effective combination-based targeted therapy for *MYCN*-amplified neuroblastoma. This approach yielded several important findings. First, *MYCN*-amplified neuroblastoma cells are exquisitely sensitive to the BCL-2/BCL-xL inhibitor ABT-263. MYCN directly regulates the expression of NOXA in these cancers, with amplified *MYCN* leading to high expression of NOXA. Second, due to high BCL-2/BCL-xL ratios in these cancers, *MYCN*-amplified neuroblastoma cells retain sensitivity to the BCL-2 inhibitor ABT-199. Third, the Aurora Kinase A inhibitor MLN8237 enhances efficacy of ABT-199 in part by inducing mitotic arrest and downregulating MCL-1, disrupting ABT-199 induced BIM:MCL-1 complexes. Fourth, in multiple mouse models of *MYCN*-amplified neuroblastoma, complete and sustained regressions were noted, without any overt signs of toxicity to the mice.

MYCN is a promiscuous transcription factor that prefers *CATGTG* and *CACGTG* E-BOX sites; moreover, amplified *MYCN* binds additional E-BOX sites, including a propensity towards *CAACTG* (Murphy et al., 2009). Here, we found that MYCN bound two regions in the *PMAIP1* promoter, encompassing a *CATGTG* E Box and a *CAACTG* E Box.

MYCN-induced upregulation of NOXA in neuroblastoma models adds to the list of other *MYCN*-regulated pro-apoptotic processes in this disease (Chen et al., 2010; Fulda et al., 1999; Petroni et al., 2011; Veschi et al., 2012). Debatin and colleagues

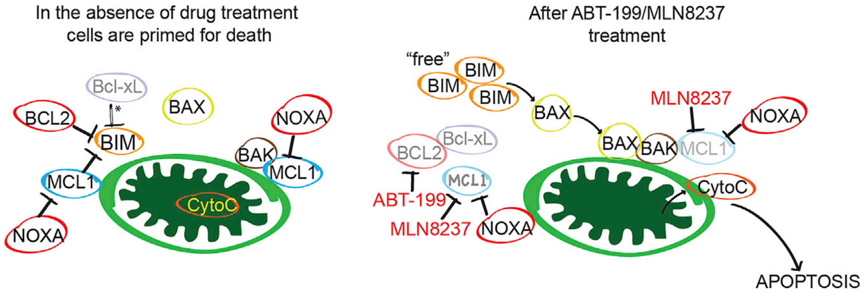

Figure 8. Schematic of *MYCN*-Amplified Neuroblastomas before and after Treatment with the ABT-199/MLN8237 Combination

Asterisk denotes weak interaction due to low BCL-xL levels.

In *MYCN*-amplified neuroblastoma, MYCN upregulates NOXA to inhibit MCL-1 and these tumors have low BCL-xL levels. The addition of ABT-199 inhibits BCL-2 with further reduction of MCL-1 by MLN8237, leading to the liberation of BIM and apoptosis. Modified from Faber et al. (2014).

demonstrated that while overexpression of MYCN in neuroblastoma does not induce apoptosis, it primes the cell for cytotoxic drugs to induce apoptosis through cooperative upregulation of BAX (Fulda et al., 1999). Therefore, it is likely that *MYCN*-driven anti-apoptotic signals are particularly critical for tumorigenesis in neuroblastoma in order to counter these pro-apoptotic signals (Hogarty, 2003). For instance, caspase 8 is often silenced in neuroblastoma, and in TH-MYCN mice, which express MYCN in neural crest cells that form neuroblastomas; caspase 8 deficiencies cause formation of metastatic neuroblastomas in the bone marrow (Teitz et al., 2013). Consistent with a role of amplified *MYCN* in BCL-2 family-mediated apoptosis following cellular stress, BCL-2 overexpression from serum deprivation and metabolic stress can counter *MYCN*-mediated apoptotic signaling in neuroblastoma and, therefore, cooperate in tumorigenicity (Jasty et al., 2001; Ushmorov et al., 2008). In addition, *MYCN* amplification participates in killing of neuroblastoma cells following glutamine deprivation, and this pathway involves NOXA (Qing et al., 2012).

In our therapeutic approach, ABT-199 eliminates this pro-survival BCL-2 signal, thus allowing for MYCN-mediated pro-apoptotic signaling to help push the neuroblastoma cells toward the apoptotic threshold. MLN8237 further pushes the neuroblastoma cell past the apoptotic threshold through reduction of MCL-1; BCL-xL is ineffective at blocking the ensuing death signal because BCL-xL levels are markedly low. The benefit of MYCN reduction by MLN8237 outweighs any relief of a pro-apoptotic signal, consistent with the anti-cancerous effects of targeting MYCN alone in *MYCN*-amplified neuroblastoma. Altogether, the combination therapy reduces cell viability and induces widespread apoptosis in vitro, with in vivo tumor regressions demonstrated in preclinical models. Finally, since drug treatments were well tolerated in multiple mouse strains, the combination therapy appears to have a favorable toxicity profile.

Prolonged mitotic arrest leads to loss of MCL-1 (Harley et al., 2010; Haschka et al., 2015), as does downregulation of p-4E-BP1, which mediates eIF4 complex formation and subsequent cap-dependent translation (Faber et al., 2014, 2015; Hsieh et al., 2010; Mallya et al., 2014; Mills et al., 2008; Schatz et al., 2011). Here, we have demonstrated that Aurora A inhibition not only leads to mitotic arrest but also leads to loss of p4E-BP1 in *MYCN*-amplified neuroblastoma cells (resulting in downregulation of MCL-1 as a result of cap-dependent translation inhibition). Therefore, it is likely that both mitotic arrest and loss of p4E-BP1-mediated cap-dependent protein translation contribute to the loss of MCL-1 following MLN8237 treatment in *MYCN*-amplified neuroblastoma cells.

To substantiate our in vitro and in vivo human xenograft findings, we found that the combination is effective in a *MYCN*-amplified neuroblastoma PDX model, which demonstrated only modest single-agent MLN8237 activity, mirroring clinical activity of MLN8237 in neuroblastoma (Mosse et al., 2012). Altogether, this combination should be prioritized for clinical testing in patients with the *MYCN*-amplified subset of neuroblastoma.

EXPERIMENTAL PROCEDURES

Drug Screen

The drug screen in which the ABT-263 sensitivity data was obtained (Figures 1A and 1B) comparing neuroblastoma sensitivity with other solid tumor cell lines has been described previously (Garnett et al., 2012).

Targeted Therapy Anchor Screen

SK-N-DZ and KELLY cells were plated at 2,000 cells/well in 96-well plates in RPMI medium containing 10% fetal bovine serum. Cells were treated with either 1 μM ABT-199, 50 μM Z-FAD-FMK caspase inhibitor, 1 μM ABT-199 and 70 nM test drug, or 1 μM ABT-199 and 700 nM test drug. After 24 hr, caspase-3/7 levels were determined using the Caspase-Glo 3/7 Assay (Promega) following the manufacturer's instructions. Briefly, plates were allowed to equilibrate to room temperature and an equal volume of Caspase-Glo 3/7 Reagent was added to each well. Plates were mixed at 500 rpm for 30 s, incubated at room temperature, and luminescence read after 30 min.

Chromatin Immunoprecipitation

Chromatin from ~25 × 10^6 KELLY and SK-N-DZ cells were purified and sheared on a Diagenode Bioruptor. Cycle thresholds reached from SYBR green incorporation were calculated from 5 ng of chromatin per qPCR reaction. Primers were BS1, 5-tgctgggattacagacgtga-3′ (forward), gagttcgagaccagcct gac (reverse), originally described in Nikiforov et al. (2007); BS2, 5-caggttca agcgattctcgt-3′ (forward), ataccagccttgccaatatg-3 (reverse); MDM2, 5-agcc tttgtgcggttcgtg-3′ (forward), 5-ccccgtgacctttaccctg-3′ (reverse); *MIR17HG*, 5-tctcccgagggcgagagttaaagc-3′ (forward), 5-caccctcgcgcgtacaaagtttgg-3′ (reverse).

Xenograft Studies

Nu/Nu mice were injected with ~5 × 10^6 cells/100 μl of PBS, in combination with 100 μl of Matrigel. Mice were injected subcutaneously and monitored for tumor growth. When tumors reached ~200–400 mm^3, the tumor-bearing-mice were randomized to a no-treatment control group, a MLN8237 group (30 mg/kg), an ABT-199 group (100 mg/kg), or a combination group (same doses). Mice in the treatment cohorts (n = 3–6) were subsequently treated with drugs directly to the stomach by oral gavage. The solvent for MLN8237 was 10% 2-hydroxypropyl-β-cyclodextrin and 1% Na butyrate. The solvent for ABT-199 was 60% Phosal, 30% PEG 400, 10% EtOH. During the study, tumors were routinely measured by electronic caliper two to three times a week, in two dimensions (length and width), and with the formula $v = l \times (w)^2(\pi/6)$, where v is the tumor volume, l is the length, and w is the width. The drug schedules were once daily for the different xenograft models except NB-1643, which was once daily for MLN8237 for 1 week, with 2 weeks off MLN8237, and MLN8237 treatment resuming on week 4. For pharmacodynamic studies,

tumor-bearing mice were treated for 3 days, tumors were harvested ∼2–3 hr following the last treatment, and tumors were snap frozen in liquid nitrogen. All these mouse experiments were approved and performed in accordance with the Institutional Animal Care and Use Committee at the Massachusetts General Hospital.

Patient-Derived Xenografts

The PDX tumor tissue was obtained through the Children's Oncology Group Cell Culture and Xenograft Repository. CB17 SCID mice were subcutaneously implanted with *MYCN*-amplified COG-N-471x PDX tumors and monitored for tumor growth. Once the tumors reached a median tumor volume of 230–240 mm^3, PDX-bearing mice were randomized into statistically identical cohorts (10 mice/group) and treated continuously with 100 mg/kg once daily of ABT-199; 30 mg/kg once daily of MLN8237 for 1 week, followed by 2 weeks without treatment and the cycle resumed on week 4; the combination of ABT-199 100 mg/kg once daily being given continuously with the MLN8237 30 mg/kg once daily dosed for 1 week with 2 weeks break; appropriate vehicle control of 60% Phosal50, 30% PEG400, 10% EtOH as ABT-199 vehicle and 10% hydroxypropyl-β-cyclodextrin, 1% Na butyrate as vehicle for MLN8237 by oral gavage in 100 μl/10 g of body weight. Tumor size was measured by caliper every 3–4 days, and volume was calculated by the spheroid formula: $(p/6) \times d^3$, where d represents the mean diameter. The work on the PDX is considered non-human subjects research. All animal experiments were conducted according to relevant national and international guidelines. The PDX study in CB17 SCID mice was approved by the Children's Hospital of Philadelphia Institutional Animal Care and Use Committee (IACUC protocol # IAC-15-000643).

Database Analyses

All the gene expression datasets (besides the cancer cell line encyclopedia) in this study were from Oncomine (www.oncomine.org) and were downloaded and analyzed using Oncomine premium research edition. The gene expression data in Oncomine is Log_2 transformed and median-centered as in Figures 1C–1F, Figures S1A–S1G and Figures S2G–S2I.

Statistical Considerations

IC_{50} values for cell lines as presented in Figures 1A and 1B were calculated as previously described in the drug screens (Garnett et al., 2012). Non-parametric Mann-Whitney U-tests were performed for Figures 1D and 1E and Figures S1A–S1C. The statistical determinations from Table S1 are described in detail (Yang et al., 2013). Student's *t* test (two-sided) and linear regression analysis were performed using GraphPad Prism. Differences were considered statistically different if $p < 0.05$.

SUPPLEMENTAL INFORMATION

Supplemental Information includes Supplemental Experimental Procedures, seven figures, and two tables and can be found with this article online at http://dx.doi.org/10.1016/j.ccell.2016.01.002.

AUTHOR CONTRIBUTIONS

Conceptualization, J.H., C.C., C.H.B., J.A.E., Y.P.M., and A.C.F.; Methodology, J.H., C.C., E.M.S., T.L., M.L.B., A.D., R.S., A.N.H., K.K., C.H.B., I.M.M., S.M.T., M.G.D, J.A.E., Y.P.M. and A.C.F.; Formal analysis, J.H., C.C., E.M.S., A.D., K.V.F., M.G.C., K.K., H.H., B.E.W., I.M.M., S.M.T., C.H.B., J.A.E., Y.P.M. ,and A.C.F.; Investigation, J.H., C.C., R.S., T.L. E.M.S., N.U.P., A.D., K.V.F., M.T.H., C.T.J., D.A.R.H., J.T.F., M.L.B., R.J.M., C.Y., M.A.H., M.G.C., K.K., B.E.W., and A.C.F.; Resources, W.N., H.H.; Data Curation, E.M.S., M.J.G., U.M., and C.H.B.; Writing – Original Draft, J.H., C.C., R.S., K.K., M.G.D., C.H.B., J.A.E., Y.P.M., and A.C.F.; Writing – Revised Draft, J.H., C.C., M.G., K.K., Y.P.M., and A.C.F.; Visualization, J.H., C.C., and A.C.F.; Supervision, S.M.T, I.M.M., C.H.B., J.A.E., Y.P.M., and A.C.F.; Funding Acquisition, J.H. and A.C.F.

ACKNOWLEDGMENTS

This study was supported by a grant from The Rally Foundation for Childhood Cancer Research and The Truth 365 (A.C.F.); an Alex's Lemonade Stand Innovation grant (A.C.F. and Y.P.M.); a Wipe out Kids Cancer research grant (A.C.F.); a VCU Massey Cancer Center Pilot Project Application grant (A.C.F. and B.E.W.); and a grant from the Wellcome Trust (102696; C.H.B., M.J.G., and U.M.). A.C.F. is supported by the George and Lavina Blick Research Fund. J.A.E. is a consultant for AstraZeneca and has research agreements with AstraZeneca. We thank Dr. Sarah Conine for critical reading of the manuscript.

Received: June 18, 2015
Revised: November 14, 2015
Accepted: January 7, 2016
Published: February 8, 2016

REFERENCES

Barretina, J., Caponigro, G., Stransky, N., Venkatesan, K., Margolin, A.A., Kim, S., Wilson, C.J., Lehar, J., Kryukov, G.V., Sonkin, D., et al. (2012). The Cancer Cell Line Encyclopedia enables predictive modelling of anticancer drug sensitivity. Nature *483*, 603–607.

Brockmann, M., Poon, E., Berry, T., Carstensen, A., Deubzer, H.E., Rycak, L., Jamin, Y., Thway, K., Robinson, S.P., Roels, F., et al. (2013). Small molecule inhibitors of aurora-a induce proteasomal degradation of N-myc in childhood neuroblastoma. Cancer Cell *24*, 75–89.

Burkhart, C.A., Cheng, A.J., Madafiglio, J., Kavallaris, M., Mili, M., Marshall, G.M., Weiss, W.A., Khachigian, L.M., Norris, M.D., and Haber, M. (2003). Effects of MYCN antisense oligonucleotide administration on tumorigenesis in a murine model of neuroblastoma. J. Natl. Cancer Inst. *95*, 1394–1403.

Chen, L., Iraci, N., Gherardi, S., Gamble, L.D., Wood, K.M., Perini, G., Lunec, J., and Tweddle, D.A. (2010). p53 is a direct transcriptional target of MYCN in neuroblastoma. Cancer Res. *70*, 1377–1388.

Chipumuro, E., Marco, E., Christensen, C.L., Kwiatkowski, N., Zhang, T., Hatheway, C.M., Abraham, B.J., Sharma, B., Yeung, C., Altabef, A., et al. (2014). CDK7 inhibition suppresses super-enhancer-linked oncogenic transcription in MYCN-driven cancer. Cell *159*, 1126–1139.

Choudhary, G.S., Al-Harbi, S., Mazumder, S., Hill, B.T., Smith, M.R., Bodo, J., Hsi, E.D., and Almasan, A. (2015). MCL-1 and BCL-xL-dependent resistance to the BCL-2 inhibitor ABT-199 can be overcome by preventing PI3K/AKT/mTOR activation in lymphoid malignancies. Cell Death Dis. *6*, e1593.

Crystal, A.S., Shaw, A.T., Sequist, L.V., Friboulet, L., Niederst, M.J., Lockerman, E.L., Frias, R.L., Gainor, J.F., Amzallag, A., Greninger, P., et al. (2014). Patient-derived models of acquired resistance can identify effective drug combinations for cancer. Science *346*, 1480–1486.

Delehouze, C., Godl, K., Loaec, N., Bruyere, C., Desban, N., Oumata, N., Galons, H., Roumeliotis, T.I., Giannopoulou, E.G., Grenet, J., et al. (2014). CDK/CK1 inhibitors roscovitine and CR8 downregulate amplified MYCN in neuroblastoma cells. Oncogene *33*, 5675–5687.

Faber, A.C., Coffee, E.M., Costa, C., Dastur, A., Ebi, H., Hata, A.N., Yeo, A.T., Edelman, E.J., Song, Y., Tam, A.T., et al. (2014). mTOR inhibition specifically sensitizes colorectal cancers with KRAS or BRAF mutations to BCL-2/BCL-XL inhibition by suppressing MCL-1. Cancer Discov. *4*, 42–52.

Faber, A.C., Farago, A.F., Costa, C., Dastur, A., Gomez-Caraballo, M., Robbins, R., Wagner, B.L., Rideout, W.M., 3rd, Jakubik, C.T., Ham, J., et al. (2015). Assessment of ABT-263 activity across a cancer cell line collection leads to a potent combination therapy for small-cell lung cancer. Proc. Natl. Acad. Sci. USA *112*, E1288–E1296.

Faisal, A., Vaughan, L., Bavetsias, V., Sun, C., Atrash, B., Avery, S., Jamin, Y., Robinson, S.P., Workman, P., Blagg, J., et al. (2011). The aurora kinase inhibitor CCT137690 downregulates MYCN and sensitizes MYCN-amplified neuroblastoma in vivo. Mol. Cancer Ther. *10*, 2115–2123.

Fulda, S., Lutz, W., Schwab, M., and Debatin, K.M. (1999). MycN sensitizes neuroblastoma cells for drug-induced apoptosis. Oncogene *18*, 1479–1486.

Gao, R.N., Levy, I.G., Woods, W.G., Coombs, B.A., Gaudette, L.A., and Hill, G.B. (1997). Incidence and mortality of neuroblastoma in Canada compared with other childhood cancers. Cancer Causes Control *8*, 745–754.

Garnett, M.J., Edelman, E.J., Heidorn, S.J., Greenman, C.D., Dastur, A., Lau, K.W., Greninger, P., Thompson, I.R., Luo, X., Soares, J., et al. (2012). Systematic identification of genomic markers of drug sensitivity in cancer cells. Nature *483*, 570–575.

Goodman, C.A., Mabrey, D.M., Frey, J.W., Miu, M.H., Schmidt, E.K., Pierre, P., and Hornberger, T.A. (2011). Novel insights into the regulation of skeletal muscle protein synthesis as revealed by a new nonradioactive in vivo technique. FASEB J. *25*, 1028–1039.

Gustafson, W.C., Meyerowitz, J.G., Nekritz, E.A., Chen, J., Benes, C., Charron, E., Simonds, E.F., Seeger, R., Matthay, K.K., Hertz, N.T., et al. (2014). Drugging MYCN through an allosteric transition in Aurora kinase A. Cancer Cell *26*, 414–427.

Harley, M.E., Allan, L.A., Sanderson, H.S., and Clarke, P.R. (2010). Phosphorylation of Mcl-1 by CDK1-cyclin B1 initiates its Cdc20-dependent destruction during mitotic arrest. EMBO J. *29*, 2407–2420.

Haschka, M.D., Soratroi, C., Kirschnek, S., Hacker, G., Hilbe, R., Geley, S., Villunger, A., and Fava, L.L. (2015). The NOXA-MCL1-BIM axis defines lifespan on extended mitotic arrest. Nat. Commun. *6*, 6891.

Hata, A.N., Yeo, A., Faber, A.C., Lifshits, E., Chen, Z., Cheng, K.A., Walton, Z., Sarosiek, K.A., Letai, A., Heist, R.S., et al. (2014). Failure to induce apoptosis via BCL-2 family proteins underlies lack of efficacy of combined MEK and PI3K inhibitors for KRAS-mutant lung cancers. Cancer Res. *74*, 3146–3156.

Hata, A.N., Engelman, J.A., and Faber, A.C. (2015). The BCL2 family: key mediators of the apoptotic response to targeted anticancer therapeutics. Cancer Discov. *5*, 475–487.

Hogarty, M.D. (2003). The requirement for evasion of programmed cell death in neuroblastomas with MYCN amplification. Cancer Lett. *197*, 173–179.

Hsieh, A.C., Costa, M., Zollo, O., Davis, C., Feldman, M.E., Testa, J.R., Meyuhas, O., Shokat, K.M., and Ruggero, D. (2010). Genetic dissection of the oncogenic mTOR pathway reveals druggable addiction to translational control via 4EBP-eIF4E. Cancer Cell *17*, 249–261.

Huang, M., and Weiss, W.A. (2013). Neuroblastoma and MYCN. Cold Spring Harb. Perspect. Med. *3*, a014415.

Huang, M., Shen, A., Ding, J., and Geng, M. (2014). Molecularly targeted cancer therapy: some lessons from the past decade. Trends Pharmacol. Sci. *35*, 41–50.

Janoueix-Lerosey, I., Lequin, D., Brugieres, L., Ribeiro, A., de Pontual, L., Combaret, V., Raynal, V., Puisieux, A., Schleiermacher, G., Pierron, G., et al. (2008). Somatic and germline activating mutations of the ALK kinase receptor in neuroblastoma. Nature *455*, 967–970.

Jasty, R., van Golen, C., Lin, H.J., Solomon, G., Heidelberger, K., Polverini, P., Opipari, A., Feldman, E., and Castle, V.P. (2001). Bcl-2 and M-Myc coexpression increases IGF-IR and features of malignant growth in neuroblastoma cell lines. Neoplasia *3*, 304–313.

Kool, M., Koster, J., Bunt, J., Hasselt, N.E., Lakeman, A., van Sluis, P., Troost, D., Meeteren, N.S., Caron, H.N., Cloos, J., et al. (2008). Integrated genomics identifies five medulloblastoma subtypes with distinct genetic profiles, pathway signatures and clinicopathological features. PLoS One *3*, e3088.

Laude, M., Siessmann, K.L., Mokyr, M.B., and Dray, S. (1991). Advantages of adoptive chemoimmunotherapy with polyethylene glycol-cultured, antigen-activated, tumor-infiltrated spleen cells for the complete eradication of lethal MOPC-315 plasmacytomas. Cancer Res. *51*, 4516–4522.

Lucas, K.M., Mohana-Kumaran, N., Lau, D., Zhang, X.D., Hersey, P., Huang, D.C., Weninger, W., Haass, N.K., and Allen, J.D. (2012). Modulation of NOXA and MCL-1 as a strategy for sensitizing melanoma cells to the BH3-mimetic ABT-737. Clin. Cancer Res. *18*, 783–795.

Ma, S., Seymour, J.F., Lanasa, M.C., Kipps, T.J., Barrientos, J.C., Davids, M.S., Mason-Bright, T., Rudersdorf, N., Yang, J., Munasinghe, W., Zhu, M., et al. (2014). ABT-199 (GDC-0199) combined with rituximab (R) in patients (pts) with relapsed/refractory (R/R) chronic lymphocytic leukemia (CLL): interim results of a phase 1b study. J Clin Oncol ASCO Annu Meet Abstr *32* (May Suppl), abstract 7013.

Mallya, S., Fitch, B.A., Lee, J.S., So, L., Janes, M.R., and Fruman, D.A. (2014). Resistance to mTOR kinase inhibitors in lymphoma cells lacking 4EBP1. PLoS One *9*, e88865.

Manfredi, M.G., Ecsedy, J.A., Chakravarty, A., Silverman, L., Zhang, M., Hoar, K.M., Stroud, S.G., Chen, W., Shinde, V., Huck, J.J., et al. (2011). Characterization of Alisertib (MLN8237), an investigational small-molecule inhibitor of aurora A kinase using novel in vivo pharmacodynamic assays. Clin. Cancer Res. *17*, 7614–7624.

Maris, J.M., Morton, C.L., Gorlick, R., Kolb, E.A., Lock, R., Carol, H., Keir, S.T., Reynolds, C.P., Kang, M.H., Wu, J., et al. (2010). Initial testing of the aurora kinase A inhibitor MLN8237 by the Pediatric Preclinical Testing Program (PPTP). Pediatr. Blood Cancer *55*, 26–34.

Mason, K.D., Carpinelli, M.R., Fletcher, J.I., Collinge, J.E., Hilton, A.A., Ellis, S., Kelly, P.N., Ekert, P.G., Metcalf, D., Roberts, A.W., et al. (2007). Programmed anuclear cell death delimits platelet life span. Cell *128*, 1173–1186.

Maurer, U., Charvet, C., Wagman, A.S., Dejardin, E., and Green, D.R. (2006). Glycogen synthase kinase-3 regulates mitochondrial outer membrane permeabilization and apoptosis by destabilization of MCL-1. Mol. Cell *21*, 749–760.

McCormick, F. (2015). KRAS as a therapeutic target. Clin. Cancer Res. *21*, 1797–1801.

Mills, J.R., Hippo, Y., Robert, F., Chen, S.M., Malina, A., Lin, C.J., Trojahn, U., Wendel, H.G., Charest, A., Bronson, R.T., et al. (2008). mTORC1 promotes survival through translational control of Mcl-1. Proc. Natl. Acad. Sci. USA *105*, 10853–10858.

Mosse, Y.P., Lipsitz, E., Fox, E., Teachey, D.T., Maris, J.M., Weigel, B., Adamson, P.C., Ingle, M.A., Ahern, C.H., and Blaney, S.M. (2012). Pediatric phase I trial and pharmacokinetic study of MLN8237, an investigational oral selective small-molecule inhibitor of Aurora kinase A: a Children's Oncology Group Phase I Consortium study. Clin. Cancer Res. *18*, 6058–6064.

Murphy, D.M., Buckley, P.G., Bryan, K., Das, S., Alcock, L., Foley, N.H., Prenter, S., Bray, I., Watters, K.M., Higgins, D., and Stallings, R.L. (2009). Global MYCN transcription factor binding analysis in neuroblastoma reveals association with distinct E-box motifs and regions of DNA hypermethylation. PLoS One *4*, e8154.

Nakajima, W., Hicks, M.A., Tanaka, N., Krystal, G.W., and Harada, H. (2014). Noxa determines localization and stability of MCL-1 and consequently ABT-737 sensitivity in small cell lung cancer. Cell Death Dis. *5*, e1052.

Nalluri, S., Peirce, S.K., Tanos, R., Abdella, H.A., Karmali, D., Hogarty, M.D., and Goldsmith, K.C. (2015). EGFR signaling defines Mcl(-)1 survival dependency in neuroblastoma. Cancer Biol. Ther. *16*, 276–286.

Nikiforov, M.A., Riblett, M., Tang, W.H., Gratchouck, V., Zhuang, D., Fernandez, Y., Verhaegen, M., Varambally, S., Chinnaiyan, A.M., Jakubowiak, A.J., and Soengas, M.S. (2007). Tumor cell-selective regulation of NOXA by c-MYC in response to proteasome inhibition. Proc. Natl. Acad. Sci. USA *104*, 19488–19493.

Northcott, P.A., Shih, D.J., Peacock, J., Garzia, L., Morrissy, A.S., Zichner, T., Stutz, A.M., Korshunov, A., Reimand, J., Schumacher, S.E., et al. (2012). Subgroup-specific structural variation across 1,000 medulloblastoma genomes. Nature *488*, 49–56.

Petroni, M., Veschi, V., Prodosmo, A., Rinaldo, C., Massimi, I., Carbonari, M., Dominici, C., McDowell, H.P., Rinaldi, C., Screpanti, I., et al. (2011). MYCN sensitizes human neuroblastoma to apoptosis by HIPK2 activation through a DNA damage response. Mol. Cancer Res. *9*, 67–77.

Puissant, A., Frumm, S.M., Alexe, G., Bassil, C.F., Qi, J., Chanthery, Y.H., Nekritz, E.A., Zeid, R., Gustafson, W.C., Greninger, P., et al. (2013). Targeting MYCN in neuroblastoma by BET bromodomain inhibition. Cancer Discov. *3*, 308–323.

Qing, G., Li, B., Vu, A., Skuli, N., Walton, Z.E., Liu, X., Mayes, P.A., Wise, D.R., Thompson, C.B., Maris, J.M., et al. (2012). ATF4 regulates MYC-mediated neuroblastoma cell death upon glutamine deprivation. Cancer Cell *22*, 631–644.

Roberts, A.W., Seymour, J.F., Brown, J.R., Wierda, W.G., Kipps, T.J., Khaw, S.L., Carney, D.A., He, S.Z., Huang, D.C., Xiong, H., et al. (2012). Substantial susceptibility of chronic lymphocytic leukemia to BCL2 inhibition: results of a phase I study of navitoclax in patients with relapsed or refractory disease. J. Clin. Oncol. *30*, 488–496.

Robinson, G., Parker, M., Kranenburg, T.A., Lu, C., Chen, X., Ding, L., Phoenix, T.N., Hedlund, E., Wei, L., Zhu, X., et al. (2012). Novel mutations target distinct subgroups of medulloblastoma. Nature *488*, 43–48.

Ryan, S.L., Schwalbe, E.C., Cole, M., Lu, Y., Lusher, M.E., Megahed, H., O'Toole, K., Nicholson, S.L., Bognar, L., Garami, M., et al. (2012). MYC family amplification and clinical risk-factors interact to predict an extremely poor prognosis in childhood medulloblastoma. Acta Neuropathol. *123*, 501–513.

Schatz, J.H., Oricchio, E., Wolfe, A.L., Jiang, M., Linkov, I., Maragulia, J., Shi, W., Zhang, Z., Rajasekhar, V.K., Pagano, N.C., et al. (2011). Targeting cap-dependent translation blocks converging survival signals by AKT and PIM kinases in lymphoma. J. Exp. Med. *208*, 1799–1807.

Seymour, J.F., Davids, M.S., Pagel, J.M., Kahl, B.S., Wierda, W.G., Puvvada, S., Gerecitano, J.F., Kipps, T.J., Anderson, M.A., Huang, D.C.S., et al. (2014). ABT-199 (GDC-0199) in relapsed/refractory (R/R) chronic lymphocytic leukemia (CLL) and small lymphocytic lymphoma (SLL): high complete- response rate and durable disease control. J Clin Oncol ASCO Annu Meet Abstr *32* (May Suppl), abstract 7015.

Shohet, J.M., Ghosh, R., Coarfa, C., Ludwig, A., Benham, A.L., Chen, Z., Patterson, D.M., Barbieri, E., Mestdagh, P., Sikorski, D.N., et al. (2011). A genome-wide search for promoters that respond to increased MYCN reveals both new oncogenic and tumor suppressor microRNAs associated with aggressive neuroblastoma. Cancer Res. *71*, 3841–3851.

Slack, A., Chen, Z., Tonelli, R., Pule, M., Hunt, L., Pession, A., and Shohet, J.M. (2005). The p53 regulatory gene MDM2 is a direct transcriptional target of MYCN in neuroblastoma. Proc. Natl. Acad. Sci. USA *102*, 731–736.

Souers, A.J., Leverson, J.D., Boghaert, E.R., Ackler, S.L., Catron, N.D., Chen, J., Dayton, B.D., Ding, H., Enschede, S.H., Fairbrother, W.J., et al. (2013). ABT-199, a potent and selective BCL-2 inhibitor, achieves antitumor activity while sparing platelets. Nat. Med. *19*, 202–208.

Sunaga, N., Shames, D.S., Girard, L., Peyton, M., Larsen, J.E., Imai, H., Soh, J., Sato, M., Yanagitani, N., Kaira, K., et al. (2011). Knockdown of oncogenic KRAS in non-small cell lung cancers suppresses tumor growth and sensitizes tumor cells to targeted therapy. Mol. Cancer Ther. *10*, 336–346.

Teitz, T., Inoue, M., Valentine, M.B., Zhu, K., Rehg, J.E., Zhao, W., Finkelstein, D., Wang, Y.D., Johnson, M.D., Calabrese, C., et al. (2013). Th-MYCN mice with caspase-8 deficiency develop advanced neuroblastoma with bone marrow metastasis. Cancer Res. *73*, 4086–4097.

Ushmorov, A., Hogarty, M.D., Liu, X., Knauss, H., Debatin, K.M., and Beltinger, C. (2008). N-myc augments death and attenuates protective effects of Bcl-2 in trophically stressed neuroblastoma cells. Oncogene *27*, 3424–3434.

Veschi, V., Petroni, M., Cardinali, B., Dominici, C., Screpanti, I., Frati, L., Bartolazzi, A., Gulino, A., and Giannini, G. (2012). Galectin-3 impairment of MYCN-dependent apoptosis-sensitive phenotype is antagonized by nutlin-3 in neuroblastoma cells. PLoS One *7*, e49139.

Wang, Q., Diskin, S., Rappaport, E., Attiyeh, E., Mosse, Y., Shue, D., Seiser, E., Jagannathan, J., Shusterman, S., Bansal, M., et al. (2006). Integrative genomics identifies distinct molecular classes of neuroblastoma and shows that multiple genes are targeted by regional alterations in DNA copy number. Cancer Res. *66*, 6050–6062.

Wang, X., Gu, Z., Li, G., Zhang, S., Cao, Z., Yang, Z., and Liu, G. (2014). Norcantharidin enhances ABT-263-mediated anticancer activity in neuroblastoma cells by upregulation of Noxa. Oncol. Rep. *32*, 716–722.

Weiss, W.A., Aldape, K., Mohapatra, G., Feuerstein, B.G., and Bishop, J.M. (1997). Targeted expression of MYCN causes neuroblastoma in transgenic mice. EMBO J. *16*, 2985–2995.

Wertz, I.E., Kusam, S., Lam, C., Okamoto, T., Sandoval, W., Anderson, D.J., Helgason, E., Ernst, J.A., Eby, M., Liu, J., et al. (2011). Sensitivity to antitubulin chemotherapeutics is regulated by MCL1 and FBW7. Nature *471*, 110–114.

Yang, W., Soares, J., Greninger, P., Edelman, E.J., Lightfoot, H., Forbes, S., Bindal, N., Beare, D., Smith, J.A., Thompson, I.R., et al. (2013). Genomics of drug sensitivity in Cancer (GDSC): a resource for therapeutic biomarker discovery in cancer cells. Nucleic Acids Res. *41*, D955–D961.

Zorde Khvalevsky, E., Gabai, R., Rachmut, I.H., Horwitz, E., Brunschwig, Z., Orbach, A., Shemi, A., Golan, T., Domb, A.J., Yavin, E., et al. (2013). Mutant KRAS is a druggable target for pancreatic cancer. Proc. Natl. Acad. Sci. USA *110*, 20723–20728.

Cancer Cell, Volume *29*

Supplemental Information

Exploitation of the Apoptosis-Primed State of *MYCN*-Amplified Neuroblastoma to Develop a Potent and Specific Targeted Therapy Combination

Jungoh Ham, Carlotta Costa, Renata Sano, Timothy L. Lochmann, Erin M. Sennott, Neha U. Patel, Anahita Dastur, Maria Gomez-Caraballo, Kateryna Krytska, Aaron N. Hata, Konstantinos V. Floros, Mark T. Hughes, Charles T. Jakubik, Daniel A.R. Heisey, Justin T. Ferrell, Molly L. Bristol, Ryan J. March, Craig Yates, Mark A. Hicks, Wataru Nakajima, Madhu Gowda, Brad E. Windle, Mikhail G. Dozmorov, Mathew J. Garnett, Ultan McDermott, Hisashi Harada, Shirley M. Taylor, Iain M. Morgan, Cyril H. Benes, Jeffrey A. Engelman, Yael P. Mossé, and Anthony C. Faber

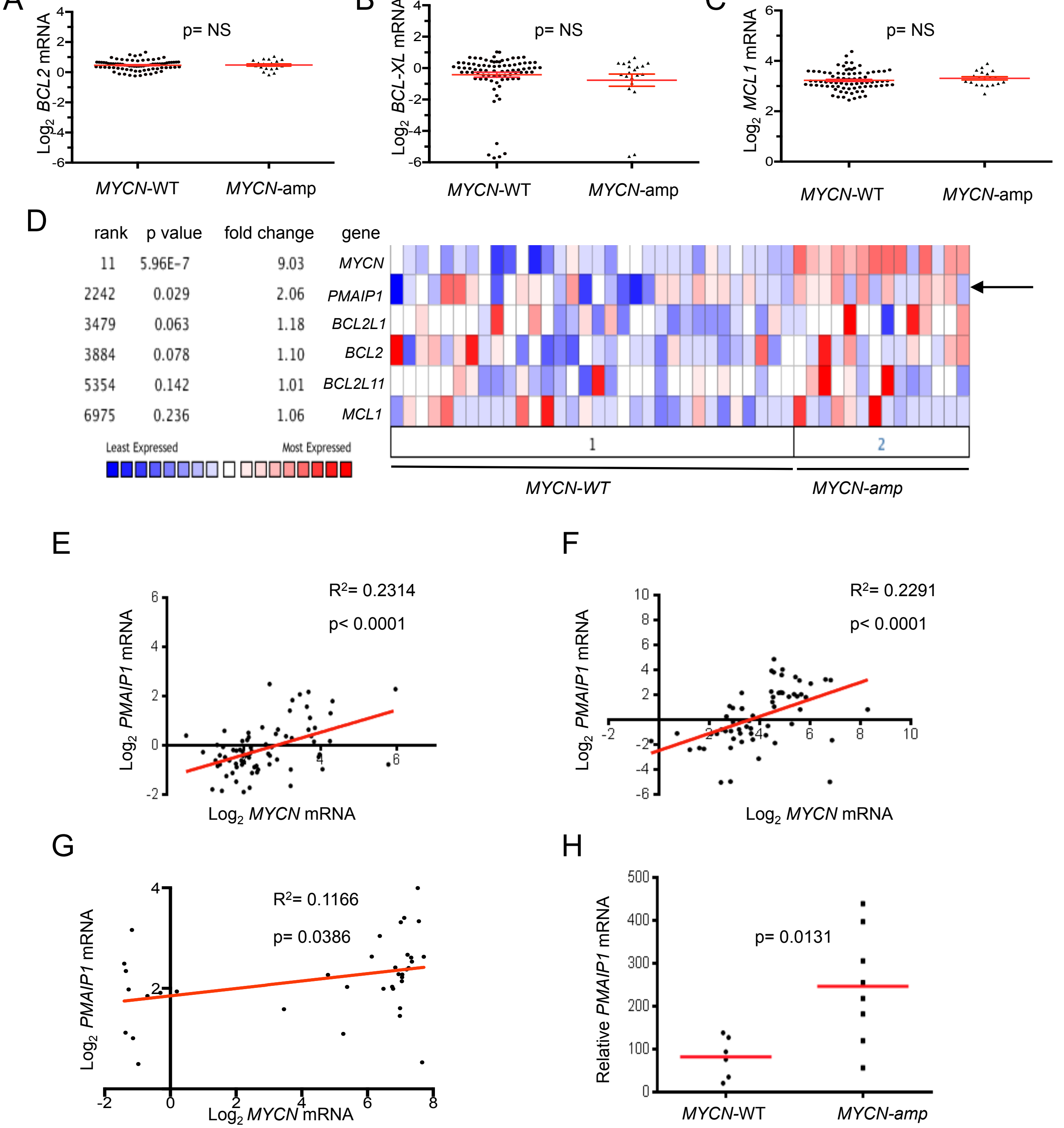

Figure S1 (related to Figure 1) Gene expression data parsing reveal *MYCN*-amplified neuroblastoma have high NOXA expression.

(A-C) RNA expression from the Wang dataset (Wang et al., 2006) for *MYCN*-WT neuroblastoma tumors (n=81) and *MYCN*-amplified (amp) neuroblastoma tumors (n=20). NS= not significant by non-parametric Mann-Whitney U-Test. Red lines are mean, error bars are -/+S.E.M. Y axis = Log_2 median-centered.

(D) Comparison of BCL-2 family members in *MYCN* wild-type (WT) and *MYCN*-amplified (amp) neuroblastomas (Janoueix-Lerosey et al., 2008) The Oncomine™ Platform (Life Technologies, Ann Arbor, MI) was used for analysis and visualization. Arrow indicates NOXA (*PMAIP1*). Data is Log_2 median-centered.

(E, F*)* Linear regression analysis between *MYCN* and NOXA (*PMAIP1*) expression in two medulloblastoma tumor sets (Kool et al., 2008; Robinson et al., 2012) (R^2=0.2314 for Robinson et al. (E), R^2=0.2291 for Kool et al. (F), p value <0.0001 for both sets). Data from Oncomine and is Log_2 median-centered.

(G) Linear regression analysis between *MYCN* and NOXA (*PMAIP1*) expression in neuroblastoma cell lines (Garnett et al. 2012). R^2=0.1166, p value = 0.0386. Data from Oncomine and is Log_2 median-centered.

(H) *MYCN*-WT and *MYCN*-amp neuroblastoma cell lines used in this study were assayed for NOXA RNA expression by qRTPCR. Student t-test indicated statistical difference between the two groups (p =0.0131).

Table S1 (related to Figure 1) *MYCN* amplified cancer cells are sensitive to ABT-263 (modified from cancerRxgene.org)

Drug Name	gene	sample (n)	mutant (n)	p value	IC50 Effect	Slope Effect	Mean (WT)	Mean (mutant)	Q Value
ABT-263	MYCN	661	26	0.000204583	0.064853587	0.0916665	2.28374	-0.105377	0.0112351
IPA-3	MYCN	665	25	0.235236	0.278877836	0.030481	5.52888	4.60757	0.712446
Vorinostat	MYCN	663	25	0.00558859	0.281225048	0.0885589	1.14141	0.0922058	0.115972
VX-680	MYCN	355	25	0.649953	0.293156819	-0.0351276	2.35922	2.31374	0.934278
OSI-906	MYCN	665	25	0.00418768	0.353332837	-0.0331439	3.44913	1.98539	0.0977126
ZM-447439	MYCN	642	26	0.000107534	0.353637246	0.0599163	2.97674	1.68667	0.0069408
QS11	MYCN	665	25	0.290149	0.362438229	0.0213664	3.96269	3.68273	0.751846
Methotrexate	MYCN	663	25	0.249704	0.425972929	-0.00741431	0.483483	0.65277	0.723843
OSU-03012	MYCN	665	25	0.228711	0.439066194	-0.0383004	3.13261	3.45487	0.706899
BAY 61-3606	MYCN	665	25	0.605774	0.440129015	0.029318	2.65854	2.4318	0.91608
PAC-1	MYCN	665	25	0.30013	0.452918437	-0.00240477	3.46285	3.11705	0.755648
AICAR	MYCN	663	25	0.0386602	0.476345427	-0.0352158	8.21457	8.42227	0.335325
GSK-650394	MYCN	665	25	0.486651	0.481901191	-0.0154645	4.26113	4.22894	0.868459
Mitomycin C	MYCN	665	25	0.335194	0.534143564	0.0123004	-1.10889	-1.02453	0.78655
CEP-701	MYCN	663	25	0.0117301	0.551063949	0.137619	-0.275621	-0.873251	0.18077
Axitinib	MYCN	663	25	0.0628123	0.560340483	0.0337669	3.09824	2.45822	0.421292
PF-562271	MYCN	672	26	0.0266798	0.561221112	0.102367	2.88291	2.70906	0.283028
AZD7762	MYCN	663	25	0.00921422	0.573696235	0.117877	-0.245575	-0.792295	0.1555
PD-0332991	MYCN	633	26	0.508188	0.579861133	0.0239354	2.69056	2.34934	0.878808
JNK Inhibitor VIII	MYCN	661	26	0.028968	0.605817759	-0.00732601	5.99778	5.57067	0.296237
BX-795	MYCN	659	26	0.244597	0.625227397	0.0128616	2.35212	1.60907	0.718255
Imatinib	MYCN	359	25	0.271146	0.626668695	-0.0290436	4.83213	4.70821	0.738206
Camptothecin	MYCN	663	25	0.432739	0.645238689	0.025919	-4.01124	-4.64046	0.842426
Epothilone B	MYCN	665	25	0.368465	0.685054621	0.0202401	-4.7685	-4.44229	0.810118
NSC-87877	MYCN	672	26	0.574995	0.70563189	-0.00738962	6.60198	6.60167	0.903914
LAQ824	MYCN	665	25	0.757507	0.711528008	0.0475988	-2.7742	-2.68253	0.971689
BMS-754807	MYCN	665	25	0.0591397	0.72414544	-0.0214165	1.24843	0.43582	0.40738
Parthenolide	MYCN	353	24	0.200715	0.741282248	0.0376962	4.83948	4.55609	0.669949
PHA-665752	MYCN	358	25	0.0068125	0.747175117	-0.0606826	4.77189	4.76723	0.133038
EHT 1864	MYCN	655	24	0.615758	0.759135673	0.0300669	4.6266	4.54701	0.920258
Thapsigargin	MYCN	665	25	0.00000127	0.776902287	0.199592	-4.03493	-3.99713	0.0002071
GW 441756	MYCN	663	25	0.566113	0.780078665	-0.00722484	4.04568	3.80271	0.903527
Nutlin-3a	MYCN	661	26	0.000000813	0.782352365	0.0914537	4.64933	4.0434	0.0001393
Sunitinib	MYCN	355	25	0.458238	0.788156025	0.00807471	3.11189	3.90644	0.858279
GSK269962A	MYCN	353	24	0.150767	0.792672515	0.0309754	3.44376	3.00397	0.602099
SB590885	MYCN	641	26	0.975958	0.832869196	-0.00945431	5.03294	5.51198	1.01848
Etoposide	MYCN	672	26	0.659007	0.841906379	0.00970601	1.8599	2.10136	0.937665
Lenalidomide	MYCN	663	25	0.459988	0.843482351	0.00221078	5.42334	5.49222	0.858644
LFM-A13	MYCN	665	25	0.145132	0.858300173	0.012893	6.22928	6.12854	0.595136

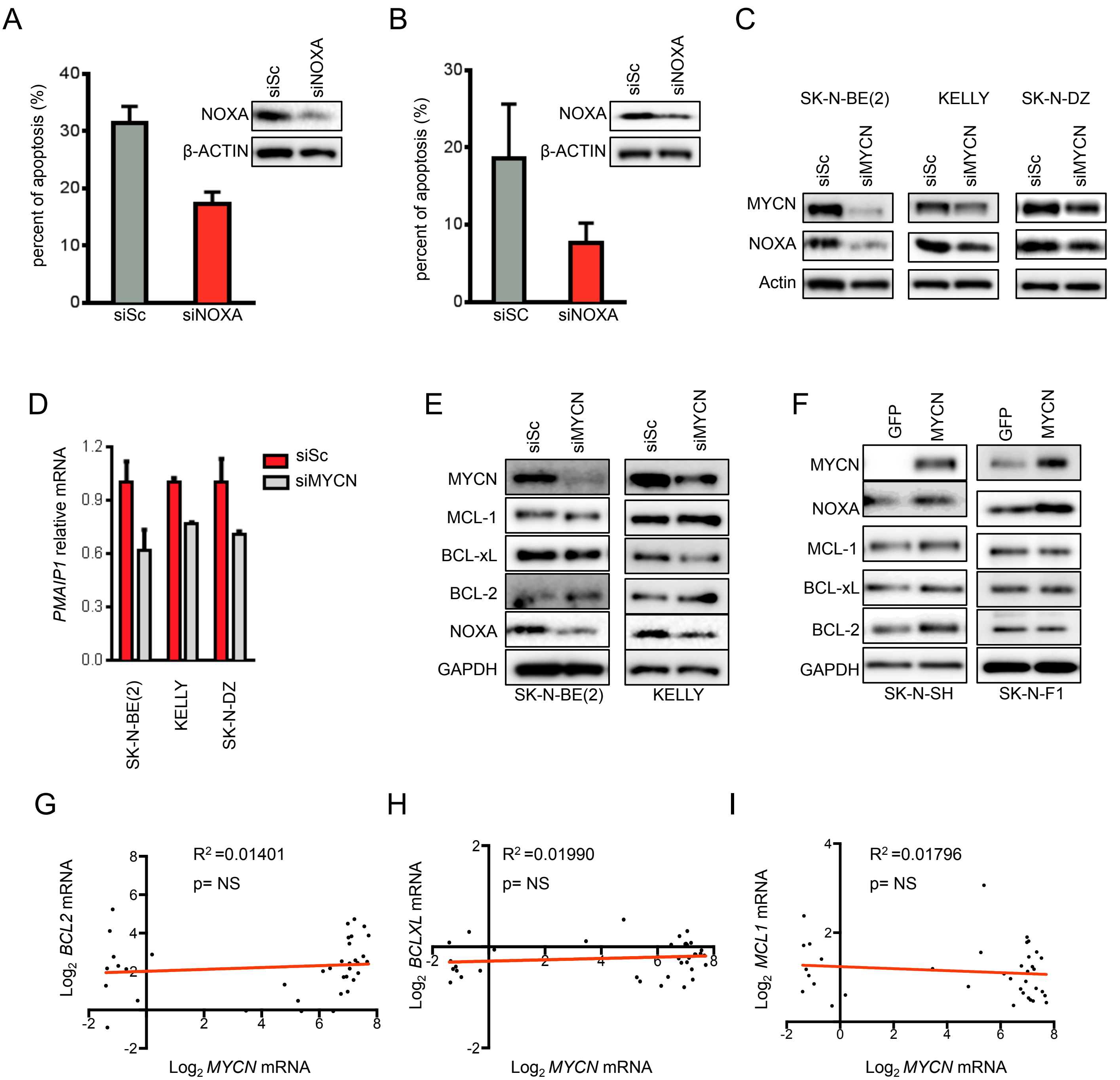

Figure S2 (related to Figure 2). NOXA is involved in *MYCN*-amplified neuroblastoma cell hypersensitivity to ABT-263.

(A, B) (A) SK-N-DZ and (B) SK-N-BE(2) *MYCN*-amplified neuroblastoma cells transfected with short interfering (si)-scrambed (Sc) or siNOXA were subsequently treated for 72 hr with ABT-263 and assayed for apoptosis or (inset) lysed to confirm knockdown. Error bars are +S.E.M.

(C, D) SK-N-BE(2), KELLY and SK-N-DZ cells were transfected with short interfering (si)-scrambed (Sc) or siMYCN and lysed to detect MYCN, NOXA or β-Actin protein (C) or mRNA levels of NOXA relative to β-Actin (D) was determined. Error bars are +S.D. for (D).

(E) SK-N-BE(2) and KELLY cells were transfected with short interfering (si)-scrambed (Sc) or siMYCN and lysed to detect the indicated antibodies.

(F) *MYCN*-wildtype neuroblastoma SK-N-SH and SK-N-F1 cells were engineered to express GFP or MYCN and the lysates were probed with the indicated antibodies.

(G-I) RNA expression levels of BCL-2 family members in Garnett et al (2012). p values were not significant (NS) for the linear regression analysis. Data from Oncomine and is Log_2 median-centered.

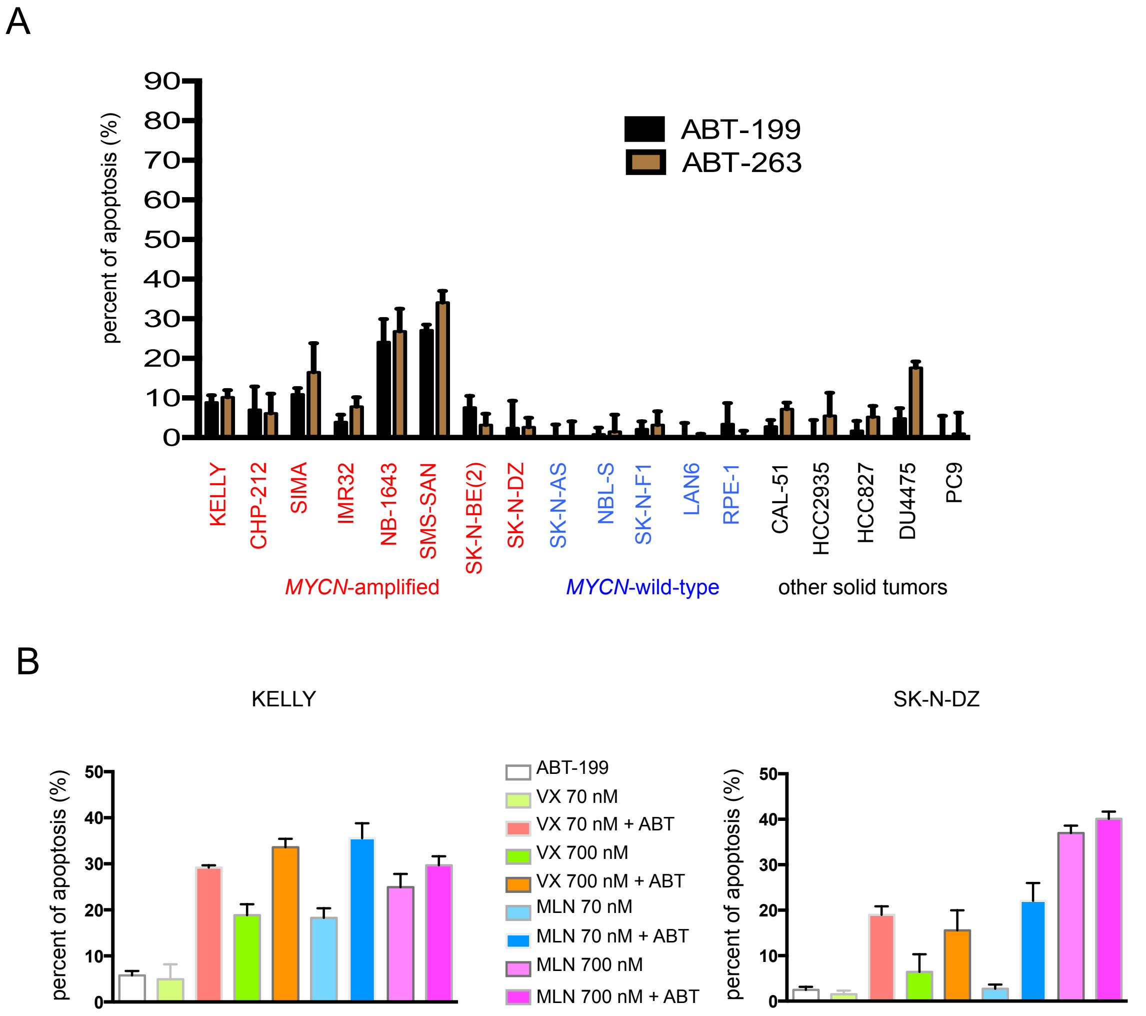

C

KELLY

MLN8237 (nM)

ABT199 (µM)	800	400	200	100	50	25	12.5
10	-0.5	-0.3	-0.2	-0.1	-0.2	-0.3	-0.1
5	1.5	3.2	3.8	13.1	7.4	9.1	13.3
2.5	5.0	9.0	13.3	33.1	19.7	20.7	18.4
1.25	4.6	8.4	11.5	29.2	17.1	15.1	10.1
0.63	5.2	8.3	11.5	25.4	17.3	15.9	3.5
0.32	5.1	8.4	12.0	26.8	17.0	18.2	14.2
0.16	5.0	9.0	12.2	27.6	17.9	20.6	9.3

SK-N-DZ

MLN8237 (nM)

ABT199 (µM)	800	400	200	100	50	25	12.5
10	-1.5	-1.8	-2.9	0.0	4.0	1.6	-1.2
5	5.4	7.3	7.9	5.3	46.6	44.1	12.5
2.5	1.4	4.1	2.4	8.1	34.2	34.6	14.4
1.25	1.6	0.6	1.4	7.3	25.5	20.3	12.2
0.63	1.6	3.0	1.8	4.9	24.0	12.1	7.6
0.32	3.0	7.6	5.5	9.4	23.7	13.1	5.9
0.16	2.5	2.7	3.0	5.1	19.4	0.6	7.0

Figure S3 (related to Figure 3 and 4). Synergy of ABT-199 and Aurora A inhibitors at low concentrations.
(A) The indicated cell lines were treated as in Figure 3B, for a duration of 24 hr and apoptosis was detected by FACS. Error bars are +S.D., n=3.
(B) Apoptosis as determined by FACS following 24 hr of indicated drug treatments in KELLY and SK-N-DZ cells. Error bars are +S.D., n=3.
(C) A dose matrix of MLN8237 and ABT-199 was created in KELLY and SK-N-DZ cells. Viability was assessed after 48 hr. Percent of the excess over the Bliss at each dose of the drug is presented.

Table S2 related to Figure 4. Drugs used in the apoptosis “anchor” screen

**Drug**	**Other Name**	**Target(s)**
Afatinib	BIBW2992	EGFR, HER2
Crizotinib	PF-2341066, Xalkori	MET, ALK
Gefitinib	ZD1839, Iressa	EGFR
Lapatinib	Tykerb, GW572016	ERBB2
AZD6244	Selumetinib, ARRY- 142886	MEK1/2
MK2206		AKT
BYL-719	Alpelisib	PI3K alpha
GDC-0941	Pictilisib	PI3K alpha/beta
PKC412	Midostaurin	PKC, VEGFR, PDGFR, FLT3, KIT
PF299804	Dacomitinib	EGFR, ERBB2
MLN8237	Alisertib	AURKA
VX680	Tozasertib, MK-0457	FLT3, Aurora kinases
AP26113	Brigatinib	ALK, EGFR
WZ4002		EGFR L858R /T790M
Dasatinib	BMS-354825, Sprycel	SRC, ABL, EPHB, SFK
Dabrafenib	GSK2118436	BRAF, CRAF
Imatinib	Gleevac, STI0571, CGP 57148	KIT, PDGFR, ABL
MP-470	Amuvatinib	KIT, PDGFR, FLT3
XL880	Foretinib, GSK1363089	MET, KDR, VEGFR, FLT4,
GSK429286A	KIN001-155	Rho kinase
Vorinostat	Zolinza, SAHA	HDAC inhibitor (Class I, Iia, Iib, IV)
NVP-BGJ398		FGFR
CT99021	CHIR-99021	GSK3
GDC-0449	Vismodegib	Hedgehog pathway

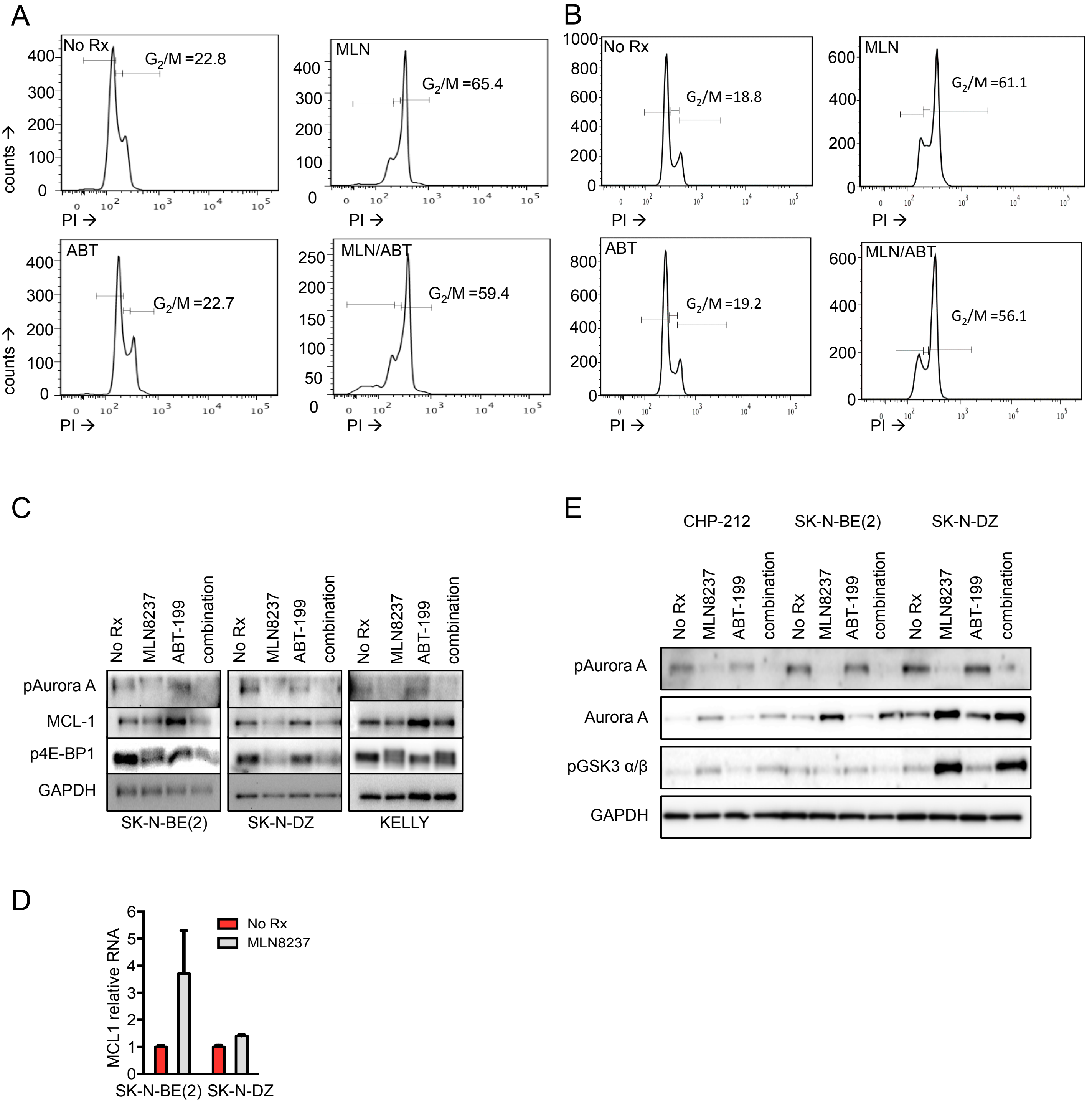

Figure S4 (related to Figure 5). Mitotic Arrest following MLN8237 treatment.
(A, B) Cell cycle profiles of *MYCN*-amplified KELLY (A) and SIMA (B) cells 24 hr following no treatment (No Rx), MLN8237 (MLN) treatment, ABT-199 (ABT) treatment, and combination treatment (MLN/ABT).
(C) Lysates from the indicated *MYCN*-amplified neuroblastoma cell lines were probed with the indicated antibodies following 16 hours of treatment with the indicated drugs.
(**D**) Relative MCL-1 RNA levels following no drug (No Rx) or MLN8237 24 hr after treatment in SK-N-BE(2) and SK-N-DZ. Error bars are +S.D.
(E) Lysates from the indicated *MYCN*-amplified neuroblastoma cell lines were probed with the indicated antibodies following 24 hr of treatment with the indicated drugs. Lysates from **(E)** were overlapping with those in main Figure 5B.

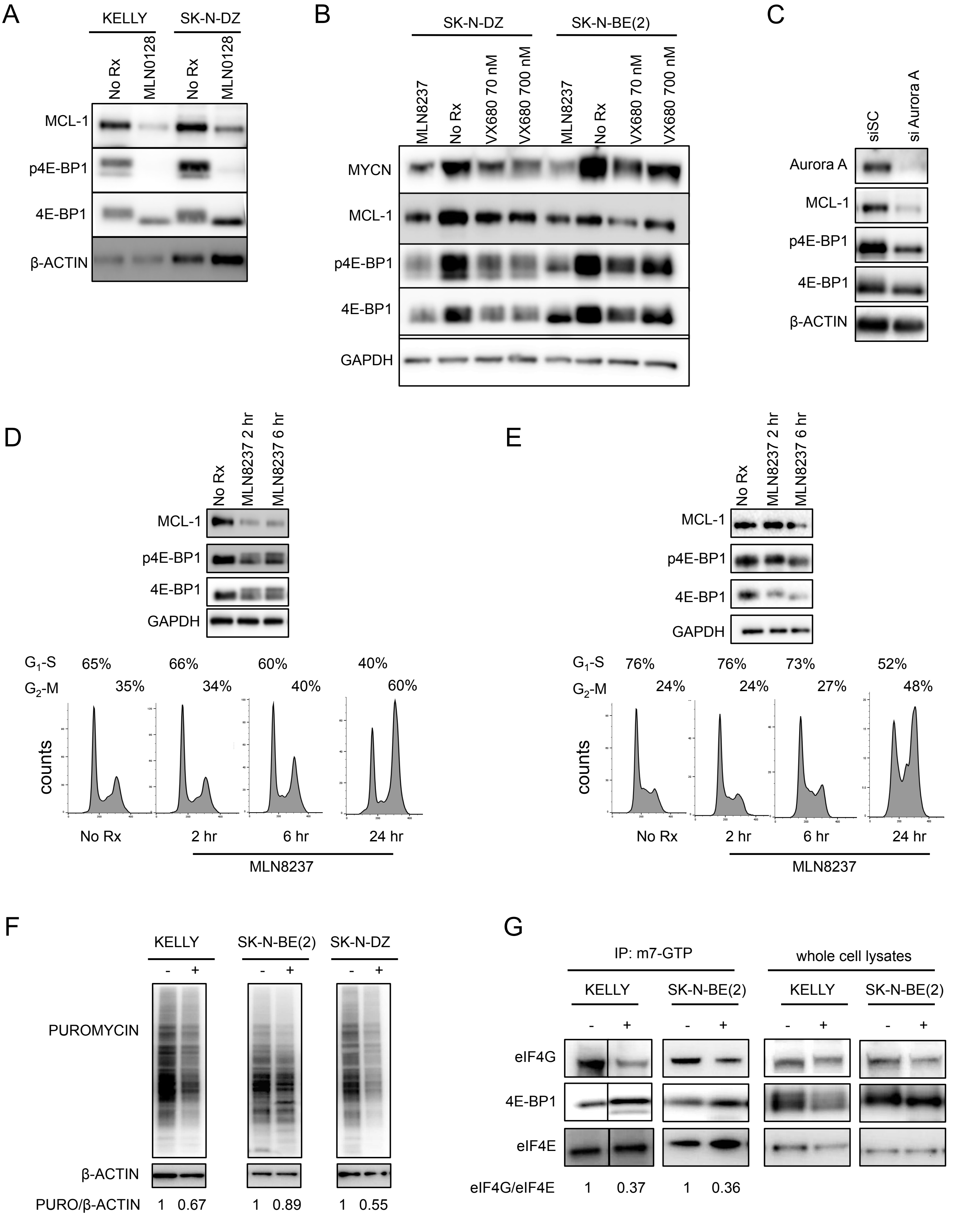

A
KELLY
SK-N-DZ
No Rx
MLN0128
No Rx
MLN0128
MCL-1
p4E-BP1
4E-BP1
β-ACTIN
B
SK-N-DZ
SK-N-BE(2)
MLN8237
No Rx
VX680 70 nM
VX680 700 nM
MLN8237
No Rx
VX680 70 nM
VX680 700 nM
MYCN
MCL-1
p4E-BP1
4E-BP1
GAPDH
C
siSC
si Aurora A
Aurora A
MCL-1
p4E-BP1
4E-BP1
β-ACTIN
D
No Rx
MLN8237 2 hr
MLN8237 6 hr
MCL-1
p4E-BP1
4E-BP1
GAPDH
G1-S 65% 66% 60% 40%
G2-M 35% 34% 40% 60%
counts
No Rx
2 hr
6 hr
24 hr
MLN8237
E
No Rx
MLN8237 2 hr
MLN8237 6 hr
MCL-1
p4E-BP1
4E-BP1
GAPDH
G1-S 76% 76% 73% 52%
G2-M 24% 24% 27% 48%
counts
No Rx
2 hr
6 hr
24 hr
MLN8237
F
KELLY
SK-N-BE(2)
SK-N-DZ
- + - + - +
PUROMYCIN
β-ACTIN
PURO/β-ACTIN 1 0.67 1 0.89 1 0.55
G
IP: m7-GTP
whole cell lysates
KELLY
SK-N-BE(2)
KELLY
SK-N-BE(2)
- + - + - + - +
eIF4G
4E-BP1
eIF4E
eIF4G/eIF4E 1 0.37 1 0.36

Figure S5 (related to Figure 5) MLN8237 downregulates MCL-1 through inhibition of cap-dependent protein translation.
(A) KELLY and SK-N-DZ cells were untreated (No Rx) or treated with100 nM MLN0128 for 6 hr and lysates were probed with the indicated antibodies.
(B) *MYCN*-amplified cell lines, SK-N-DZ and SK-N-BE(2), were treated with the indicated drugs at the indicated concentrations for 24 hr and probed with the indicated antibodies.
(C) SK-N-DZ cells were treated with 50 nM control (siSC) or 50 nM siAurora A siRNA for 24 hr and lysates probed with the indicated antibodies.
(D, E) Top: SK-N-BE(2) (D) and KELLY (E) cells were left untreated (no Rx) or treated with 100 nM MLN8237 for 2 hr or 6 hr, and lysates were probed with the indicated antibodies. Bottom: Cell cycle analysis of SK-N-BE(2) and KELLY cells at the indicated times following treatment with 100 nM MLN8237.
(F) *MYCN*-amplified neuroblastoma cells were left treated with DMSO (-) or 100 nM MLN8237 (+), followed by 1 μM of puromycin exposure and lysates were subjected to Western blot analysis with the indicated antibodies.
(G) Cells were treated with DMSO (-) or 100 nM MLN8237 (+) and lysates were subjected to m^7GTP pull-downs, and analyzed for levels of the indicated proteins. Bands were quantified and the ratio of eIF4G:eIF4E was presented. Immunoprecipiated lysates from KELLY cells were run on the same gel but in discontigious lanes.

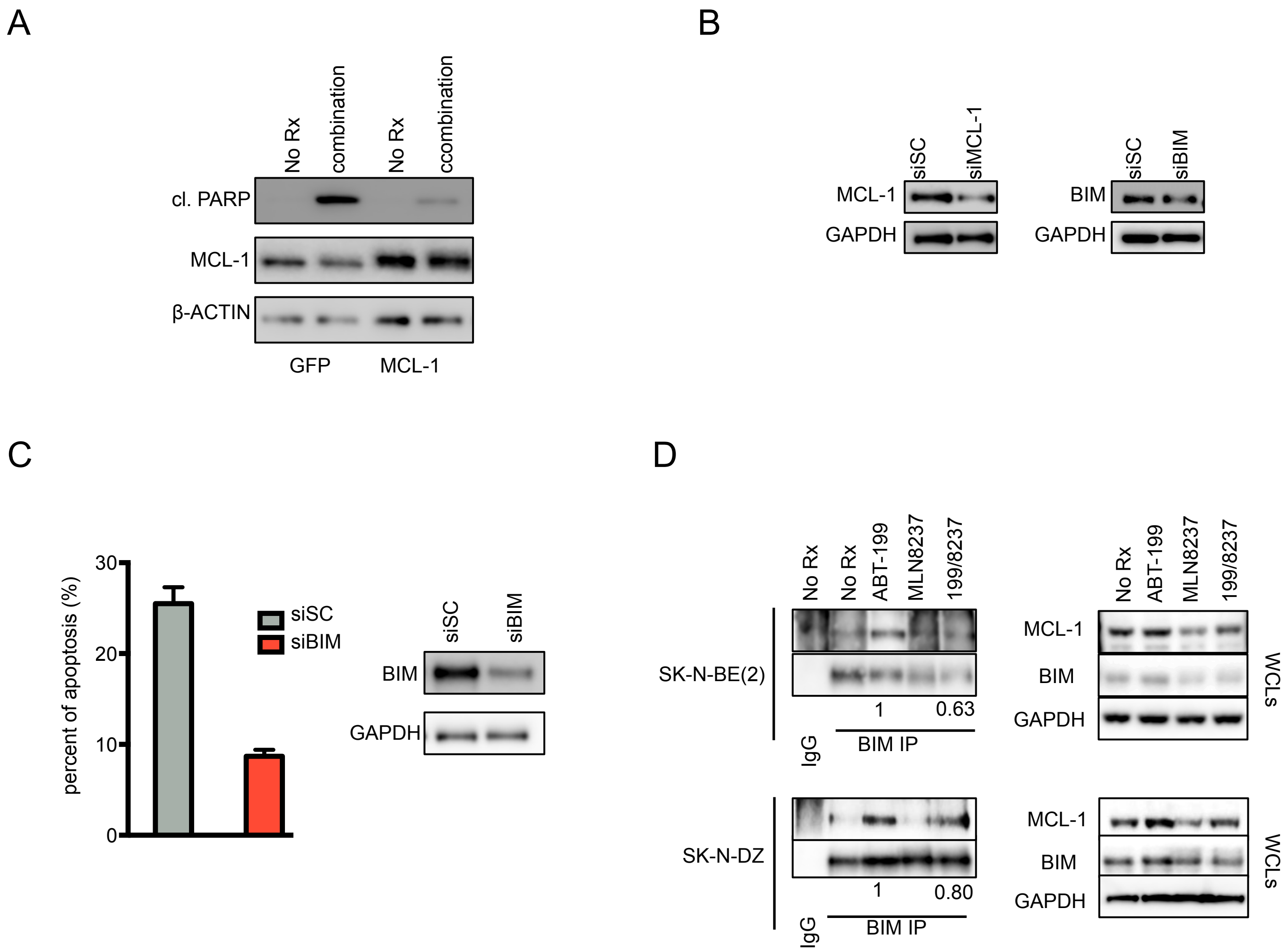
A
No Rx
combination
No Rx
ccombination
cl. PARP
MCL-1
β-ACTIN
GFP
MCL-1
B
siSC
siMCL-1
MCL-1
GAPDH
siSC
siBIM
BIM
GAPDH
C
percent of apoptosis (%)
30
20
10
0
siSC
siBIM
siSC
siBIM
BIM
GAPDH
D
No Rx
No Rx
ABT-199
MLN8237
199/8237
SK-N-BE(2)
1
0.63
IgG
BIM IP
No Rx
ABT-199
MLN8237
199/8237
MCL-1
BIM
GAPDH
WCLs
SK-N-DZ
1
0.80
IgG
BIM IP
MCL-1
BIM
GAPDH
WCLs

E

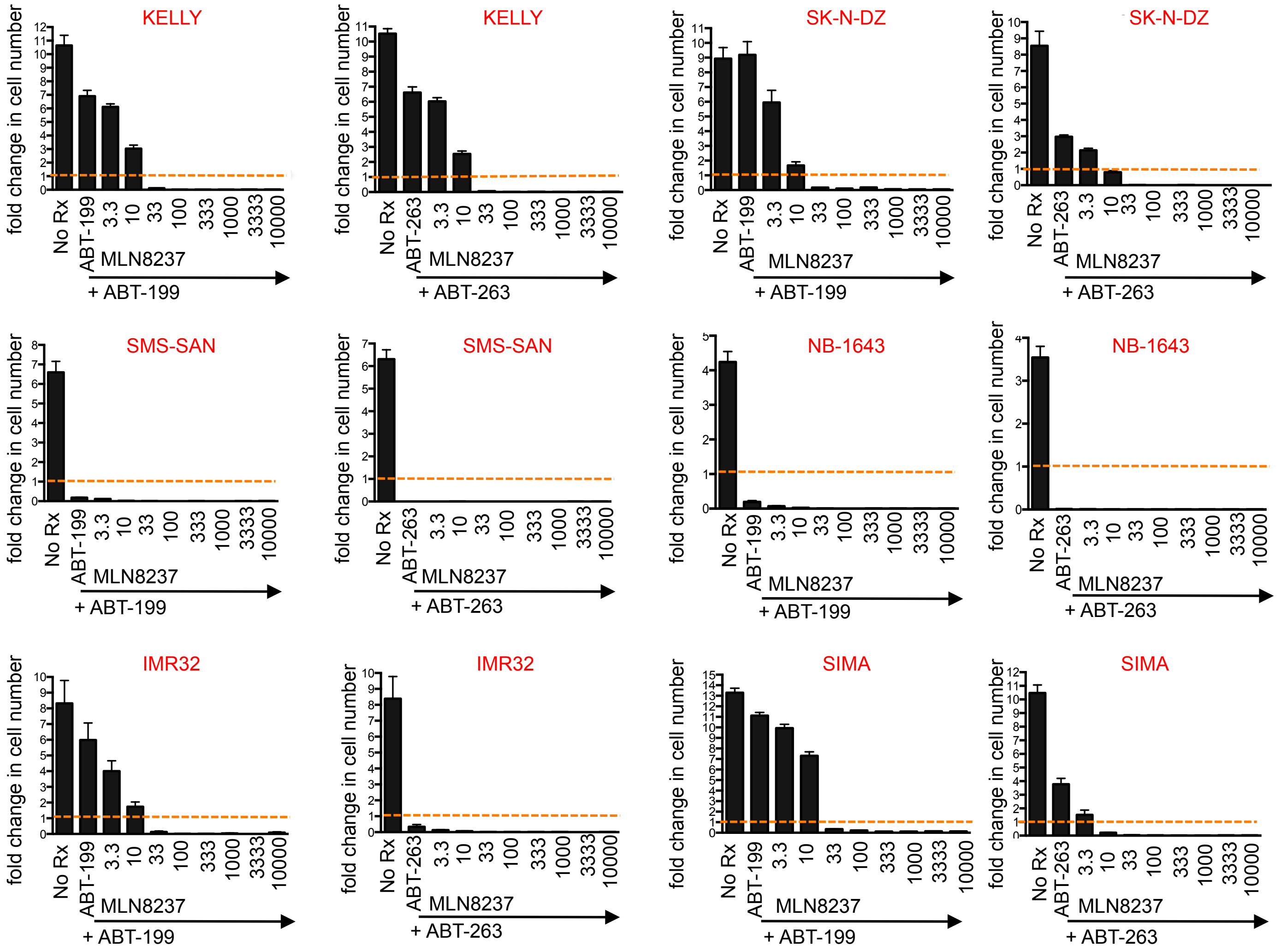

KELLY
SK-N-DZ
SMS-SAN
NB-1643
IMR32
SIMA
fold change in cell number
No Rx
ABT-199
ABT-263
3.3
10
33
100
333
1000
3333
10000
MLN8237
+ ABT-199
+ ABT-263

F

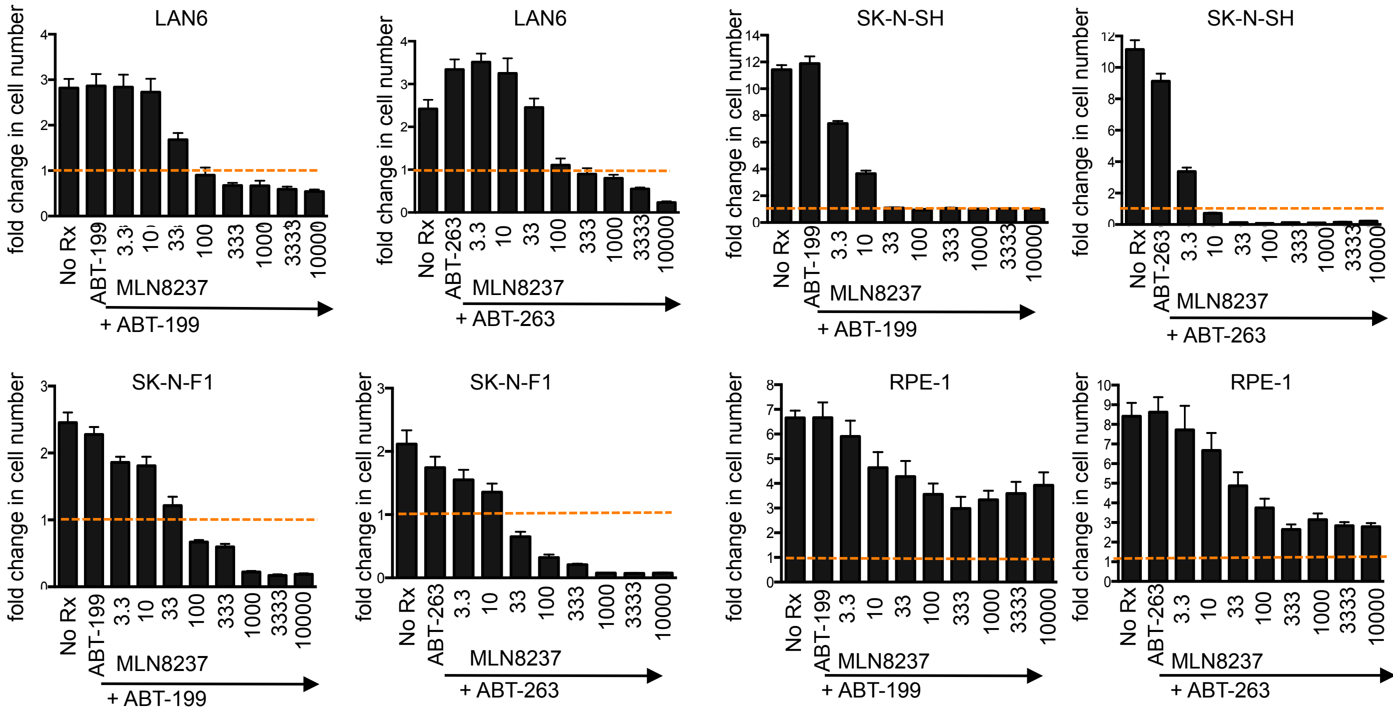

LAN6
SK-N-SH
SK-N-F1
RPE-1
fold change in cell number
No Rx
ABT-199
ABT-263
3.3
10
33
100
333
1000
3333
10000
MLN8237
+ ABT-199
+ ABT-263

Figure S6 (related to Figure 5). MCL-1 is critical in MLN8237/ABT-199 combination-mediated toxicity.
(A) SK-N-DZ cells engineered to overexpress GFP or MYCN were treated for 24 hr with no drug (No Rx) or the combination of 100 nM MLN8237 and 1 μM ABT-199 and lysates were probed for the indicated antibodies. **(B)** SK-N-BE(2) cells treated with control siRNA (siSC) or siMCL-1 (left panel) or siBIM (right panel) were lysed and assayed for knockdown efficiency. Figure is related to main Figure 5C.
(C) SK-N-DZ cells were treated with siSC or siBIM, and cells were treated with the combination and assayed for apoptosis 48 hr later (left). FACS apoptosis is presented as amount of apoptosis minus each condition minus no treatment scrambled control. Error bars are +S.E.M. (n=3). Insets, Western blots of scrambled (Sc) or BIM siRNA-treated cells (right).
(D) Lysates from cells treated for 24 hr were immunoprecipitated with BIM or an IgG control (left) or not immunoprecipitated (Whole Cell Lysates, right) (5% of immunoprecipitated proteins) and probed with the indicated antibodies. Ratio of quantified band intensities of MCL-1 complexed to MCL-1/BIM that was pulled down, for the indicated treatments.
(E,F) *MYCN*-amplified neuroblastoma cell lines (E) or *MYCN*-wild-type neuroblastoma cell lines or the RPE-1 cell line (F) were treated for 6 days with 1 μM ABT-199 or 1 μM ABT-263 in the presence or absence of increasing concentrations of MLN8237 and viability was determined. Error bars are +S.E.M. (n=3-4).

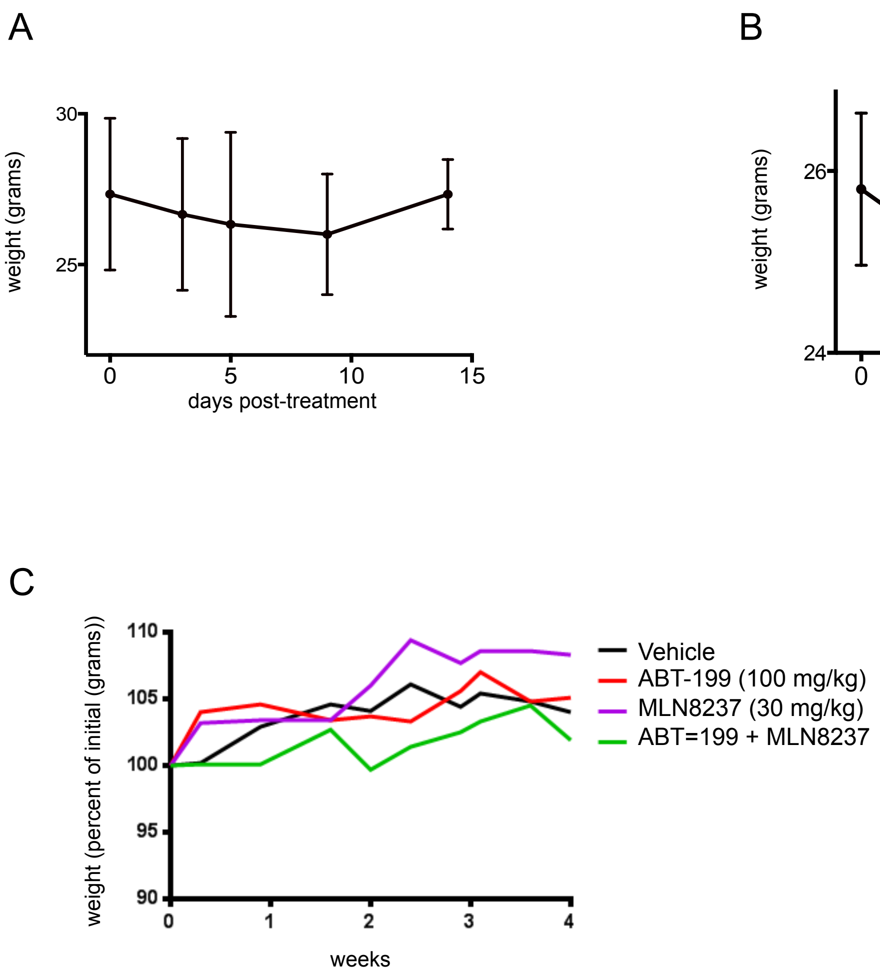

Figure S7 (related to Figure 6 and 7). No weight loss in mouse models treated with the combination.
(A,B) Weights of human xenograft-bearing mice (A, Kelly) and (B, SK-N-SH) from the in vivo experiments. Error Bars are -/+ S.D.
(C) Weights of PDX-bearing mice from the in vivo experiment.

Supplemental Experimental Procedures

Cell Lines

The cell lines in this study were from the Center for Molecular Therapeutics at Massachusetts General Hospital, The Children's Hospital of Philadelphia and Massachusetts General Hospital. KELLY, SIMA, IMR32, NB-1643, SMS-SAN, RPE-1, NBL-S, LAN6, SK-N-F1, SK-N-BE(2), SK-N-DZ, CAL-51, HCC2935, DU4475, and PC9 cells were cultured in RPMI (the Lonza Group) with 10% FBS (Seradigm) in the presence of 1μg/ml penicillin and streptomycin. HCC827 cells were cultured in RPMI with 5% FBS in the presence of antibiotics. CHP-212, SK-N-SH, and SK-N-AS cells were grown in DMEM/F12 (HyClone Laboratories, Inc.) with 10% FBS in the presence of 1μg/ml penicillin and streptomycin. 293T cells were grown in in DMEM with 10% FBS in the presence of 1μg/ml penicillin and streptomycin.

The Genomics of Drug Sensitivity in Cancer

The website for publically accessible drug-sensitivity data (www.cancerRxgene.org) contains the complete drug sensitivity profiles of the 130 drugs tested as well as the efficacy of different drugs compared between 26 *MYCN*-amplified cancers (including 20 neuroblastomas) and the remainder of the cancer cell lines (*MYCN*-wild-type). Table S1 is derived from this dataset and has been modified from the downloadable data.

Antibodies and Reagents

The antibodies used for Western blot analyses were n-Myc (MYCN) (cat #9405), β-Actin (cat #4967), phospho-Erk (cat #9101), phospho-GSK-3α/β(cat #9331), eIF4G (cat #2469), eIF4E (cat #2067), phospho-4E-BP1 (cat #2855), total 4E-BP1 (cat #9452) cleaved PARP (cat #5625), phospho-Aurora A/B/C (cat #13464), total aurora A (cat #12100), and Bim (cat #2933); all these antibodies were from Cell Signaling Technology (Beverly, MA). n-Myc (sc-53993), MCL-1 (sc-819) and normal Rabbit IgG from Santa Cruz Biotechnology (Dallas, TX) were used. The NOXA antibody (product # MA1-41000) was from Pierce-Thermo Scientific (Waltham, MA). GAPDH or β-Actin antibody were used as loading controls. The Protein A beads were from GE Healthcare (Little Chalfont, United Kingdom). The reagents used for apoptosis assay were Annexin V-Cy5 reagent (cat #1013-200) from BioVision (Milpitas, CA), propidium iodide (cat #51-66211E) from BD Biosciences (San Jose, CA), and Guava Nexin Reagent (part #4700-1140) from Millipore. Reagents used for siRNA experiments were Opti-MEM (cat #31985-070) from Life Technologies/Thermo Fisher (Waltham, MA) and HiPerfect Transfection Reagent (cat # 301705) from Qiagen (Valencia, CA). MLN8237, MLN0128 and ABT-199 were from Abmole (Houston, TX).

Western Blotting and Immunoprecipitation

Cell lines, tumors from traditional human xenografts, and tumors from patient-derived xenografts were prepared and lysed in lysis buffer (20mM Tris, 150mM NaCl, 1% NP-40, 1 mM EDTA, 1mM EGTA, 10% glycerol, and protease and phosphatase inhibitors), incubated on ice for 10 min and centrifuged at 10,500 rpm for 10 min at 4 °C. Tumor lysates were homogenized with Tissuemiser (Fisher Scientific) in the lysis buffer described previously, incubated for 20 min on ice, and centrifuged at 10,500 rpm for 10 min at 4 °C. Equal amounts of the detergent-soluble lysates were resolved using the NuPAGE® Novex® Midi Gel system on 4% to 12% Bis-Tris Gels (Invitrogen), transferred to PVDF membranes (PerkinElmer) in between 6 pieces of Whatman paper (Fisher Scientific) set in transfer buffer from Biorad with 20% methanol, and following transfer and blocking in 5% non-fat milk in PBS, probed overnight with the antibodies listed above. Representative blots from several experiments are shown in the Figures. Chemiluminescence was detected with the Syngene G:Box camera (Synoptics). For immunoprecipitation, cells were lysed in the same buffer; 250ug to 500ug were incubated with either BIM antibody (500 ng), MCL-1 antibody (500 ng), or rabbit IgG (500 ng). As Cell Signaling antibody concentrations change from lot to lot, precise concentrations were verified by the company. Following the addition of 25uL of 1:1 PBS: pre-washed Protein A beads to the antibody/lysate mix, samples were incubated with rotating motion overnight. Equal amounts of extracts (10% of immunoprecipitated protein) were prepared in parallel. For the immunoprecipitations, all antibodies from each panel were run on the same gel for precise comparisons.

Apoptosis
Cells were seeded in triplicate at roughly 30-40% confluency and treated for the indicated time with the indicated treatments, with no-treatment controls in parallel. Apoptosis experiments staining with propidium iodide and Cy5-Annexin V, including data from Figures 3B and 5A and Figure S3B were analyzed on a BD LSR III (Becton Dickenson), with the exception of the NBL-S cells from Figure 3B (see below). Apoptosis was determined 72 hr after treatments in Figure 3B and 24 hr after treatments in Figure S3B-S4B, and 48 hr in Figure 5A. With the exception of the KELLY cells, which were quantified 24 hr later for the due to the quick death kinetics of the cells. For the apoptosis experiments from data in Figures 2A, 2B, 2H, 5C and Figures S3A and S7C and the NBL-S cells from Figure 3B, cells were stained with Guava Nexin (Millipore) according to the manufacturer's protocol, and subsequently analyzed on a Guava easyCyte FACS machine (Millipore). For all apoptosis experiments staining with propidium iodide and Annexin, the number of cells in quadrants II and IV (Annexin V positive) were counted as apoptotic.

Cell Cycle
Cells were seeded in triplicate at roughly 30-40% confluency and treated for 24 hr, with no-treatment controls in parallel. The cells were trypsinized, washed and re-suspended with 0.1%Triton in PBS. The cells were then stained with Propidium Iodide, incubated for 20 min at 37 degrees °C and immediately analyzed on a BD LSR III for cell cycle profiles (Becton Dickenson, Figure S4A-S4B) or a Guava easyCyte FACS Machine (Figure S5D-S5E).

Cell Viability
For the long-term (six-day) ABT-199/MLN8237 and ABT-263/MLN8237 Cell Titer-Glo experiments in Figures S6E-S6F, sparsely seeded cells in two parallel 96-well flat bottom black plates were either 1) treated with 50 μL of CellTiter-Glo (Promega) the next day and immediately frozen ("day 0 plate"), or 2) treated the next day with drug. Following six days of continuous drug treatment at 37 degrees and 5% atmospheric CO_2, 50 μL of CellTiter-Glo was added to cells and immediately frozen. Cells were then thawed on a rocker with motion (Day 0 plate and treatment plate), and upon thawing, were read on a Centro LB 960 microplate luminometer (Berthold Technologies) according to the Promega protocol. The quantification of cells from the day 0 plate was used to determine the total cell growth number over the experiment. Cells were seeded in at least triplicate. For the 72 hr viability assays including data from Figures 2G, 2I, and 4C, sparsely seeded cells were plated in a flat-bottom black 96-well plate and the next day the indicated drug(s) at the indicated concentration(s) were added. 72 hr later, CellTiter-Glo (Promega) was added per well and read on the Centro LB960 microplate luminometer according to the Promega protocol. For the five-day crystal violet experiments, 100,000 cells were seeded (300,000 for the SMS-SAN and NB1643 cells) and the next day cells were treated with nothing, 10 nM MLN8237, 1 μM ABT-199 or the combination and five days later viable cells were stained with 0.1% crystal violet (Sigma-Aldrich) as shown in Figures 5F-5G.

RNA extraction and quantitative (Q) RT-PCR
RNA was isolated from cultured cells grown at sub-confluency using the Zymo Quick-RNA MiniPrep kit (Zymo Research), and RNA was reverse-transcribed to from cDNA molecules using cDNA synthesis kit superscript II (Invitrogen, Carlsbad, CA) on a 7500 Fast Real-Time PCR System (Life Technologies). The number of NOXA, MCL1 and β-ACTIN molecules was monitored in real time on a GENEAMP PCR System 9700 (Life Technologies) by measuring the fluorescence increases of SYBR Green (Roche). The primers for *PMAIP1* Forward were: 5′-GCTGGAAGTCGAGTGTGCTA-3′ and *PMAIP1* Reverse 5′-CCTGAGCAGAAGAGTTTGGA-3; *MCL1* Forward (5'-GGGCAGGATTGTGACTCTCATT-3') and *MCL1* Reverse (5'-GATGCAGCTTTCTTGGTTTATGG-3'); *ACTB* Forward (5'-GGCATGGGTCAGAAGGATT-3') and *ACTB* Reverse (5'-AGGATGCCTCTCTTGCTCTG-3'). To determine relative abundance of *PMAIP1* and *MCL1* in relation to *ACTB* , the Delta-Delta CT (cycle threshold) method was utilized.

siRNA Experiments

For the short-interfering (si)RNA experiments, siRNA designed against *MCL-1, MYCN, BIM,* and *AuroraA* and scrambled control oligos were used at a concentration of 50nM and transfected with Hiperfect reagent (Qiagen). In general, cells were plated in antibiotic-free media to achieve next day confluency of roughly 60%. Next, 75 μL of Hiperfect was added to 750 μL of OPTI-MEM in a 1.5 mL eppendorf tube. In parallel, scramble siRNA was added to 750 μL of Opti-MEM in a second, separate 1.5 mL eppendorf tube. Following gently mixing and incubation for ten minutes, the tubes were combined, and again, mixed gently. Following an additional ten min incubation time, the Hiperfect-siRNA mix was added to the appropriate cells. 24 hr later, the cells were re-seeded in 6-well plates, and the next morning were either (1) treated with the appropriate drug for eventual apoptosis determination, (2) lysed for Western blot analysis, or (3) collected for RNA isolation. The siRNA against *MCL-1*, *NOXA* and *Aurora A* were from Dharmacon, and the *BIM*, *MYCN* and scramble control siRNA were from Qiagen. The knockdowns for the scrambled, NOXA and MYCN, for example presented in Figure S2A-S2E, were performed within the same experiments. For the SK-N-BE(2) and SK-N-DZ cells, presented in Figure 5C and Figure S6B-S6C, the knockdowns for the scrambled, MCL-1 and BIM were performed within the same experiments. Apoptosis was measured 48 hours following drug additions in Figure 5C.

shRNA Experiments

For the short-hairpin (sh)RNA experiments, shRNA against NOXA (Hata et al., 2014) and MYCN (Sigma-Aldrich), MISSION shRNA (clone ID:NM_005378.3-230s1c1) were utilized. shRNA designed against a scramble sequence (MISSION pLKO.1-shRNA control plasmid DNA) served as the control. The shRNAs each have a pLKO.1 puromycin-resistant vector backbone and served as the basis for cell selection in puromycin following infection. Cells were transduced with plasmid-containing viral particles and viral particles were generated in 293T cells and collected over 48 hr.

MYCN and MCL-1 Overexpression

The GFP-IRES-MCL-1 pLENTI plasmid and pLENTI-GFP control were previously described (Faber et al., 2014). pMXs-hu-N-Myc (Nakagawa et al., 2010) was a gift from Shinya Yamanaka (Addgene plasmid #50772), and cloned into the pLENTI backbone to form pLENTI-MYCN.

Cap-dependent translation assay

Cells were lysed in the same buffer as used for Western blotting experiments. 30 μL of m7-GTP beads (Jena Bioscence, Germany) were added to an equal amount of cellular lysates. Following overnight incubation at 4 degrees Celsius, the IP complexes were washed three times in lysis buffer, boiled, and ran on a 4-12% BIS-TRIS gel (Invitrogen, Carlsbad, CA).

Global Protein Synthesis assay

Cells were pre-incubated with the indicated drugs, followed by the addition of 1μM puromycin for 30 min. Cells were lysed and subjected to western blot analysis with an anti-puromycin antibody, stripped for the loading control (β-ACTIN).

Combination synergy test

6000 cells /well were seeded in 96-well plates. A 8x8 dose matrix of either MLN8237 or ABT-199 was performed and cell viability was measured at the end of 48 or 72 hr using CellTiter Glo (Promega) according to Promega protocol. Excess over the Bliss values were calculated as a measure of synergy and are displayed in Figure S3C .

Protein Quantification

Bands, where indicated, were quantified on a Syngene using the GeneTools software program. Data is presented as the indicated ratios, with the control (No Rx) or the ABT-199 condition normalized to a value of “1”.

Supplemental Reference

Nakagawa, M., Takizawa, N., Narita, M., Ichisaka, T., and Yamanaka, S. (2010). Promotion of direct reprogramming by transformation-deficient Myc. Proceedings of the National Academy of Sciences of the United States of America *107*, 14152-14157.
